# The Wnt signaling networks in higher-grade meningiomas: from molecular pathogenesis to therapeutic innovation

**DOI:** 10.1186/s12935-026-04393-1

**Published:** 2026-07-24

**Authors:** Arathy S. Kumar, Olli Tynninen, Miikka Korja

**Affiliations:** 1 Department of Neurosurgery, Helsinki University Hospital and University of Helsinki, Haartmaninkatu 4, P.O. Box 320, Helsinki, FI- 00029 Finland; 2 Department of Pathology, Helsinki University Hospital and University of Helsinki, Helsinki, Finland

**Keywords:** High-grade meningiomas, NF2/Merlin, Wnt signaling pathway, β-catenin, Inhibitors, Therapeutic targeting

## Abstract

Meningiomas are the most frequently reported primary brain and other central nervous system tumors and account for more than one-third of all primary CNS tumors. High-grade meningiomas (WHO grades 2 and 3) are associated with significantly poorer prognosis and a higher risk of recurrence than grade 1 tumors, despite surgery and radiotherapy. Although several genetic and epigenetic alterations, including *NF2* inactivation and related molecular drivers, have been identified in these tumors, effective systemic treatment options remain limited. Increasing evidence indicates that the Wnt/β-catenin signaling pathway is altered in meningiomas, as demonstrated by dysregulated expression of β-catenin, pathway regulators, and downstream transcriptional activity in different studies. Mechanistic studies in meningioma models show that Merlin S13 dephosphorylation, rather than Merlin loss alone, can attenuate inhibitory Merlin–β-catenin interactions, promote β-catenin-dependent Wnt signaling, and support tumor growth in *NF2*-wildtype meningiomas. Because the Wnt pathway regulates key processes such as proliferation, differentiation, apoptosis resistance, and tumor microenvironment interactions, its dysregulation may contribute to meningioma progression, particularly in biologically aggressive or higher-grade tumors. A better understanding of these mechanisms may help identify more rational therapeutic approaches for aggressive meningiomas. However, their clinical relevance will depend on whether candidate strategies can achieve adequate tumor exposure while maintaining acceptable systemic and CNS safety.

**Supplementary Information:**

The online version contains supplementary material available at https://doi.org/10.1186/s12935-026-04393-1.

## Introduction

**Fig. 1:**
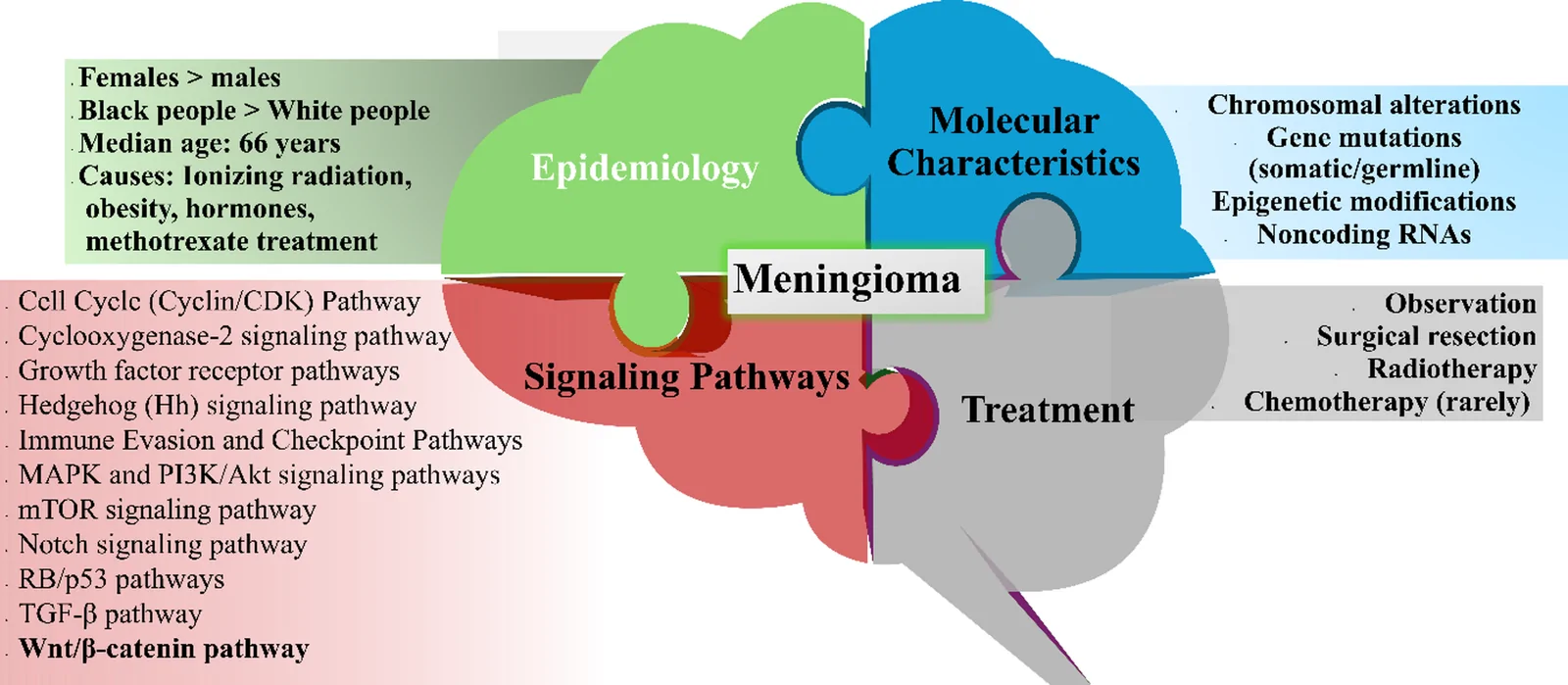
Epidemiological, molecular and therapeutic characteristics of meningiomas

Meningiomas are the most frequently reported primary brain and other central nervous system tumor histopathology in the United States, accounting for 42.6% of all primary brain and other CNS tumors diagnosed during 2018–2022 [1]. Meningiomas have historically been linked to meningothelial arachnoid cap cells [2–4], but this classical view does not fully explain their histological, molecular, anatomical, and clinical heterogeneity. Recent evidence from developmental biology, single-cell and spatial transcriptomics, proteomics, and experimental models supports a more heterogeneous model of meningioma ontogeny. In this model, the cell of origin is unlikely to be universal and may involve early meningeal progenitor populations, including fibroblast-like or mesenchymal progenitor candidates that vary with anatomical location and embryological lineage [4]. This heterogeneity may partly reflect regional differences in meningeal embryology, with the spatial distribution of genotypically distinct meningiomas potentially related to differential neural crest and mesodermal contributions across cranial meningeal regions [5]. Beyond demographic and environmental risk factors, including older age, female sex (possibly reflecting hormonal influences), Black/African American race, and exposure to ionizing radiation (particularly childhood cranial irradiation), elevated body mass index is also a well-established contributor to meningioma risk (Fig. 1) [6, 7]. In contrast, a history of atopic conditions, including allergies, eczema, and elevated serum IgE, has consistently been associated with reduced risk, implicating a potential protective effect of immune surveillance [7].

The World Health Organization (WHO CNS5) classifies meningiomas into three grades based on histopathological features and molecular characteristics as grade 1 (benign), grade 2 (atypical), and grade 3 (anaplastic/malignant) [8–12]. While most grade 1 meningiomas are slow-growing and associated with favourable outcomes, grade 2 and especially grade 3 meningiomas show higher recurrence risk and poorer clinical behaviour [13]. This higher-risk behaviour is reflected partly by WHO CNS5 grading criteria: brain invasion, increased mitotic activity, and atypical histological features support grade 2 designation, whereas frank anaplasia, very high mitotic activity, *TERT* promoter mutation, or homozygous *CDKN2A/B* deletion defines grade 3 disease [9, 13]. Treatment of meningiomas is individualized and primarily relies on observation, surgical resection, and radiotherapy depending on tumor size, location, growth behaviour, and patient factors [14]. Gross-total resection is preferred when it can be achieved safely, and radiotherapy is commonly used for incompletely resected or inoperable tumors [15]. Systemic therapy has no established standard role in meningioma management. Agents evaluated to date, including hydroxyurea, interferon-α, somatostatin analogues, targeted agents, and anti-angiogenic approaches, have generally produced limited and variable activity, with disease stabilization more common than objective tumor regression [14]. For recurrent or progressive disease, enrollment in clinical trials remains recommended when available [16].

Molecular studies have identified recurrent genetic and epigenetic alterations in meningioma, including *NF2* loss, non-*NF2* driver mutations, copy-number changes, DNA methylation changes, histone modification, and altered miRNA expression [3]. Copy-number alteration burden increases in higher-grade meningiomas, and specific chromosomal losses or complex cytogenetic profiles are associated with recurrence risk and shorter recurrence-free survival [17–19]. Convergent molecular analyses identify biologically distinct subtypes, including an *NF2*-deficient, chromosomally unstable Type C/MenG C subgroup associated with frequent recurrence [20]. DNA methylation-based and integrated molecular-morphologic classifiers further improve recurrence-risk stratification beyond histology alone [21–23]. Together, these advances have refined molecular diagnosis, prognostication, and pathway-informed therapeutic thinking in meningioma, particularly for aggressive or recurrent disease [24, 25]. This molecularly informed view also provides a framework for examining signaling pathways that may contribute to aggressive tumor behavior. In this context, Wnt signaling has been implicated among altered signaling programs in meningiomas, with expression [26] and proteomic studies [27] reporting Wnt-related changes associated with higher-grade disease and broader brain-tumor literature supporting its biological relevance [28]. Proteomic analysis in a cohort of meningiomas revealed enrichment of the Wnt pathway, particularly in non-radiated meningiomas [29]. Originally identified for its role in embryogenesis and tissue homeostasis, aberrant activation of the Wnt pathway has been implicated in numerous cancers, where it contributes to oncogenesis through modulation of cell cycle, migration, and stemness [30, 31]. Dysregulated Wnt signaling has been linked to poor prognosis in several non-meningioma cancers [32, 33]. However, its prognostic significance is context-dependent; WNT-activated medulloblastoma has a favourable prognosis, with 5-year survival often exceeding 90% [34–36]. Because Wnt signaling supports normal tissue homeostasis, systemic pathway inhibition can cause on-target toxicity. Evidence from other cancers suggests that Wnt-targeted therapy may be safer and more effective when used in rational combinations or delivered more selectively [37, 38], but whether these approaches will work in meningioma remains to be established. Although Wnt signaling has been proposed as a therapeutic target in brain tumors [28], meningioma-specific clinical validation remains lacking. Preclinical study suggests that Wnt signaling may contribute to meningioma biology in a context-dependent manner. In *NF2*-mutant models, ligand-mediated Wnt/β-catenin signaling has been linked to sensitivity to CBP/β-catenin inhibition [39]. Complementing this, work in Merlin-intact meningiomas showed that the phosphorylation status of Merlin at S13 regulates its inhibitory interaction with β-catenin; S13 dephosphorylation attenuates this interaction, activates Wnt signaling, and promotes tumor growth [40]. Together, these findings suggest that Wnt pathway activation in meningioma may emerge from distinct molecular contexts, including NF2 loss and post-translational regulation of Merlin, supporting biomarker-guided evaluation of Wnt-directed therapeutic strategies. However, clinical translation will require reliable markers of Wnt pathway activity, careful patient selection, and safety-aware therapeutic design because systemic Wnt inhibition can affect normal tissue homeostasis [33, 41].

Wnt-pathway reviews of intracranial meningioma describe altered expression or activity of several pathway components, including Frizzled receptors, *CTNNB1*/β-catenin, *CCND1*, and *ENC1*, but the strength and grade-specificity of these findings vary across studies [42]. Loss-of-function alterations in tumor suppressors such as *NF2*, which encodes Merlin, can relieve restraints on Wnt/β-catenin signaling and promote tumor growth in several tumor types, including sporadic meningiomas [43–45]. In meningiomas, Vasudevan et al. demonstrated that aggressive tumors are characterized by a FOXM1/Wnt signaling axis [46]. Their comprehensive molecular profiling identified FOXM1 as a key transcription factor driving proliferation and poor clinical outcomes, while immunohistochemistry revealed cytoplasmic and occasional nuclear β-catenin, indicating Wnt pathway activation. These findings support a FOXM1/Wnt-associated proliferative program linked to mitotic gene expression and aggressive clinical behavior [46]. Recent evidence also links Meflin/*ISLR*, a meningeal cell-associated marker, with Wnt-related biology in meningioma progression. Higher *ISLR* expression was associated with higher tumor grade and recurrence, and Meflin-high tumor cells showed enrichment of the “WNT ligand biogenesis and trafficking pathway”, with WNT6 identified as the specifically upregulated WNT ligand [47]. A recent case report described a histologically WHO grade 1 convexity meningioma in a young woman with osteosclerosis and a germline *AMER1/WTX* mutation. AMER1/WTX is a tumor-suppressor protein involved in canonical Wnt/β-catenin regulation, suggesting a possible but still unproven link between altered *AMER1* and meningioma biology [48]. Furthermore, crosstalk between Wnt signaling and other oncogenic pathways, including PI3K/Akt, Hippo/YAP, and FOXM1, creates a complex network that sustains cancer progression [49, 50]. *NF2* loss or mutation confers notable radioresistance in high-grade meningiomas [51, 52]. Parallel evidence implicates Wnt-driven programs in radioresistance across diverse tumor types [53]. Valproic acid (VA), a histone deacetylase (HDAC) inhibitor, has been reported to increase radiosensitivity in meningioma stem-like cells [54]. However, HDAC-related effects may differ across tumor contexts. In breast cancer stem-like cells, valproic acid has been shown to promote de-differentiation through Wnt/β-catenin signaling [55], suggesting that drug effects on Wnt-associated biology should not be extrapolated directly from one tumor type to another. Therefore, understanding the molecular underpinnings of Wnt pathway alterations in higher grade meningiomas (grade 2 and 3) and its role in radioresistance and potential therapy resistance is essential to develop targeted therapeutic strategies.

This narrative review summarizes current evidence on Wnt signaling in meningioma. Relevant studies were identified through PubMed, Scopus, Web of Science, and Google Scholar. Search terms included “meningioma,” “WHO grading,” “molecular classification,” “DNA methylation,” “Wnt signaling,” “Wnt/β-catenin,” “Wnt/non canonical pathway”, “Frizzled/*FZD*,” “*NF2*/Merlin,” “recurrence,” “prognosis,” “targeted therapy,” and “Wnt inhibitor.” In PubMed, relevant Medical Subject Headings (MeSH) were incorporated where available to improve search sensitivity and consistency. Priority was given to original, translational, molecular-classification, clinical, and review articles. Evidence from non-meningioma cancers was included only when it was directly relevant to Wnt biology, therapeutic targeting, or toxicity.

## WHO classification and molecular subtypes

### WHO classification

**Fig. 2:**
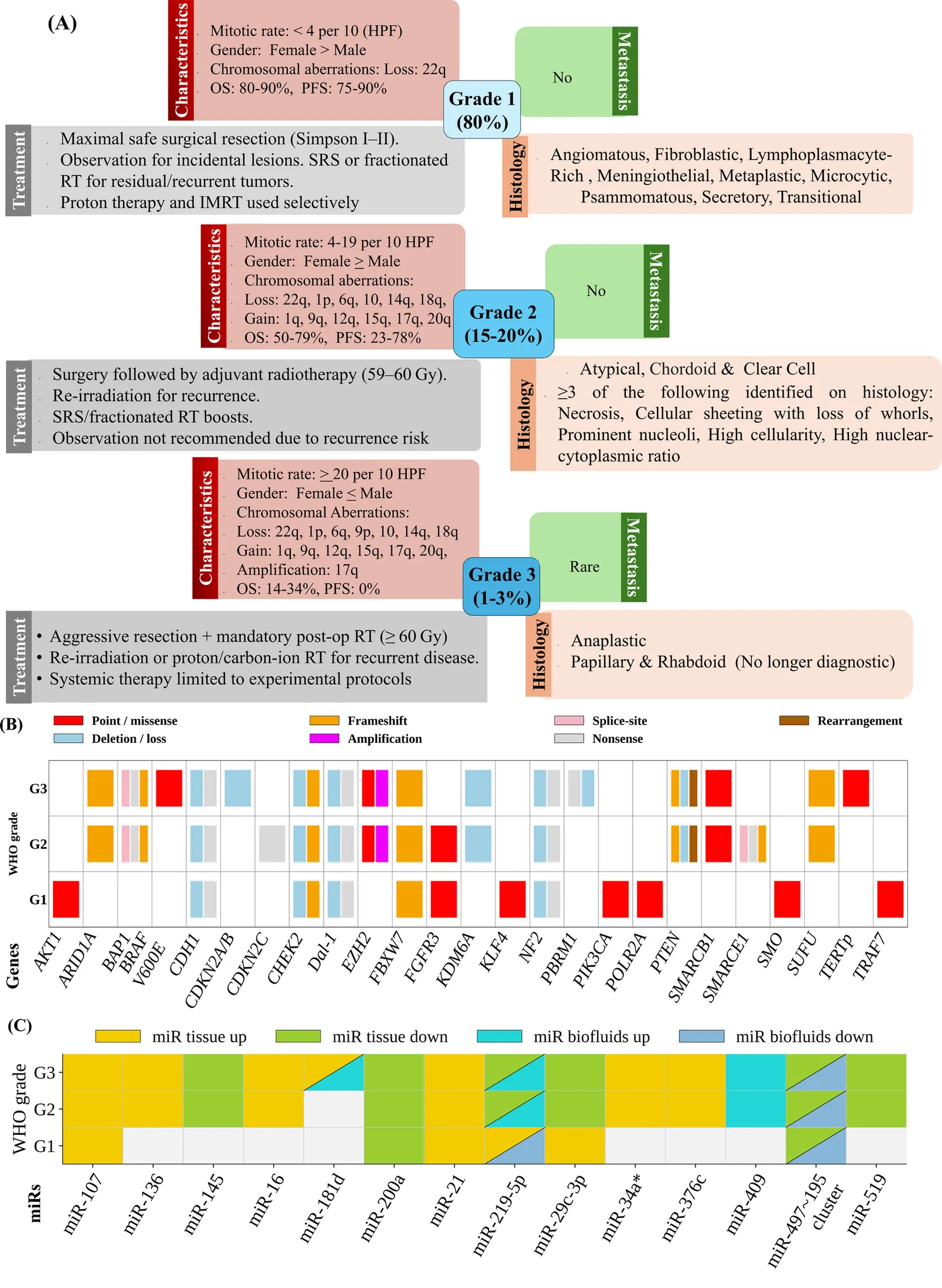

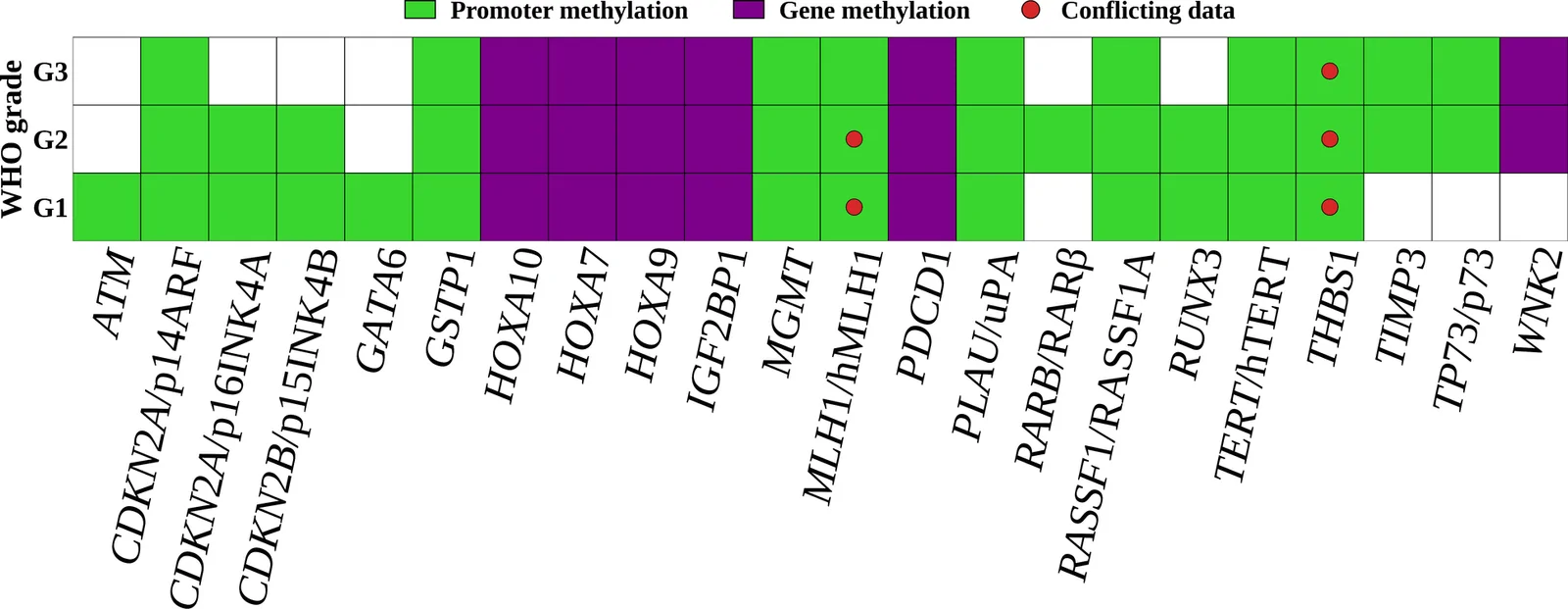
WHO classification and molecular features of meningioma. (**A**) Histological and clinicopathological characteristics of WHO grade 1, grade 2, and grade 3 meningiomas. (**B**) Representative reported genetic alterations across meningioma grades, compiled and redrawn from data summarized by Wang JZ, et al., 2024 [61]. (**C**) Dysregulated microRNAs reported in meningioma, compiled and redrawn from data summarized by Beylerli O, et al., 2024 [71]. (**D**) Promoter and gene methylation alterations associated with meningioma biology, compiled and redrawn from published data summarized in [62] and supporting original studies [72–85]. (AKT - Protein kinase B; ATM - ATM serine/threonine kinase; BAP1 - BRCA1 associated deubiquitinase 1; CDKN2A/B - Cyclin dependent kinase inhibitors 2A and 2B; CDKN2A/p14ARF - Cyclin dependent kinase inhibitor 2A/cyclin-dependent kinase inhibitor 2A isoform p14ARF; CDKN2A/p16INK4A - Cyclin dependent kinase inhibitor 2A/cyclin-dependent kinase inhibitor 2A isoform p16INK4A; CDKN2B/p15INK4B - Cyclin dependent kinase inhibitor 2B/p15INK4B protein; G1 - Grade 1 meningioma; G2 - Grade 2 meningioma; G3 - Grade 3 meningioma; GATA6 - GATA binding protein 6; GSTP1 - Glutathione S-transferase pi 1; HOXA7 - Homeobox A7; HOXA9 - Homeobox A9; HOXA10 - Homeobox A10; IGF2BP1 - Insulin like growth factor 2 mRNA binding protein 1; IMRT - Intensity-modulated radiotherapy; KLF4 - KLF transcription factor 4; MGMT - O-6-methylguanine-DNA methyltransferase; miR - MicroRNA; MLH1/hMLH1 - MutL homolog 1/human MutL homolog 1; NF2 - NF2, moesin-ezrin-radixin like (MERLIN) tumor suppressor; OS - Overall survival; PDCD1 - Programmed cell death 1; PFS - Progression-free survival; PIK3CA - Phosphatidylinositol-4,5-bisphosphate 3-kinase catalytic subunit alpha; PLAU/uPA - Plasminogen activator, urokinase/urokinase-type plasminogen activator; RARB/RARβ - Retinoic acid receptor beta; RASSF1/RASSF1A - Ras association domain family member 1/Ras association domain family member 1 isoform A; RUNX3 - RUNX family transcription factor 3; SMO - Smoothened, frizzled class receptor; SRT - Stereotactic radiotherapy; TERT/hTERT - Telomerase reverse transcriptase/human telomerase reverse transcriptase; TERTp - Telomerase reverse transcriptase promoter; THBS1 - Thrombospondin 1; TIMP3 - TIMP metallopeptidase inhibitor 3; TP73/p73 - Tumor protein p73; TRAF7 - TNF receptor associated factor 7; WHO - World Health Organization; WNK2 - WNK lysine deficient protein kinase 2.)

The World Health Organization (WHO) classification system for meningiomas has evolved over decades to incorporate both histopathological and molecular features, aiming to improve prognostication and guide clinical management. The latest 2021 WHO classification of central nervous system tumors divides meningiomas into three grades. Grade 1 (benign) meningiomas comprise approximately 80% of meningiomas and include nine histological variants (Fig. 2). They generally exhibit low mitotic activity (less than 4 mitoses per 10 high-power fields (HPF)), lack brain invasion, and have favourable clinical outcomes. Grade 2 (atypical) tumors represent 15–20% of meningiomas, these tumors show increased mitotic activity (4–19 mitoses per 10 HPF), brain invasion, and include three specific histological variants, namely atypical, clear cell and chordoid meningiomas. These features correlate with a higher risk of recurrence and more aggressive clinical behaviour compared to grade 1 tumors. Grade 3 (anaplastic/malignant) meningiomas account for 1–3% of cases with marked mitotic activity (≥ 20 mitoses per 10 HPF), frank anaplasia (malignant morphology), and may show papillary or rhabdoid characteristics. Papillary or rhabdoid morphology alone should not be considered sufficient for grade 3 designation under WHO CNS5 unless grade-defining criteria are met [8, 9]. Grade 3 meningiomas carry poor prognosis with high recurrence rates and increased mortality [8–11] (Fig. 2).

### Molecular subtypes and epigenetic profiles

Beyond histological morphology, the 2021 WHO update emphasizes integrating molecular alterations for precise tumor grading and prognostication. Molecular markers such as *TERT* promoter mutations and *CDKN2A/B* homozygous deletions are recognized molecular criteria for CNS WHO grade 3 designation and are increasingly incorporated into diagnostic risk assessment [9, 56–59]. cIMPACT-NOW Update 8 clarified the role of molecular risk parameters in meningioma grading. It reaffirmed WHO CNS5 grade 3 criteria, including oncogenic *TERT* promoter variants and homozygous *CDKN2A/B* deletion. It also recommended CNS WHO grade 2 assignments for tumors with grade 1 morphology when 1p deletion co-occurs with 22q deletion and/or *NF2* oncogenic variants. In selected cases, copy-number, DNA methylation, and RNA-based profiling may further refine grading and risk assessment [60–62]. Next-generation sequencing has revealed distinct genetic patterns in different grades of meningiomas [63]. Lower-grade tumors (grade 1) frequently harbor mutations in *TRAF7*,* KLF4*,* AKT1*, and *SMO*, often defining benign, skull-base subgroups. In contrast, higher-grade meningiomas (grade 2–3) are predominantly driven by *NF2* mutations and/or chromosome 22q loss and often accumulate recurrent copy-number alterations, correlating with more aggressive behaviour and higher recurrence risk. These findings highlight how genetic alterations align with tumor grade, location, and clinical outcome, providing a foundation for molecular classification and targeted therapeutic strategies [63]. For instance, loss of *NF2*/Merlin disrupts contact inhibition and activates multiple oncogenic pathways, including Hippo/YAP, Wnt/β-catenin signaling and PI3K/AKT/mTOR, collectively promoting proliferation, survival, and tumor progression in meningiomas and other *NF2*-deficient tumors [64, 65]. In *NF2*-deficient mouse embryonic fibroblasts, loss of *NF2* increased canonical Wnt/β-catenin signaling and that Rac1-dependent Wnt signaling was required for loss of contact inhibition of proliferation [66]. Understanding these molecular subtypes is crucial for accurate risk stratification, and tailoring treatment strategies, particularly in grade 2 and 3 meningiomas, where effective systemic options remain very limited [14, 16, 67].

Recent DNA methylation profiling studies have identified molecular subgroups of meningioma that provide prognostic information beyond histological grade [22, 23, 68]. Sahm et al. and others demonstrated DNA methylation-based classification systems for meningioma with unfavorable methylation subgroups in grade 2 and 3 meningiomas, enriched for copy number aberrations, including losses of 1p, 6q, 14q, and 18q, and these subgroups often correlate with reduced recurrence-free survival [21, 22, 69]. Similarly, Nassiri et al. showed that integrative molecular groups, particularly MG3 and MG4, are characterized by a higher burden of chromosomal copy-number losses and significantly worse recurrence-free survival, and that these molecular groups provide prognostic stratification within each WHO grade [70]. Histone modifications also contribute to epigenetic dysregulation in high-grade meningiomas. Trimethylation of histone H3 at lysine 27 (H3K27me3) is a repressive chromatin mark involved in transcriptional silencing.

In meningioma, loss of H3K27me3 has been associated with adverse outcome in some cohorts, but its reproducibility and value as a reliable grade 3 biomarker remain debated [86, 87]. In addition, in an atypical meningioma cohort study, diminished expression of the pro-apoptotic proteins CASP3 and BAX, coupled with upregulation of the anti-apoptotic proteins survivin and MDM2, is associated with increased risk of tumor recurrence. Although histone-modifying enzymes contribute to the regulation of these apoptosis-related proteins, their expression alone was not independently predictive of recurrence in this cohort [88]. Beyond histone post-translational modifications, high-grade and histologically aggressive meningiomas frequently harbor recurrent lesions in chromatin-regulating genes. The mammalian SWI/SNF chromatin-remodeling complexes (BAF/PBAF/ncBAF) are ATP-dependent chromatin remodelers that reposition or evict nucleosomes to modulate DNA accessibility [89]. Within the SWI/SNF complex, different components have been reported to be mutated in meningioma, which is detailed in a later section.

MicroRNA dysregulation adds another layer of epigenetic complexity (Fig. 2). MicroRNAs are small non-coding RNAs that regulate gene expression after transcription by promoting mRNA degradation or inhibiting translation. In meningioma, altered miRNA expression has been reported across tumor grades. Katar et al. found that miR-21 and miR-107 expression varied with histopathological grade [90], while miR-224 overexpression was associated with advanced grade, recurrence, increased proliferation, and reduced apoptosis [91]. Reduced expression of miR-34a, miR-195, and miR-145 has also been described in higher-grade or more aggressive meningiomas, with functional studies suggesting roles in proliferation, invasion, and apoptosis regulation [92–94]. MiR-200a negatively regulates β-catenin expression and Wnt/β-catenin signaling in meningioma models, supporting a role in tumor growth and differentiation-related pathways [71, 95]. Together, these studies support the role of DNA methylation signatures, histone modifications, and miRNA networks in defining the malignant potential of high-grade (2 and 3) meningiomas. These findings may help refine risk stratification and prioritize molecularly selected preclinical testing.

## Molecular genetics of high-grade meningiomas: implications for Wnt signaling

High-grade meningiomas (grade 2 and 3) exhibit a complex landscape of genetic alterations that contribute to their aggressive behavior, frequent recurrence [96] and radiation resistance [51, 52]. Understanding these molecular aberrations is essential for identifying the molecular pathways affected, which will help in designing targeted therapies for meningiomas [97]. Several signaling pathways have been investigated in meningioma progression, including Wnt/β-catenin signaling [98]. Although Wnt signaling regulates key cellular processes, its specific role in high-grade meningioma remains incompletely defined.

### *NF2*/Merlin loss

The most common genetic alteration in meningiomas, particularly in higher grades, is the inactivation or loss of the *NF2* gene located on chromosome 22q12.2, which encodes the tumor suppressor protein Merlin [17, 99, 100]. Germline *NF2* (neurofibromatosis type 2) mutations and somatic *NF2* loss in sporadic meningiomas represent distinct but convergent routes to Merlin inactivation [45]. Merlin negatively regulates several signaling pathways, including Wnt/β-catenin, by promoting β-catenin degradation through the upregulation of Axin1 and inhibiting its nuclear translocation in breast cancer and pancreatic cancer [101], though this mechanism has not been directly demonstrated in meningioma. Merlin also inhibits Wnt signaling by blocking LRP6 phosphorylation [43]. Merlin loss can alter Wnt/β-catenin-related signaling in several model systems [44], but the exact relationship between *NF2* loss, β-catenin stabilization, and Wnt output in meningioma appears context-dependent. In a large multi-institutional genomic profiling cohort of high-grade/progressive meningiomas, *NF2* mutations were reported in 60.1% and 69.3% in WHO grade 2 and 3 tumors respectively, supporting the association of *NF2*-mutant biology with aggressive meningioma subsets. *NF2*-mutant tumors were also more frequent in male than female patients, occurring in 64.0% versus 48.7% of cases, respectively [99]. Another genomic study found *NF2* or chromosome 22 alterations in 80% of high-grade meningiomas compared with 43% of low-grade tumors [17]. *NF2*-mutant meningiomas that progress in grade often show broad copy-number instability, including recurrent losses of 1p, 10, and 22q, suggesting that chromosomal instability may contribute to progression in a subset of *NF2*-driven tumors [102].

### *TERT* promoter mutations

Telomerase reverse transcriptase (TERT), the catalytic subunit of telomerase, sustains telomere length and allows cells to bypass replicative limits, supporting continuous division and the uncontrolled growth fueled by oncogenic signaling [103]. *TERT* promoter mutations in meningioma most commonly involve the recurrent C228T and C250T hotspot substitutions. They are more frequent in higher-grade tumors. Reported frequencies are 1.7%, 5.7%, and 20.0% in WHO grade 1, 2, and 3 meningiomas, respectively [104]. Another study reported p*TERT* mutations in 17.6% of atypical/anaplastic meningiomas, compared with < 5% of benign tumors [105]. Clinically, *TERT* promoter mutations are associated with shorter time to progression and poor prognosis [104, 106]. *TERT* promoter mutations in meningioma have been linked to increased TERT expression, telomerase activity, and indefinite in vitro growth in meningioma models [106]. In other cancers, TERT can interact with β-catenin and enhance Wnt target-gene transcription [107], but this mechanism remains unproven in meningioma.

The nuclear TERT primarily performs its telomere-maintaining canonical function, while the cytoplasmic and mitochondrial TERT can interact with signaling pathways such as Wnt/β-catenin signaling and NF-κB signaling [108]. Evidence from other cancers suggests that TERT may interact with β-catenin [109, 110], but this has not been conclusively demonstrated in meningiomas. Whether TERT–β-catenin interactions contribute to meningioma aggressiveness remains speculative and should be tested directly in meningioma models.

### *CDKN2A/B* deletions

Homozygous deletions of *CDKN2A/B*, which encode tumor suppressors p16^INK4A^ (and p14^ARF^, encoded by an alternative reading frame of *CDKN2A*) and p15^INK4b^ respectively, are recurrently reported in anaplastic/progressive meningiomas and contribute to aggressive tumor behavior [56, 59, 111]. The p16^INK4A^ protein inhibits CDK4 and CDK6, which prevents Rb phosphorylation and blocks G1/S cell-cycle progression. Prolonged activation of this pathway can promote cellular senescence. In contrast, p14^ARF^ inhibits MDM2-mediated ubiquitination and degradation of p53, thereby stabilizing p53 and supporting p53-dependent tumor-suppressive responses [112]. By forming a complex with CDK4 and CDK6, p15^INK4b^ prevents the activation of these kinases and controls cell cycle progression at the G1 phase [113]. Loss of these genes leads to deregulation of cell cycle checkpoints and increased proliferation [114, 115]. Heterozygous *CDKN2A/B* loss may also be relevant, as it has been linked to shorter recurrence-free survival in aggressive meningioma, including within the MenG C subgroup [116]. Direct cooperation between *CDKN2A/B* loss and Wnt signaling has not been shown in meningioma, although studies in skin [117] and colorectal cancer [118] suggest that cell-cycle regulators can interact with Wnt-driven programs. Given the molecular heterogeneity of aggressive and recurrent meningiomas, this possible interaction should be viewed as hypothesis-generating and may help guide future testing of rational combination strategies.

### Other genetic alterations

High-grade and progressive meningiomas show heterogeneous genomic alterations beyond *NF2* inactivation. Williams et al. identified biologically aggressive subclasses that included *NF2*-associated tumors with *CDKN2A/B* or *ARID1A* alterations, *NF2*-agnostic tumors with *TERT* promoter or *TP53* alterations, and *NF2*-wild-type tumors with *BAP1* or *PBRM1* alterations. Focal *DMD* deletions and alterations in *BAP1*,* PBRM1*,* ARID1A*, and *SMARCE1* therefore appear to define subsets of progressive meningioma rather than general features of all high-grade tumors [99]. *BAP1* loss is particularly associated with aggressive meningiomas showing rhabdoid histomorphology [119]. PBRM1, a component of the PBAF chromatin-remodeling complex, has been reported to show truncating mutations and homozygous deletion in papillary meningiomas [120], with loss-of-function alterations also described in an advanced meningioma case [121]. *ARID1A* loss-of-function alterations have been linked to recurrent meningioma and poorer prognosis [122], and a frameshift deletion has been described during acquisition of rhabdoid morphology [123]. *SMARCE1* loss-of-function mutations are enriched in clear cell meningioma, a WHO grade 2 subtype [124]. *SMARCE1*-deficient clear cell meningiomas show a distinct epigenetic and transcriptional profile. In transcriptomic pathway analysis, WNT signaling was among the gene-expression programs altered in *SMARCE1*-null tumors [125], although a direct mechanistic SMARCE1–Wnt axis has not yet been established in meningioma. SUFU, a negative regulator of Hedgehog signaling located at 10q24.32, has been implicated in meningioma biology through germline loss-of-function predisposition to familial multiple meningioma [126], and low-frequency SUFU mutations/focal deletions have also been reported in high-grade/progressive meningioma cohorts [99].

By contrast, *TRAF7*,* KLF4*,* SMO*, and *AKT1* mutations are more often seen in benign and some atypical meningiomas, where they define non-*NF2* molecular subgroups with characteristic anatomical and histological patterns [127, 128]. Evidence from other systems suggests possible connections between Wnt/β-catenin signaling and chromatin regulation, Hedgehog/SUFU signaling, AKT–GSK3β activity, and KLF4-dependent transcriptional control [129–136]. In meningioma, direct evidence linking all these alterations to Wnt signaling is still limited. Therefore, these links should be treated as priorities for experimental testing rather than as established mechanisms.

### Genetic heterogeneity and clinical implications

High-grade meningiomas are genetically heterogeneous, often harboring multiple mutations that collectively drive tumor aggressiveness. This heterogeneity complicates prognostication and therapeutic targeting [137]. These mutations and crosstalk promote oncogenic transcriptional programs leading to enhanced proliferation, invasiveness, and potential therapy resistance. Lucas et al. showed that high-grade meningiomas have marked spatial heterogeneity, with region-to-region differences in gene/protein programs and sub-clonal CNVs, while grade 1 comparators had fewer, less-diverse clusters and no divergent CNVs across regions. In matched primary-recurrent pairs, recurrence is characterized by expansion of sub-clones present at low levels in the primary, along with decreased immune and MAPK signals and increased PI3K–AKT signaling and proliferation. Within single tumors, adjacent grade 2 and grade 3 areas aligned with distinct CNV patterns and cell states, underscoring sampling bias. CRISPRi co-culture models further showed clone-specific drug sensitivities and that combination therapy can suppress mixed populations more effectively than single agent treatment [138]. Molecular genetic profiling may improve meningioma risk stratification and guide future personalized management [96].

## Canonical and non-canonical Wnt signaling pathways in grade 2 and 3 meningiomas

**Fig. 3:**
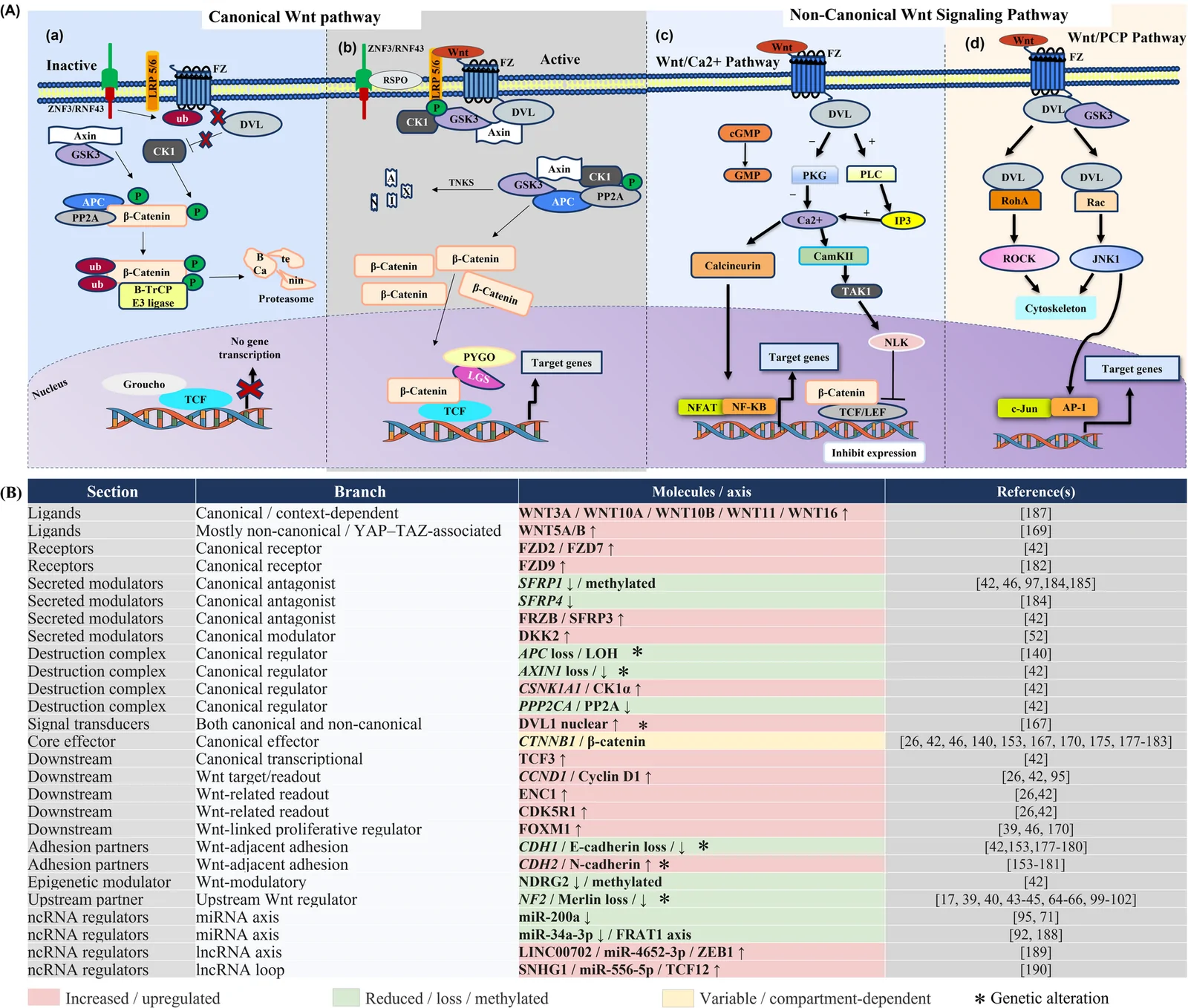
Schematic representation of canonical and non-canonical Wnt signaling pathways. (**A**) Canonical and non-canonical Wnt signaling branches (**B**) Reported Wnt-pathway mutations or alterations in meningioma. (APC - Adenomatous polyposis coli; CaMKII - Calcium/calmodulin-dependent protein kinase II; CK1α - Casein kinase 1 alpha; DVL - Dishevelled; FZD - Frizzled receptor; GSK3β - Glycogen synthase kinase 3 beta; IP3R - Inositol 1,4,5-trisphosphate receptor; JNK - c-Jun N-terminal kinase; LRP5/6 - Low-density lipoprotein receptor-related protein 5/6; NFAT - Nuclear factor of activated T cells; PCP - Planar cell polarity; PLC - Phospholipase C; PP2A - Protein phosphatase 2 A; TCF/LEF - T-cell factor/lymphoid enhancer-binding factor; β-TrCP - β-transducin repeat-containing E3 ubiquitin-protein ligase)

Wnt signaling is a multifaceted pathway with canonical β-catenin-dependent and non-canonical β-catenin-independent branches. The canonical pathway involves Wnt ligands, Frizzled receptors, LRP5/6 co-receptors, Dishevelled, the β-catenin destruction complex, and TCF/LEF transcription factors, whereas non-canonical signaling acts through β-catenin-independent mediators such as PKC, CaMKII, NFAT, Rho, and Rac (Fig. 3A) [139]. Across cancers, Wnt-pathway dysregulation can occur through genetic, epigenetic, transcriptional, and miRNA-mediated mechanisms, but its biological effect depends on tumor type and molecular context (Supplementary Fig. 1 and Supplementary Table 1) [31, 140–149]. In meningioma, altered expression of Wnt-related components has been reported across different cohorts, but the evidence remains uneven and does not yet establish uniform activation of either pathway in grade 2 and grade 3 tumors [26, 42]. This distinction is important because canonical and non-canonical Wnt signaling may contribute differently to meningioma biology and therapeutic response.

### Canonical Wnt/β-catenin pathway

The canonical Wnt/β-catenin pathway regulates key cellular processes, including proliferation, differentiation, survival, and migration. In the absence of Wnt stimulation, the β-catenin destruction complex, composed principally of Axin, APC, GSK3β, and CK1α, phosphorylates β-catenin. Phosphorylated β-catenin is then recognized by β-TrCP within the SCF E3 ubiquitin ligase complex, leading to ubiquitination and proteasomal degradation. When Wnt ligands bind Frizzled receptors and LRP5/6 co-receptors, β-catenin degradation is inhibited. Stabilized β-catenin then accumulates, enters the nucleus, and regulates TCF/LEF target genes such as *MYC*, *CCND1*, and matrix-remodeling genes involved in tumor progression (Fig. 3A; Panel a and b) [150, 151]. Therapeutic targeting of this pathway is being explored in cancer, but pathway complexity and on-target toxicity remain important limitations [30, 152]. In meningioma, canonical Wnt signaling has been implicated mainly through altered expression of pathway components and downstream targets, rather than through a single recurrent Wnt-pathway mutation (Fig. 3B). Earlier microarray profiling across WHO grade 1, grade 2, and grade 3 meningiomas identified progression-associated expression differences involving Wnt-related genes such as *CTNNB1*,* CDK5R1*,* ENC1*, and *CCND1* [26]. Pećina-Šlaus et al. later summarized a broader set of canonical Wnt-related genes and proteins reported in meningioma, including *FZD2*,* FZD7*,* CSNK1A1*,* APC*,* AXIN1*,* CTNNB1*,* PPP2CA*,* TCF3*,* CCND1*,* ENC1*,* FRZB/SFRP3*,* SFRP1*,* CDH1*,* NDRG2*, and *CDK5R1* [42].

More recent meningioma-focused studies suggest that canonical Wnt activity may be relevant in selected aggressive contexts. A FOXM1/Wnt signaling axis has been linked to aggressive meningioma biology, mitotic gene programs, and poor clinical outcomes [46]. Cadherin-related regulation may also contribute to pathway activity, as *CDH2*/N-cadherin alterations and elevated protein expression correlate with total and active β-catenin, particularly in grade 2 and grade 3 meningiomas. *CDH1*/E-cadherin loss of heterozygosity also increases with grade and parallels higher active β-catenin [153]. Together, these studies suggest that canonical Wnt/β-catenin signaling contributes to meningioma biology in selected molecular contexts, but its grade-specific and therapeutic relevance still requires validation in larger, well-annotated cohorts.

### Non-canonical Wnt signaling pathways

Non-canonical Wnt signaling is β-catenin-independent and includes two major branches: Wnt/Ca^2+^ pathway and the Planar Cell Polarity (PCP) pathway.

#### Wnt/Ca^2+^ pathway

In the Wnt/Ca²⁺ pathway, ligands such as Wnt5a and Wnt11 bind Frizzled receptors and, through G-protein coupling and dishevelled activation, stimulate phospholipase C, leading to intracellular Ca²⁺ release and downstream activation of CaMKII, PKC, and calcineurin/NFAT signaling (Fig. 3A; Panel c) [154]. These pathways regulate cell polarity, migration, apoptosis, and other context-dependent cellular responses. In cancer, Wnt/Ca²⁺ signaling has been linked to migration, invasion, and epithelial–mesenchymal transition, but most of this evidence comes from non-meningioma tumor systems [155–157]. In meningioma, direct evidence for Wnt/Ca²⁺ signaling remains limited. Lei et al. showed that *NF2* regulates IP3R-mediated Ca²⁺ signaling and apoptosis in meningioma models, including the high-grade IOMM-Lee cell line [158]. A related study reported a positive association between *NF2* and *CAMK2A* expression in meningiomas and nervous-system tissues, suggesting a possible association between CAMK2A-related calcium signaling and NF2-associated meningioma biology [159]. Because CaMKII is also a downstream effector of non-canonical Wnt/Ca²⁺ signaling, these findings provide a plausible link between *NF2* loss, calcium signaling, and non-canonical Wnt output, but this connection has not yet been directly demonstrated in meningioma.

#### PCP pathway

The planar cell polarity pathway is a β-catenin-independent branch of Wnt signaling that regulates cytoskeletal organization, cell polarity, and directional movement (Fig. 3A; Panel d). In cancer, PCP-related signaling has been linked to tumor-cell motility and invasion [160–162], but direct evidence for a PCP-driven invasive program in meningioma remains limited. In meningioma, Merlin-deficient tumors show loss of contact inhibition with activation of Wnt/β-catenin signaling linked to PDGFR/Src and Rac/PAK pathways [64]. A *FAT2* (FAT atypical cadherin 2) mutation involving a PCP-related cadherin-family gene has been reported in a single spinal meningioma case [163], but direct evidence for a recurrent PCP-driven invasive program in meningioma remains limited. Intratumoral profiling has also identified regional differences in genes such as *CDH2* and *PTPRZ1*, supporting spatial heterogeneity in meningioma biology rather than direct proof of PCP activation [164]. Belumosudil (oral selective ROCK2 inhibitor) treatment induced oxidative phosphorylation and ROS-mediated cell death in IOMM-Lee high-grade meningioma cells, with pathway-level dysregulation including Wnt signaling [165], supporting further preclinical testing of ROCK-pathway targeting in aggressive meningioma models.

### Wnt signaling crosstalk and radioresistance in meningioma

**Fig. 4:**
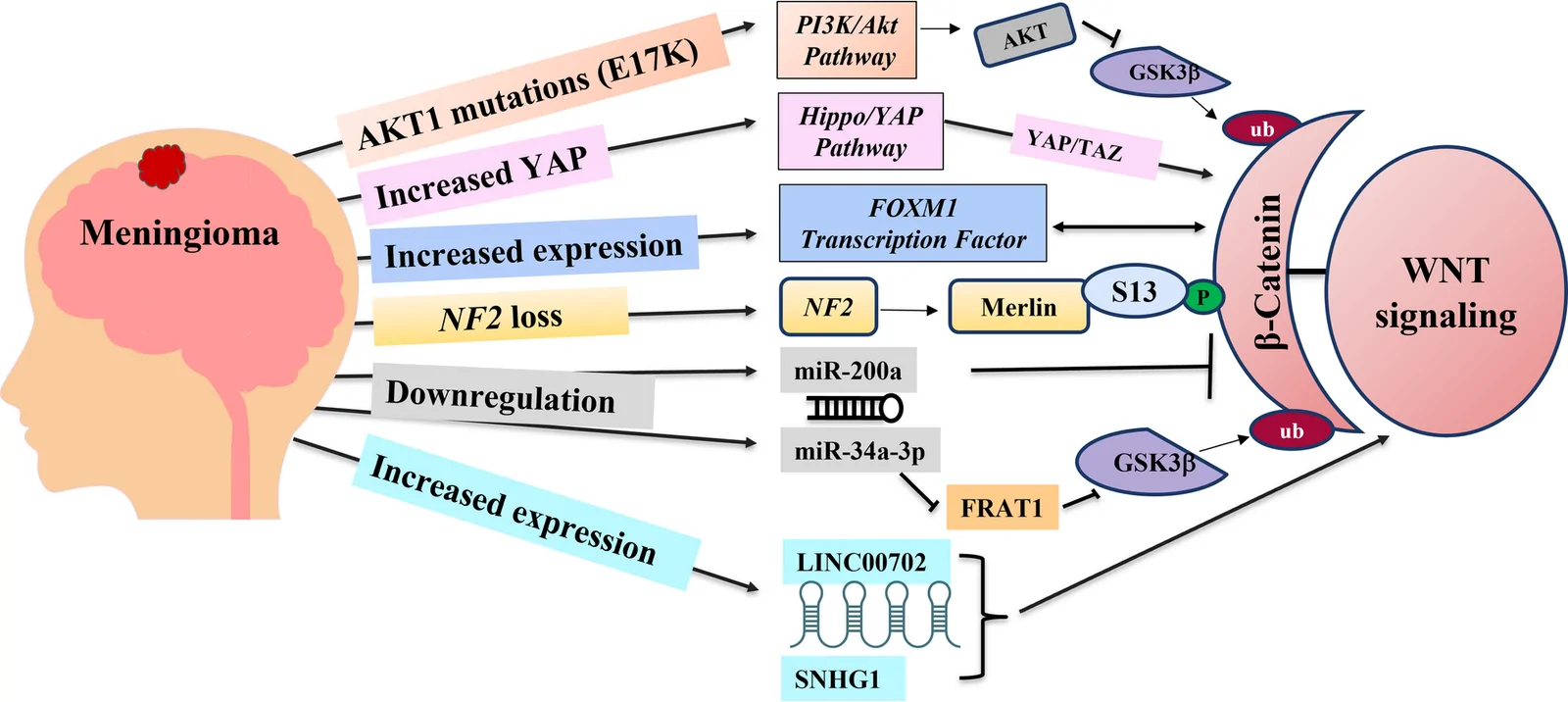
Wnt signaling pathway crosstalk with other proteins and signaling pathways in meningioma. (AKT - Protein kinase B; FOXM1 - Forkhead box M1; GSK3β - Glycogen synthase kinase 3 beta; LINC00702 and SNHG1 - long non-coding RNAs (lncRNAs); miR - microRNA; NF2 - Neurofibromatosis type 2 gene; PI3K - Phosphoinositide 3-kinase; YAP - Yes-associated protein; P - Phosphorylation; Ub - Ubiquitination)

Pathway crosstalk is relevant in meningioma because Wnt activity may be shaped by shared regulatory nodes that also influence tumor grade, proliferation, recurrence, and aggressive behavior. DVL1 is important in this context because dishevelled proteins can transmit both canonical β-catenin-dependent and non-canonical β-catenin-independent Wnt signals [166]. In meningioma, nuclear DVL1 has been associated with active β-catenin expression and higher tumor grade, supporting its role as a possible intracellular link between Wnt pathway activation and aggressive tumor features [167]. *NF2*/Merlin provides another important point of convergence. Merlin loss is associated with Hippo–YAP deregulation in meningioma [168], while Merlin S13 phosphorylation has been shown to regulate β-catenin interaction, Wnt pathway activity, and tumor growth [40]. Emerging abstract-level data further suggest that *NF2*-mutant meningiomas may shift WNT5A/B signaling toward a YAP/TAZ-associated response rather than a β-catenin-dominant output [169]. Wnt signaling is also linked to proliferative transcriptional programs in aggressive meningioma. Integrated molecular profiling identified a FOXM1/Wnt axis associated with mitotic gene expression, tumor proliferation, and poor clinical outcome [46]. In an immunohistochemical study across WHO grades, FOXM1 expression increased with tumor grade and was associated with recurrence probability and higher proliferation indices, while altered β-catenin expression was also linked to recurrence, supporting a clinically relevant FOXM1–β-catenin relationship in meningioma progression [170]. PI3K/Akt signaling may provide an additional secondary connection. *AKT1*^*E17K*^ mutations are mainly reported in non-*NF2*, often skull-base, lower-grade meningiomas [171], and *PIK3CA* mutations are enriched in non-*NF2* tumors, including primary benign WHO grade 1 meningiomas [172, 173]. These alterations are relevant to Wnt signaling because mechanistically, in other tumor systems, AKT can inhibit GSK3β through Ser9 phosphorylation, a process that may stabilize β-catenin and enhance β-catenin-dependent transcription [174] (Fig. 4). Reduced p53 expression has also been inversely associated with β-catenin expression in meningioma, suggesting a possible but still correlative p53–β-catenin relationship [175].

These crosstalks may also be relevant to treatment resistance, particularly radiotherapy response in recurrent higher-grade meningioma. In recurrent WHO grade 2/3 meningiomas, postoperative radiotherapy was associated with reduced recurrence risk in *NF2*-wild-type tumors, whereas *NF2*-mutant tumors showed no clear benefit and a possible trend toward earlier recurrence after radiotherapy [51]. Experimental work in grade 2 meningioma further showed that *NF2* loss alone had only a modest effect on radiation response, but *NF2* knockdown under hypoxic conditions increased clonogenic survival after irradiation [52]. In the same study, spontaneous necrosis together with *NF2* mutation was associated with worse post-radiation local control, and DKK2 (context-dependent Wnt/β-catenin regulator) was included among the shared upregulated genes in radiation-resistant in vitro and patient-derived tumor comparisons [52]. These findings do not prove that Wnt signaling drives *NF2-*associated radioresistance, but they place Wnt-related signaling within a broader *NF2*–hypoxia–radiation response network. This link is biologically plausible because phospho Merlin S13 regulates Merlin–β-catenin interaction and β-catenin-dependent Wnt activity [40] as discussed earlier. More direct support for a β-catenin-associated radiosensitization strategy comes from an in vitro study in which HDAC6 inhibition with CAY10603 enhanced the cytotoxic effect of radiotherapy in immortalized and patient-derived meningioma models, increased radiation-induced DNA damage, reduced nuclear accumulation of β-catenin and MCM2, and suppressed c-MYC expression [176]. Overall, current evidence supports a plausible connection between *NF2* dysfunction, hypoxia, β-catenin/Wnt activity, and radiotherapy tolerance, but direct proof that Wnt/β-catenin signaling is an established driver of radioresistance in meningioma remains limited.

## Dysregulation of Wnt signaling in higher-grade (grade 2 and 3) meningiomas

While Wnt/β-catenin signaling is implicated in the tumorigenesis and progression of high-grade meningiomas, its role is complex and not entirely understood. Recent studies have highlighted the involvement of this pathway in various aspects of meningioma biology, including cell proliferation, invasiveness, and resistance to apoptosis. However, the precise mechanisms and clinical significance remain areas of active research. This section summarizes evidence of Wnt pathway dysregulation, clinical correlations with tumor aggressiveness, and epigenetic modulation.

### Aberrant β-Catenin expression and clinical implications

β-catenin expression in meningioma is difficult to interpret as a single biomarker because different studies have examined different cellular pools of the protein. Membranous β-catenin largely reflects cadherin–catenin cell–cell adhesion, whereas nuclear or active/non-phosphorylated β-catenin is more directly related to canonical Wnt pathway activity. This distinction helps explain why some cohorts link reduced β-catenin with invasion and recurrence, while others report nuclear or active β-catenin in higher-grade tumors. In a 107-case immunohistochemical series, E-cadherin, α-catenin, and β-catenin were broadly expressed at the membrane and/or cytoplasm across meningioma variants and WHO grades, with no consistent association with malignancy or Ki-67 proliferation index [177]. In contrast, reduced E-cadherin and β-catenin expression has been linked to invasive meningiomas and invasive tumor regions, supporting a relationship between adhesion-complex loss and local invasion [178]. This adhesion-based interpretation is strengthened by evidence that membranous E-cadherin is absent in a subset of meningiomas and membranous β-catenin is frequently lost, with β-catenin accumulating mainly in a perinuclear Golgi/ERGIC (Endoplasmic Reticulum–Golgi Intermediate Compartment) rather than in the nucleus, independent of *NF2* status or WHO grade [179]. Reduced E-cadherin and β-catenin expression has also been associated with higher WHO grade, invasion, peritumoral edema, and postoperative recurrence, again suggesting adhesion remodeling rather than direct proof of canonical Wnt activation [180].

Evidence for canonical Wnt pathway involvement comes from a more specific set of observations. *APC* loss of heterozygosity has been associated with abnormal APC protein expression and nuclear β-catenin localization in meningioma, supporting APC-related Wnt dysregulation in a subset of tumors [140]. In one cohort, nuclear β-catenin and N-cadherin expression were reported more strongly in high-grade meningiomas and correlated with more severe peritumoral brain edema [181]. Wnt-receptor expression may add another layer of heterogeneity, as FZD9 showed differential expression in grade 2–3 compared with grade 1 meningiomas in a stem-cell-marker analysis [182]. However, β-catenin is not consistently increased in aggressive tumors. In one tissue microarray cohort of 153 meningiomas, high β-catenin expression was associated with lower tumor grade, lower recurrence rate, and lower incidence of brain invasion. In contrast, high galectin-3 expression was associated with higher grade, recurrence, and brain invasion. Nevertheless, in multivariate logistic regression, β-catenin and galectin-3 expression levels were significant predictors of recurrence, suggesting that β-catenin retained recurrence-related prognostic relevance despite its inverse univariate association with tumor grade [183]. Similarly, in an immunohistochemical study across WHO grades, FOXM1 expression increased with tumor grade and was associated with recurrence probability and higher proliferation indices. In contrast, β-catenin expression was lower in WHO grade 2/3 tumors and was also associated with recurrence, supporting an altered FOXM1/β-catenin marker pattern in meningioma progression rather than direct proof of canonical Wnt activation [170]. Separately, nuclear DVL1 expression was significantly associated with active β-catenin expression and higher meningioma grade, supporting DVL1-linked Wnt activation in a subset of tumors [167].

**Fig. 5:**
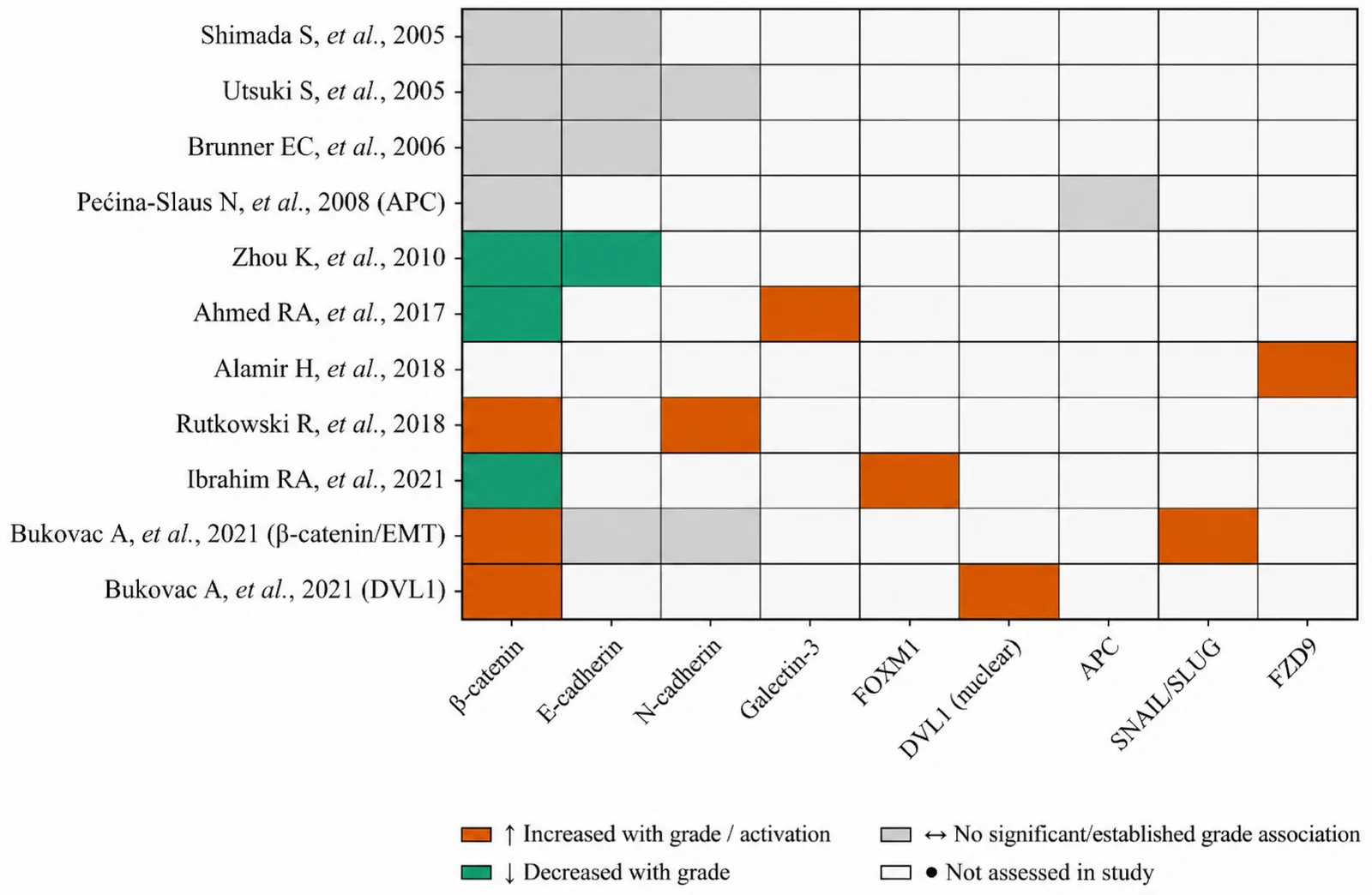
Overview of representative studies reporting Wnt/β-catenin pathway readouts, EMT/cadherin switching, and related markers across WHO grades 1–3 meningiomas. (APC - Adenomatous polyposis coli; DVL1 - Dishevelled 1; EMT - Epithelial–mesenchymal transition; FZD9 - Frizzled class receptor 9)

Taken together, β-catenin should not be presented as a uniform prognostic marker in meningioma. Differences in antibody specificity, staining compartment, scoring method, cohort composition, and clinical endpoints likely account for much of the inconsistency across published studies. Prognostic statements about β-catenin in meningioma should therefore be compartment-specific and cohort-qualified (Fig. 5, Supplementary Table 2).

### Epigenetic and non-coding RNA regulation of Wnt signaling in meningioma

Epigenetic and post-transcriptional regulation may contribute to Wnt pathway dysregulation in meningioma, but the strongest evidence is concentrated around selected Wnt antagonists and RNA-regulatory networks rather than a broad pathway-wide silencing program. The clearest DNA-methylation evidence involves *SFRP1*, a secreted Wnt antagonist. *SFRP1* promoter hypermethylation has been linked to poorer local recurrence-free survival in aggressive meningioma within a broader FOXM1/Wnt signaling axis [46]. Earlier gene-expression analysis of progressive and recurrent meningiomas also identified reduced expression of Wnt-related genes, including *SFRP1* and *SFRP4*, with *SFRP1* promoter hypermethylation proposed as one mechanism of gene repression [184]. Reduced SFRP1 expression has also been reported in recurrent and atypical meningiomas, supporting loss of Wnt-inhibitory control in a subset of tumors [97]. However, *SFRP1* methylation does not appear to have uniform prognostic value across all clinical settings, as *SFRP1* promoter hypermethylation was not associated with regrowth-free survival in WHO grade 1 meningiomas treated with Gamma Knife radiosurgery [185].

Genome-wide methylation studies provide additional pathway level support. In a matched case control study of WHO grade 2 meningiomas, tumors with recurrent or clinically dismal behavior showed broad CpG methylation differences enriched in Wnt and cadherin signaling pathways, rather than a single promoter-specific methylation event. These changes involved several Wnt- and adhesion-related genes, including *CDH2*,* CDH13*,* CTNNA2*,* WNT16*,* SFRP1*,* ANKRD6*,* BMPR1A*, and multiple protocadherin family members, and were distributed across CpG islands, shores, shelves, gene bodies, intergenic regions, and transcription start sites [186]. Histone- and polycomb-related regulation may also intersect with Wnt biology in selected meningioma subtypes. *BAP1*-altered meningiomas represent a distinct aggressive molecular subtype with deregulation of Polycomb repressive complex target genes. RNA-seq analysis reported increased expression of WNT signaling pathway members, including *WNT3A*,* WNT10A*,* WNT10B*,* WNT11*, and *WNT16*, in *BAP1*-altered meningiomas, with several of these pathway members also elevated in H3K27me3-loss meningiomas [187]. This supports a possible link between altered chromatin regulation and WNT-ligand expression, although functional validation is still needed.

Non-coding RNAs provide some of the strongest direct evidence for Wnt regulation in meningioma. miR-200a is reduced in meningioma, and its downregulation increases β-catenin and cyclin D1 expression, linking miR-200a loss to Wnt/β-catenin-associated proliferation [95]. miR-34a-3p is also relevant because it directly targets FRAT1, a Wnt/β-catenin pathway component as well as SMAD4 and BCL2 and alters proliferation and apoptosis in meningioma cells [188]. Long non-coding RNAs further support this regulatory layer. LINC00702 promotes malignant meningioma progression through the miR-4652-3p/ZEB1 axis and activation of Wnt/β-catenin signaling [189]. Similarly, the SNHG1/miR-556-5p/TCF12 feedback loop enhances meningioma tumorigenesis through Wnt signaling [190]. Together, these findings support a mechanism-specific role for epigenetic and RNA-mediated regulation of Wnt signaling in meningioma, with the clearest support from *SFRP1* methylation/repression, Wnt/cadherin pathway-level methylation differences, polycomb repressive complex-associated WNT-ligand upregulation, miR-200a loss, miR-34a-3p/FRAT1 regulation, and Wnt-activating lncRNA networks (Fig. 4).

## Therapeutic targeting of Wnt signaling in grade 2 and 3 meningiomas

Therapeutic targeting of Wnt-associated signaling in meningioma remains preclinical, with the strongest evidence coming from studies that directly perturb β-catenin-dependent signaling or related upstream regulators. In the Merlin S13 phosphorylation study, β-catenin suppression reduced Wnt signaling, impaired meningioma cell proliferation and xenograft growth, and prolonged survival in preclinical models [40]. The most direct meningioma-specific pharmacologic evidence comes from ICG-001, a CBP/β-catenin inhibitor tested in *NF2*-mutant or Merlin-low meningioma models. In functional models, ICG-001 reduced proliferation, induced G1 arrest, increased p21, and decreased survivin in meningioma cell models. It suppressed growth of Merlin-negative/*NF2*-associated patient-derived subcutaneous flank xenograft models in female BALB/c nude mice [39]. The same study reported attenuation of FOXM1-mediated Wnt/β-catenin signaling after ICG-001 treatment, making it the clearest meningioma-specific preclinical example of pharmacologic Wnt/β-catenin pathway targeting [39].

Additional therapeutic evidence is supportive but less direct. HDAC6 inhibition with CAY10603 enhanced the cytotoxic effect of radiotherapy in immortalized and patient-derived meningioma models, increased radiation-induced DNA damage, reduced nuclear β-catenin and MCM2 accumulation, and suppressed c-MYC expression [176]. This suggests a β-catenin-associated radiosensitization effect, but HDAC6 inhibition should not be described as direct Wnt blockade. Earlier in vitro work using plant-derived compounds from citrus, ginger, and mushroom sources reported antitumor activity in human meningioma cells through suppression of Wnt/β-catenin signaling [191]. Recent evidence further suggests that *NF2*-mutant meningioma models may be sensitive to inhibition of an alternative WNT5A/B–YAP/TAZ pathway, but this remains preliminary until full peer-reviewed validation is available [169].

**Fig. 6:**
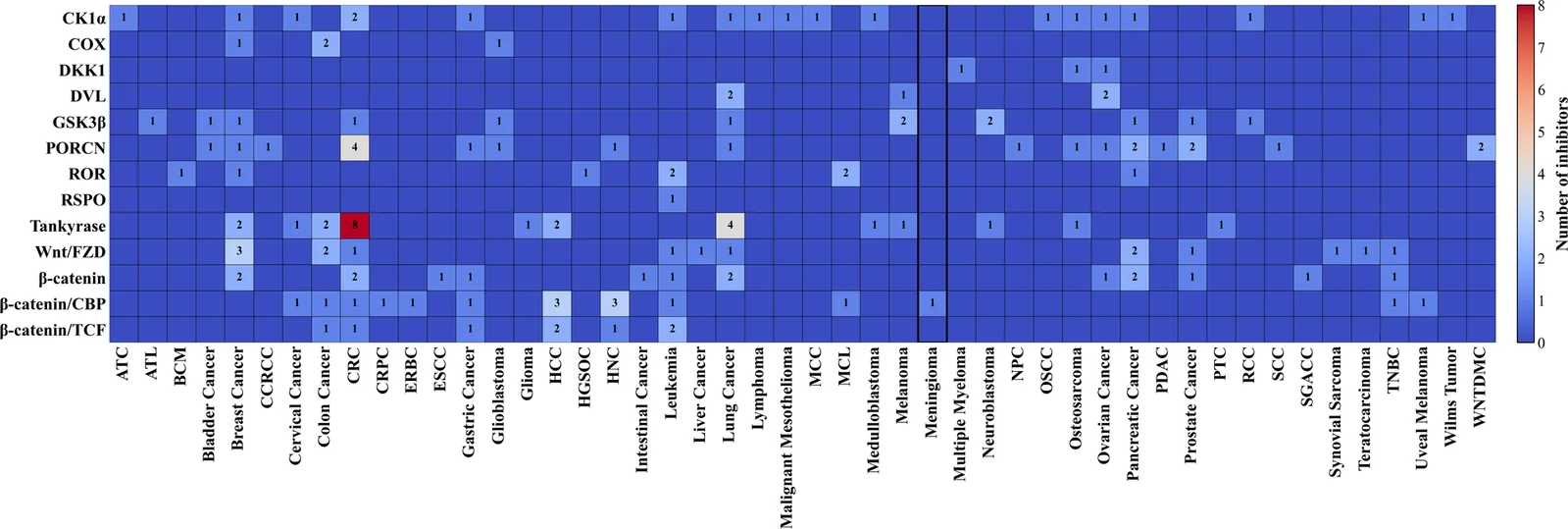
Preclinical Wnt-pathway–targeting agents and modulators across cancer types. Agents included in the heatmap were grouped according to their reported target or mechanistic class: RSPO-targeting agent (OMP-131R10 [rosmantuzumab]); DKK1-targeting agents (BHQ880, DKN-01); tankyrase/destruction-complex inhibitors (XAV939, IWR-1, NVP-TNKS656, LZZ-02, E7449, G007-LK, JW55, AZ1366, MSC2504877, JW67, JW74); GSK3β inhibitors/modulators (9-ING-41, LY2090314); CK1α-targeting agents/modulators (pyrvinium pamoate, SSTC3); Wnt/FZD- or WNT5A-axis–targeting agents/modulators (OMP-54F28 [ipafricept], OMP-18R5 [vantictumab], Foxy-5, OTSA-101, Fz7-21, IC-2); ROR-targeting agents and modalities (cirmtuzumab, anti-ROR1 CAR T-cell therapy, NBE-002, VLS-101 [zilovertamab vedotin], KAN0439834); DVL inhibitors/modulators (FJ9, 3289-8625, sulindac); PORCN inhibitors (WNT974 [LGK974], Wnt-C59, CGX1321, IWP-2, ETC-159, RXC004, GNF-6231); β-catenin pathway inhibitors/modulators (CWP232291, KYA1797K, ST316, FH535); β-catenin/CBP-associated inhibitors/modulators (PRI-724, ICG-001, E7386, isoquercitrin); β-catenin/TCF inhibitors (PKF115-584, CGP049090, iCRT3, LF3); and COX-associated Wnt modulators (aspirin, celecoxib). The number shown in each heatmap cell indicates the number of distinct agents included for the corresponding agent class and cancer type.

To our knowledge, Wnt-pathway inhibition has not yet been evaluated in a dedicated meningioma clinical trial, highlighting a translational gap between emerging pathway-level evidence and therapeutic development. This is important because most Wnt-targeting approaches, including PORCN inhibitors, Frizzled/receptor-targeting agents, tankyrase inhibitors, β-catenin–CBP or β-catenin–TCF transcriptional inhibitors, monoclonal antibodies, and gene-based approaches, have largely been developed in other cancer settings (Figure 6 and Supplementary Table 3) [31, 192–195]. Despite progress in drug development, Wnt-targeted therapies have not become routine cancer treatments. One reason is that effective pathway inhibition must be balanced against the physiological role of Wnt/β-catenin signaling in adult tissue maintenance, regeneration, and stem-cell homeostasis. This creates therapeutic-index and toxicity concerns for systemic therapy [195]. For meningioma, the most rational next step is biomarker-guided preclinical testing rather than broad, unselected Wnt inhibition. This is particularly important because high-grade and recurrent meningiomas show spatial and molecular heterogeneity [138, 164]. Wnt-pathway modulation should therefore be tested both alone and in rational combinations.

## Challenges and considerations in targeting the Wnt pathway

Targeting Wnt signaling in meningioma is biologically attractive, but it remains an early translational concept rather than a validated therapeutic strategy. Several meningioma-specific studies support a link between Wnt-associated activity and aggressive tumor biology, including FOXM1-linked proliferative programs, DVL1-associated active β-catenin signaling, and Phospho Merlin (S13)–regulated β-catenin/Wnt activity in experimental models [40, 46, 167]. In *NF2*-mutant meningioma models, the CBP/β-catenin inhibitor ICG-001 suppressed Wnt/β-catenin–associated tumor growth, providing preclinical support for Wnt inhibition in selected molecular contexts [39]. The central translational challenge is therefore not simply whether Wnt can be inhibited, but whether Wnt-active tumors can be identified, pathway suppression can be confirmed after treatment, and adequate intratumoral drug exposure can be achieved without unacceptable toxicity.

### Pathway complexity and therapeutic window

One difficulty is that Wnt signaling is not a single linear pathway. It includes multiple Wnt ligands, Frizzled receptors, LRP5/6 co-receptors, Dishevelled proteins, destruction-complex components, transcriptional co-regulators, and both β-catenin-dependent and β-catenin-independent branches [196]. Blocking one ligand, receptor, or intracellular node may leave other pathway inputs active. The therapeutic window is also narrow because Wnt signaling contributes to normal tissue homeostasis, regeneration, and stem-cell regulation, particularly in tissues such as the intestinal epithelium and hematopoietic system [196, 197]. For meningioma, any systemic Wnt-directed approach would need to show a meaningful antitumor effect while limiting damage to normal stem-cell compartments and tissue-repair programs.

### Redundancy and feedback loops

Pathway redundancy and feedback regulation add another layer of complexity. A tumor with several active Wnt inputs may retain downstream signaling even after one component is inhibited. AXIN2 illustrates this problem well: it is induced by Wnt/β-catenin signaling, but it also functions as a negative-feedback regulator that restrains pathway activity after activation [198]. A single baseline marker is therefore unlikely to capture Wnt activity reliably in meningioma. Preclinical studies should combine baseline and post-treatment readouts, including AXIN2, LEF1, β-catenin localization, and active β-catenin, together with proliferation-linked markers such as FOXM1 and cyclin D1.

### Drug delivery and tumor exposure

Drug delivery requires careful interpretation in meningioma. Many meningiomas are extra-axial, dural-based, and highly vascular tumors, so delivery cannot be framed only as a classical blood-brain barrier problem. Meningioma vessels often lack typical BBB properties, which contributes to contrast enhancement on imaging [199]. However, intratumoral exposure may still vary with tumor location, skull-base anatomy, vascular supply, prior surgery or radiotherapy, brain invasion, efflux mechanisms, stromal composition, and drug formulation. Wnt/β-catenin signaling also contributes to endothelial barrier properties in experimental BBB models, and endothelial Wnt-targeting strategies have been explored to transiently open the blood-tumor barrier in brain metastasis models [200, 201]. These studies provide useful proof-of-principle, but they remain indirect evidence for meningioma. For Wnt-directed therapy in this disease, the practical question is whether the drug reaches the tumor, suppresses the pathway in situ, and maintains an acceptable safety profile.

### Biomarker selection

Biomarker selection will be essential because Wnt activity is unlikely to be uniform across meningiomas. Candidate markers include nuclear or active β-catenin, nuclear DVL1 with active β-catenin, *SFRP1* silencing or promoter hypermethylation, *NF2*/Merlin status, Merlin S13 phosphorylation status, and FOXM1/Wnt-high proliferative programs [39, 40, 46, 153, 167, 184]. These markers capture different aspects of meningioma biology and should not be interpreted as equivalent indicators of Wnt pathway activation. Reduced membranous β-catenin may indicate disruption of the cadherin-catenin adhesion complex rather than direct activation of canonical Wnt transcription, whereas nuclear or active β-catenin is more closely linked to β-catenin-dependent Wnt signaling [153, 178, 179]. Similarly, *NF2* mutation, Merlin loss, and Merlin S13 phosphorylation represent related but distinct biological contexts [39, 40]. A practical translational panel would therefore need to combine Wnt activity markers, including AXIN2, LEF1, β-catenin localization, and active β-catenin, with proliferation or cell-cycle markers such as FOXM1 and cyclin D1. These findings should be interpreted together with *NF2* status, Merlin expression or phosphorylation state, *SFRP1* methylation or expression, tumor grade, recurrence status, and prior treatment history.

### Delivery strategies

Improved delivery strategies may support future Wnt-directed studies, but none is currently validated for meningioma. Convection-enhanced delivery, prodrug design, nanoparticle systems, and receptor-mediated transcytosis approaches are being explored to improve CNS or tumor drug exposure [201–205]. In meningioma, these approaches remain investigational and their value will depend on whether they improve tumor exposure without compromising pathway selectivity or increasing toxicity in anatomically diverse tumors.

### Future directions and combination strategies

Future work should move away from broad, unselected Wnt inhibition and toward biomarker-defined testing. In meningioma models, Wnt-pathway modulation could be evaluated with radiotherapy, PI3K-AKT-mTOR inhibition, FAK/Hippo-YAP-directed therapy, CDK-pathway inhibition, anti-angiogenic therapy, or immune-based approaches. At present, these combinations are best framed as translational hypotheses. Indirect strategies may also be relevant, particularly those targeting upstream *NF2*/Merlin-related signaling or FOXM1-associated transcriptional programs [39, 40, 46]. These questions should be tested in molecularly annotated grade 2 and grade 3 patient-derived cultures, organoids, and xenografts, with parallel measurement of antitumor effect, Wnt pharmacodynamic markers, intratumoral drug exposure, and toxicity. Patient-derived meningioma organoids offer a useful platform for studying tumor biology and therapeutic response, although their role in Wnt-specific pharmacology and grade-specific prediction still requires validation [206, 207]. Overall, Wnt-directed therapy in meningioma remains preclinical, but it is a rational direction if developed with tumor-specific biomarkers, realistic delivery studies, and careful attention to the molecular and anatomical heterogeneity of high-grade and recurrent disease.

## Conclusion

High-grade meningiomas, including WHO grade 2 and grade 3 tumors, remain difficult to manage because of their biological heterogeneity, aggressive clinical behavior, high recurrence rates, and limited treatment options beyond surgery and radiotherapy. The evidence reviewed here supports an important role for Wnt-associated signaling in meningioma biology, particularly in relation to tumor proliferation, invasive behavior, and possibly treatment resistance in selected molecular contexts.

Canonical Wnt/β-catenin signaling appears to be altered in subsets of grade 2 and grade 3 meningiomas, where it may contribute to malignant behavior through transcriptional programs linked to cell-cycle progression and tumor growth. Non-canonical Wnt signaling is less well studied in meningioma, but its known roles in cell polarity, migration, and microenvironmental communication make it a relevant area for further investigation, especially in the context of invasion and tumor progression. Wnt pathway dysregulation in meningioma is unlikely to arise from a single mechanism. Instead, available studies point to several possible layers of regulation, including *NF2*/Merlin-related signaling, epigenetic silencing of Wnt antagonists, altered β-catenin localization, DVL1-associated pathway activity, and microRNA-mediated control. Importantly, β-catenin findings need careful interpretation and the variable association of β-catenin localization with tumor grade, recurrence, and prognosis further emphasizes the need to define its biomarker value more precisely.

Translating these observations into therapy will require a more integrated view of Wnt signaling in meningioma. Large-scale studies combining genomics, epigenomics, transcriptomics, proteomics, and spatial profiling could help define which tumors are truly Wnt-active and how this state relates to grade, recurrence, *NF2*/Merlin status, methylation class, and prior treatment. Clinically useful biomarkers may include β-catenin localization, active β-catenin, *AXIN2*,* LEF1*,* DVL1*, *SFRP1* methylation or expression, Merlin phosphorylation status, and FOXM1-linked proliferative programs. A combined marker panel is likely to be more informative than any single readout, particularly for distinguishing true pathway activation from adhesion remodeling or nonspecific proliferation-associated changes.

At present, Wnt-directed therapy in meningioma remains a preliminary experimental concept, supported by limited meningioma-specific evidence and no dedicated clinical trials of Wnt-pathway inhibitors. Before clinical trials, Wnt-directed agents should be tested in molecularly annotated grade 2 and grade 3 patient-derived cultures, organoids, and xenograft models, with parallel assessment of pathway suppression, tumor response, intratumoral drug exposure, and toxicity. Combination strategies may be particularly relevant, including approaches that pair Wnt modulation with radiotherapy, PI3K-AKT-mTOR inhibition, FAK/Hippo-YAP-directed therapy, CDK-pathway inhibition, anti-angiogenic therapy, or immune-based treatment. These combinations should first be evaluated in meningioma-specific models rather than extrapolated from other cancers.

Given the rarity of recurrent high-grade meningiomas, especially WHO grade 3 disease, future Wnt-directed studies will need multi-institutional collaboration and biomarker-enriched designs. Treatment should be tested in tumors with clear evidence of Wnt pathway activation, together with pharmacodynamic evidence that the pathway is being inhibited. Clinical endpoints should also go beyond radiographic response and include progression-free survival, neurological function, steroid requirement, quality of life, and treatment-related toxicity. In this context, Wnt signaling should be viewed not as a universal target in meningioma, but as a biologically selected therapeutic opportunity whose value will depend on accurate patient selection and meaningful clinical benefit.

## Supplementary Information

Below is the link to the electronic supplementary material.

Supplementary Material 1.

Supplementary Material 2.

Supplementary Material 3.

Supplementary Material 4.

## Data Availability

The datasets used and/or analysed during the current study are available from the corresponding author on reasonable request.

## Abbreviations

ACC: Adrenocortical carcinoma
AKT: Protein kinase B
AML: Acute myeloid leukemia
APC: Adenomatous polyposis coli
ATC: Anaplastic thyroid cancer
ATL: Adult T-cell leukemia
BAF: BRG1/BRM-associated factor
BBB: Blood–brain barrier
BCM: B-cell malignancies
BCR: Breakpoint cluster region
β-TrCP: β-transducin repeat-containing E3 ubiquitin-protein ligase
BTB: Blood–tumor barrier
CaMKII: Calcium/calmodulin-dependent protein kinase II
CBP: CREB-binding protein
CDK: Cyclin-dependent kinase
CDKN2A/B: Cyclin-dependent kinase inhibitor 2 A/B
CK1α: Casein kinase 1 alpha
CLL: Chronic lymphocytic leukemia
CNA: Copy-number alteration
CNS: Central nervous system
CNS5: Fifth edition of the WHO Classification of Central Nervous System Tumours
CNV: Copy-number variation
COX: Cyclooxygenase
CRC: Colorectal cancer
CRPC: Castration-resistant prostate cancer
CSC: Cancer stem cell
DKK1: Dickkopf WNT signaling pathway inhibitor 1
DMD: Duchenne muscular dystrophy gene/dystrophin
DVL: Dishevelled
EGFR: Epidermal growth factor receptor
EMT: Epithelial–mesenchymal transition
ERBC: Endocrine-resistant breast cancer
ERGIC: Endoplasmic reticulum–Golgi intermediate compartment
ESCC: Esophageal squamous cell carcinoma
FAK: Focal adhesion kinase
FZD: Frizzled receptor
GBM: Glioblastoma
GFAP: Glial fibrillary acidic protein
GSK3β: Glycogen synthase kinase 3 beta
H3Ac: Histone H3 acetylation
H3K4me3: Trimethylation of histone H3 lysine 4
H3K9Ac: Acetylation of histone H3 lysine 9
H3K9me3: Trimethylation of histone H3 lysine 9
H3K27me3: Trimethylation of histone H3 lysine 27
HCC: Hepatocellular carcinoma
HDAC: Histone deacetylase
HDAC6: Histone deacetylase 6
HGSOC: High-grade serous ovarian cancer
HNC: Head and neck cancer
HNSCC: Head and neck squamous cell carcinoma
HPF: High-power field
IF: Immunofluorescence
IHC: Immunohistochemistry
IP3R: Inositol 1,4,5-trisphosphate receptor
IRS: Immunoreactive score
JNK: c-Jun N-terminal kinase
LOH: Loss of heterozygosity
LRP: Low-density lipoprotein receptor-related protein
LRP5/6: Low-density lipoprotein receptor-related protein 5/6
MAPK: Mitogen-activated protein kinase
MCC: Merkel cell carcinoma
MCL: Mantle cell lymphoma
MeSH: Medical Subject Headings
miR: microRNA
MSI: Microsatellite instability
mTOR: Mechanistic target of rapamycin
ncBAF: Non-canonical BAF
NF2: Neurofibromatosis type 2 gene
NFAT: Nuclear factor of activated T cells
NGS: Next-generation sequencing
NPC: Nasopharyngeal carcinoma
NSAID: Non-steroidal anti-inflammatory drug
NSCLC: Non-small cell lung cancer
OLIG2: Oligodendrocyte transcription factor 2
OS: Overall survival
OSCC: Oral squamous cell carcinoma
PAK: p21-activated kinase
PBAF: Polybromo-associated BAF
PCP: Planar cell polarity
PDAC: Pancreatic ductal adenocarcinoma
PDGFR: Platelet-derived growth factor receptor
PDX: Patient-derived xenograft
PFS: Progression-free survival
PFS-6: Progression-free survival at 6 months
PI3K: Phosphoinositide 3-kinase
PLC: Phospholipase C
PORCN: Porcupine O-acyltransferase
PP2A: Protein phosphatase 2 A
PRC2: Polycomb repressive complex 2
PTC: Papillary thyroid carcinoma
PTBE: Peritumoral brain edema
RB: Retinoblastoma protein
RCC: Renal cell carcinoma
ccRCC: Clear-cell renal cell carcinoma
ROR: Receptor tyrosine kinase-like orphan receptor
RSPO: R-spondin
SCC: Squamous cell carcinoma
sFRP/SFRP: Secreted Frizzled-related protein
SGACC: Salivary gland adenoid cystic carcinoma
SHH: Sonic hedgehog
SOX2: SRY-box transcription factor 2
SRC: SRC proto-oncogene/non-receptor tyrosine kinase
SSEA4: Stage-specific embryonic antigen 4
SUFU: Suppressor of fused
SWI/SNF: Switch/sucrose non-fermentable
TAZ: Transcriptional coactivator with PDZ-binding motif
TCF/LEF: T-cell factor/lymphoid enhancer-binding factor
TERT: Telomerase reverse transcriptase
TGF-β: Transforming growth factor beta
TNBC: Triple-negative breast cancer
TNKS: Tankyrase
T-ALL: T-cell acute lymphoblastic leukemia
VEGF: Vascular endothelial growth factor
WIF1: Wnt inhibitory factor 1
WntDMC: Wnt-driven mammary cancer
YAP: Yes-associated protein
