# Supplementary material for: The Wnt signaling networks in higher-grade meningiomas: from molecular pathogenesis to therapeutic innovation
Source: Cancer Cell Int. 2026 Jul 24;26:345. doi: 10.1186/s12935-026-04393-1 (PMC13685165; doi:10.1186/s12935-026-04393-1)
Supplement: Supplementary file 1 [file 12935_2026_4393_MOESM1_ESM.docx]

**Supplementary Table 2**. Evidence for Wnt/β-catenin and cadherin–catenin pathway alterations in meningioma. This table summarizes human meningioma cohort studies evaluating Wnt-related pathway components, including β-catenin, E-cadherin, N-cadherin, APC, FZD9, FOXM1, DVL1, and EMT-associated markers. For each study, the table lists cohort size, WHO grade composition, methods used, and principal grade-wise findings.

| **Study type** | **Cohort size** | **Grades in cohort** | **Methods used** | **Principal findings (grade-wise summary)** | **Reference** |
| --- | --- | --- | --- | --- | --- |
| Human tumor cohort | 107 meningiomas (plus 15 hemangioblastomas, 4 hemangiopericytomas as comparators) | grade 1 = 84, grade 2 = 18, grade 3 = 5 | IHC for E-cadherin and α-, β-, γ-catenin with semi-quantitative immunostaining scores; Ki-67 labeling index; statistical correlation with WHO grade, histological subtype, and Ki-67 | E-cadherin and α-/β-catenin were expressed in most meningiomas, while adhesion-molecule expression was detected in all grade 2–3 cases. Immunostaining scores showed no significant association with WHO grade or Ki-67 index. γ-catenin was also present but weaker/less frequent in fibrous subtype. Thus, cadherin–catenin expression is ubiquitous and not associated with malignancy grade, but differs from hemangioblastoma/hemangiopericytoma and may aid differential diagnosis | [1] |
| Human tumor cohort | 103 meningiomas | WHO grade 1 = 98 (61 meningothelial, 25 fibrous, 12 invasive), WHO grade 3 = 5 (anaplastic) | Immunohistochemistry for E-cadherin, N-cadherin, β-catenin and Ki-67 on formalin-fixed, paraffin-embedded sections; semi-quantitative 4-grade scoring of E-cadherin immunoreactivity (high >80%, intermediate 20–80%, low <20%, negative); statistical analysis with Kruskal–Wallis, Fisher’s exact test, and t-test. | None of the 103 meningiomas expressed N-cadherin. E-cadherin/β-catenin positivity at cell–cell borders was seen in 10/12 (83%) invasive, 61/61 (100%) meningothelial, 0/25 (0%) fibrous, and 3/5 (60%) anaplastic meningiomas. E-cadherin expression level was predominantly high/intermediate in meningothelial (42/61, 69%), completely negative in fibrous (25/25, 100%), and intermediate in invasive and anaplastic subtypes (invasive: 2/12 high, 4/12 intermediate, 4/12 low, 2/12 negative; anaplastic: 0/5 high, 1/5 intermediate, 2/5 low, 2/5 negative). In invasive meningiomas, the expansive (brain-infiltrating) part showed reduced E-cadherin/β-catenin immunoreactivity compared with the central/whole tumor. Ki-67 labeling index was 0.7 ± 0.4% in meningothelial, 0.5 ± 0.3% in fibrous, 1.5 ± 1.0% in invasive, and 15.6 ± 12.6% in anaplastic meningiomas, with significantly higher Ki-67 in invasive than in meningothelial/fibrous tumors (p<0.05). | [2] |
| Human tumor cohort + primary cell cultures | 38 meningiomas + 8 primary meningioma cell cultures; 2 colorectal adenocarcinoma brain metastases and non-tumorous arachnoid as controls | grade 1 = 18, grade 2 = 16, grade 3 = 4 | IHC for β-catenin and E-cadherin on 38 tumors; Western blot on tumor lysates and fractionated primary cultures; subcellular fractionation to ER/Golgi/ERGIC; immunofluorescence co-localization with Golgi marker; karyotyping for chromosome 22/NF2 status | Membranous β-catenin was present in only 8/38 meningiomas (21%; 2/18 grade 1, 5/16 grade 2, 1/4 grade 3), whereas most tumors showed granular perinuclear/cytoplasmic β-catenin localized to ER/Golgi fractions and no nuclear staining. E-cadherin was detected at the cell membrane in 25/38 (66%) tumors (7/38 negative; 6/38 only cytoplasmic). Only 7/38 (18%) meningiomas—2 grade 1, 4 grade 2, 1 grade 3—showed concomitant membranous β-catenin and E-cadherin, and this altered cadherin–catenin distribution was independent of WHO grade, histological subtype, brain invasion, and chromosome 22/NF2 loss. | [3] |
| Human tumor cohort | 33 meningiomas (32 informative/heterozygous for APC markers) | Grade 1 =29  (14 meningothelial meningiomas, 6 fibrous  (fibroblastic), 4 transitional (mixed), 1 psammoma-  tous, 4 angiomatous), Grade 2 =2, Grade 3= 2 | PCR-based LOH analysis of the APC gene using RFLP at two polymorphic markers (RsaI in exon 11 and MspI in exon 15); assessment of APC protein status (loss of wild-type vs mutant/aberrant APC) by immunostaining; correlation of APC genetic changes with nuclear β-catenin localization using χ² statistics | APC LOH was found in 15/32 informative tumors (47%): 7 showed LOH at both markers (exons 11 and 15), 4 only at exon 11, and 4 only at exon 15. By subtype, APC LOH occurred in 46% of meningothelial, 33% of fibrous, 75% of mixed/transitional, 75% of angiomatous meningiomas, and in a single psammomatous case, while no LOH was detected in atypical or anaplastic meningiomas. Tumors with APC LOH showed either loss of wild-type APC protein expression or expression of mutant APC, with a highly significant association between APC genetic status and APC protein pattern (χ² = 13.81, df = 2, P < 0.001). Nuclear β-catenin localization was significantly associated with APC genetic alterations (χ² = 21.96, df = 2, P < 0.0001), supporting involvement of APC–β-catenin pathway deregulation in a subset of meningiomas, particularly benign histological subtypes. | [4] |
| Human tumor cohort | 49 meningiomas | grade 1 = 41, grade 2 = 3, grade 3 = 5 | Immunohistochemistry for E-cadherin and β-catenin; immunoreactive score (IRS) based on staining intensity (0–3) × proportion of positive cells (0–4); classification by WHO 2007 grade; correlations with invasion, peritumoral edema (Goldman grade 0–3), and postoperative recurrence using χ² tests | E-cadherin positivity decreased from 92.7% in grade 1 to 33.3% in grade 2 and 0% in grade 3; β-catenin positivity decreased from 82.9% in grade 1 to 33.3% in grade 2 and 20% in grade 3. Reduced E-cadherin/β-catenin expression was significantly associated with higher grade, invasive growth, increased peritumoral edema, and postoperative recurrence. | [5] |
| Human tumor cohort | 153 tumors | grade 1 = 131, grade 2 = 19, grade 3 = 3 | Immunohistochemistry (IHC) for β-catenin and galectin-3 | β-catenin staining was strongest in grade 1 and decreased in grades 2–3, whereas galectin-3 showed the opposite trend, being higher in aggressive tumors; inverse association of total β-catenin with grade. | [6] |
| Human tumor cohort (in situ CSC/Wnt marker mapping) | 15 tumors | grade 1 = 8; grade 2/3 = 7 | Immunofluorescence of consecutive sections: CD133, SOX2, Nestin, Vimentin, GFAP, β3-tubulin, FZD9, SSEA4, OLIG2, Ki-67; single/double-marker stats | FZD9 and GFAP were significantly higher in grade 2/3 vs grade 1 (p < 0.01). Vimentin+FZD9 co-staining increased with grade; 3-D spatial analysis showed greater hetero-regional expression in higher grades. | [7] |
| Human tumor cohort | 154 intracranial meningiomas | 104 low-grade (G1) and 50 high-grade (G2–G3) meningiomas. PTBE was present in 103/154 cases and was graded separately by Steinhoff classification as PTBE grade 1 = 57, PTBE grade 2 = 44, and PTBE grade 3 = 2. | Immunohistochemistry for N-cadherin and β-catenin on tumor tissue; PTBE graded 0–3 using Steinhoff classification; marker expression graded by positive ratio of immunoreactive cells; results analyzed statistically | Positive N-cadherin membranous expression was seen in 50/104 (48.1%) low-grade and 34/50 (68%) high-grade tumors. In low-grade meningiomas, β-catenin expression was observed in the cytoplasm and nucleus in 54/104 (51.9%) cases, whereas in high-grade meningiomas, β-catenin expression was observed in 33/50 (66%) tumors only within the nucleus. PTBE was present in 103 cases; N-cadherin expression occurred in 36 PTBE grade 1, 28 grade 2, and 2 grade 3 cases, while β-catenin expression occurred in 40 PTBE grade 1, 24 grade 2, and 2 grade 3 cases, with statistically significant correlations between PTBE degree and both markers, most pronounced in the high-grade group | [8] |
| Human tumor cohort | 91 tumors | grade 1 = 43, grade 2 = 34, grade 3 = 14 | IHC for FOXM1, β-catenin, Ki-67 | FOXM1 increased with grade and correlated with proliferation and recurrence. β-catenin staining was strongest in grade 1 and lower in grades 2–3; reduced β-catenin associated with recurrence. | [9] |
| Human tumor cohort | 72 tumors | grade 1 = 49, grade 2 = 17, grade 3 = 6 | IHC for total and active (non-phospho) β-catenin, E-cadherin, N-cadherin, SNAIL, SLUG, TWIST1; LOH/MSI and Sanger sequencing | Total and active (non-phospho) β-catenin and SNAIL/SLUG expression increased with grade. N-cadherin expression was more pronounced than E-cadherin expression overall; however, neither E-cadherin nor N-cadherin protein expression was significantly associated with grade. | [10] |
| Human tumor cohort | 33 tumors | grade 1 = 16, grade 2 = 12, grade 3 = 5 | DVL1 microsatellite analysis in 33 tumors; HRM with Sanger sequencing of the PDZ-region in 26 available tumor DNA samples; IHC/H-score assessment of cytoplasmic and nuclear DVL1 and active β-catenin in 23 tumors | Nuclear DVL1 expression correlated with higher tumor grade and elevated active β-catenin; cytoplasmic DVL1 showed no association. Indicates grade-dependent nuclear accumulation of DVL1. | [11] |

**References**

1. **Shimada S, Ishizawa K, Hirose T. Expression of E-cadherin and catenins in meningioma: ubiquitous expression and its irrelevance to malignancy. Pathol Int. 2005 Jan;55(1):1-7.** [**https://doi.org/10.1111/j.1440-1827.2005.01786.x**](https://doi.org/10.1111/j.1440-1827.2005.01786.x)**.**
2. **Utsuki S, Oka H, Sato Y, Kawano N, Tsuchiya B, Kobayashi I, Fujii K. Invasive meningioma is associated with a low expression of E-cadherin and beta-catenin. Clin Neuropathol. 2005 Jan-Feb;24(1):8-12.**
3. **Brunner EC, Romeike BFM, Jung M, Comtesse N, Meese E. Altered expression of beta-catenin/E-cadherin in meningiomas. Histopathology. 2006 Aug;49(2):178-87.** [**https://doi.org/10.1111/j.1365-2559.2006.02440.x**](https://doi.org/10.1111/j.1365-2559.2006.02440.x)**.**
4. **Pećina-Slaus N, Nikuseva Martić T, Tomas D, Beros V, Zeljko M, Cupić H. Meningiomas exhibit loss of heterozygosity of the APC gene. J Neurooncol. 2008 Mar;87(1):63-70.** [**https://doi.org/10.1007/s11060-007-9500-6**](https://doi.org/10.1007/s11060-007-9500-6)**.**
5. **Zhou K, Wang G, Wang Y, Jin H, Yang S, Liu C. The potential involvement of E-cadherin and beta-catenins in meningioma. PLoS One. 2010 Jun 21;5(6):e11231.** [**https://doi.org/10.1371/journal.pone.0011231**](https://doi.org/10.1371/journal.pone.0011231)**.**
6. **Ahmed RA, Shebl AM, Habashy HO. Expression levels of β-catenin and galectin-3 in meningioma and their effect on brain invasion and recurrence: a tissue microarray study. Cancer Biol Med. 2017 Aug;14(3):319-326.** [**https://doi.org/10.20892/j.issn.2095-3941.2017.0024**](https://doi.org/10.20892/j.issn.2095-3941.2017.0024)**.**
7. **Alamir H, Alomari M, Salwati AAA, Saka M, Bangash M, Baeesa S, Alghamdi F, Carracedo A, Schulten HJ, Chaudhary A, Abuzenadah A, Hussein D. In situ characterization of stem cells-like biomarkers in meningiomas. Cancer Cell Int. 2018 May 25;18:77.** [**https://doi.org/10.1186/s12935-018-0571-6**](https://doi.org/10.1186/s12935-018-0571-6)**.**
8. **Rutkowski R, Chrzanowski R, Trwoga M, Kochanowicz J, Turek G, Mariak Z, Reszeć J. Expression of N-cadherin and β-catenin in human meningioma in correlation with peritumoral edema. Int J Neurosci. 2018 Sep;128(9):805-810.** [**https://doi.org/10.1080/00207454.2018.1424153**](https://doi.org/10.1080/00207454.2018.1424153)**.**
9. **Ibrahim, R. A., Shash, L. S., & Elgohary, S. A. (2021). FOXM1 and β-catenin in meningiomas: biomarkers for tumor proliferation and progression. Egyptian Journal of Pathology, 41(2), 137–145.** [**https://doi.org/10.4103/egjp.egjp_44_21**](https://doi.org/10.4103/egjp.egjp_44_21)**.**
10. **Bukovac, A., Kafka, A., Raguž, M., Brlek, P., Dragičević, K., Müller, D., & Pećina-Šlaus, N. (2021). Are we benign? What can Wnt signaling pathway and epithelial to mesenchymal transition tell us about intracranial meningioma progression. Cancers, 13(7), 1633.** [**https://doi.org/10.3390/cancers13071633**](https://doi.org/10.3390/cancers13071633)**.**
11. **Bukovac, A., Dragičević, K., Kafka, A., Orešković, D., Cesarec-Augustinović, S., & Pećina-Šlaus, N. (2021). Decoding the Role of DVL1 in Intracranial Meningioma. International Journal of Molecular Sciences, 22(21), 11996.** [**https://doi.org/10.3390/ijms222111996**](https://doi.org/10.3390/ijms222111996)**.**
