# Supplementary material for: The Wnt signaling networks in higher-grade meningiomas: from molecular pathogenesis to therapeutic innovation
Source: Cancer Cell Int. 2026 Jul 24;26:345. doi: 10.1186/s12935-026-04393-1 (PMC13685165; doi:10.1186/s12935-026-04393-1)
Supplement: Supplementary file 2 [file 12935_2026_4393_MOESM2_ESM.docx]

**Supplementary Figure 1.** Wnt signaling-related genes and associated genetic, epigenetic, and miRNA alterations reported across cancer types. Red, green, and yellow indicate genetic, epigenetic, and miRNA-associated alterations, respectively. The meningioma row is highlighted.
