# Supplementary material for: The Wnt signaling networks in higher-grade meningiomas: from molecular pathogenesis to therapeutic innovation
Source: Cancer Cell Int. 2026 Jul 24;26:345. doi: 10.1186/s12935-026-04393-1 (PMC13685165; doi:10.1186/s12935-026-04393-1)
Supplement: Supplementary file 3 [file 12935_2026_4393_MOESM3_ESM.docx]

**Supplementary Table 3**. Preclinical Wnt-pathway–targeting agents and modulators across disease models. This table summarizes reported preclinical studies of agents targeting or modulating components of the Wnt signaling network, including RSPO, DKK1, tankyrase, GSK3β, CK1α, Wnt/FZD, ROR, DVL, PORCN, β-catenin, β-catenin/CBP, β-catenin/TCF, and COX-associated Wnt modulation. For each agent, the table lists the target protein or axis, inhibitor/modulator name, therapeutic class, canonical or non-canonical Wnt branch, disease context, experimental model, reported biological or therapeutic effect, and supporting references. The evidence is largely preclinical and includes cell-line models, xenografts, patient-derived xenografts, organoids, syngeneic models, and selected non-oncology disease models

| **Protein** | **Inhibitor** | **Type** | **Wnt Signaling Pathway** | **Medical condition** | **Preclinical study** | **Result** | **Reference** |
| --- | --- | --- | --- | --- | --- | --- | --- |
| RSPO | OMP-131R10 (Rosmantuzumab) | Humanized monoclonal antibody, anti-RSPO3, Wnt pathway inhibitor | Canonical | Acute myeloid leukemia (AML), Fibrosis | AML patient-derived xenografts, Mouse models | Impairs self-renewal and induces differentiation, antifibrotic activity | [1, 2] |
| DKK1 | BHQ880 | Monoclonal antibody, anti-DKK1 | Canonical | multiple myeloma, Osteosarcoma | Mouse xenograft | Tumor reduction, metastasis reduction | [3, 4] |
| DKK1 | DKN-01 | Monoclonal antibody, anti-DKK1 | Canonical | Ovarian cancer | Human ovarian cancer cell lines, ID8 syngeneic mouse model | No significant change in tumor growth in the ID8 model and partial reversal of immune suppression | [5, 6] |
| Tankyrase | XAV939 | Small molecule, tankyrase 1/2 inhibitor, Wnt pathway inhibitor | Canonical | Myocardial infarction, Medulloblastoma, Lung adenocarcinoma, Neuroblastoma, colorectal cancer, melanoma, breast cancer, cervical cancer | Rat myocardial infarction model/ in vitro rat cardiomyocytes, Human medulloblastoma cell lines, Human A549 lung adenocarcinoma cell line, Human neuroblastoma cell line | Improved cardiac function/reduced infarct size/ decreased fibrosis,Reduced cell proliferation and survival, Inhibited cell proliferation and migration, induced apoptosis | [7 - 14] |
| Tankyrase | IWR-1 | Small molecule, tankyrase 1/2 inhibitor, Wnt pathway inhibitor | Canonical | Colorectal cancer, Osteosarcoma | Cell line, xenograft | Inhibited EMT, in vivo growth | [15, 16] |
| Tankyrase | NVP-TNKS656 | Small molecule, selective tankyrase 2 inhibitor, Wnt pathway inhibitor | Canonical | Colorectal cancer, Hepatocellular carcinoma | Cell line | Reduced tumor growth, Inhibited proliferation, migration, and invasion | [17, 18] |
| Tankyrase | LZZ-02 | Wnt signaling inhibitor (small molecule) | Canonical | colonic carcinoma | Cell line, xenograft | Reduced cell growth, tumor shrinkage | [19] |
| Tankyrase | E7449 | Wnt pathway inhibitor (small molecule) | Canonical | Breast cancer, Lung adenocarcinoma, | Cell line, xenograft | Reduced tumor growth | [20, 21] |
| Tankyrase | G007-LK | Tankyrase inhibitor | Canonical | Non-small cell lung cancer (NSCLC), hepatocellular carcinoma (HCC), Glioma, Colorectal cancer | Cell line, xenograft | Enhanced sensitivity to other drugs, reduced proliferation | [22 - 25] |
| Tankyrase | JW55 | Tankyrase inhibitor | Canonical | Colon cancer; papillary thyroid carcinoma (PTC-associated in silico analysis) | Colon carcinoma cell lines and conditional APC-mutant mice; GSCALite/CTRP pharmacogenomic drug-sensitivity correlation analysis | Inhibited canonical Wnt signaling and reduced tumor growth/polyposis in the APC-mutant model; high ADORA1 expression was associated with greater JW-55 sensitivity in CTRP-based analysis | [26, 27] |
| Tankyrase | AZ1366 | Tankyrase inhibitor, Wnt pathway inhibitor | Canonical | Non-small cell lung cancer (NSCLC), Colorectal cancer | Cell line, mouse model | Synergistic effect with other drugs | [28, 29] |
| Tankyrase | MSC2504877 | Wnt pathway inhibitor | Canonical | Colorectal cancer | Cell line | Reduced cell growth, synergistic effect with other drugs | [30] |
| Tankyrase | JW67 | Tankyrase inhibitor | Canonical | Colorectal cancer, | Cell line, mouse xenograft models, | Tumor growth inhibition | [31] |
| Tankyrase | JW74 | Tankyrase inhibitor | Canonical | Colorectal cancer, | Cell line, mouse xenograft models, | Tumor growth inhibition | [31] |
| GSK3β | 9-ING-41 | GSK-3β inhibitor, anti-cancer agent | Canonical | Colorectal cancer, Neuroblastoma, bladder cancer, Melanoma, Renal cancer, Glioblastoma, breast cancer, pancreatic cancer, lymphoma, soft-tissue sarcoma | Cell line/tumor organoid model, xenograft, patient derived xenograft models | Reduced cell growth, synergistic effect with other drugs | [32 - 41] |
| GSK3β | LY2090314 | GSK-3 inhibitor | Wnt/β-catenin-associated, context-dependent modulation. | Neuroblastoma, melanoma, Lung cancer, prostate cancer, Adult T-cell leukemia, pancreatic cancer | Cell line, zebra fish, mouse models, | Antiproliferative and apoptotic effects | [42 - 46] |
| CK1α | Pyrvinium pamoate | Small molecule, Wnt pathway inhibitor | Canonical | Anaplastic thyroid cancer, colorectal cancer, KRAS-mutant lung cancer, breast cancer, pancreatic cancer, Gastric cancer, ovarian cancer, Malignant mesothelioma, Merkel cell carcinoma, Renal cell carcinoma, uveal melanoma, osteosarcoma, AML, Wilms Tumor, cervical cancer, oral squamous cell carcinoma, lymphoma, prostate cancer | Cell line, mouse xenograft, 2d and 3d cell culture models, organoids | Overcame resistance to other drugs, reduced proliferation, metabolic lethality | [47 - 64] |
| CK1α | SSTC3 | Small molecule, Wnt antagonist | Canonical | Colorectal cancer, Systemic lupus erythematosus, Medulloblastoma, | Mouse, patient derived cells, patient derived xenograft, | repressed the systemic autoinflammatory responses, attenuated growth and metastasis | [65 - 67] |
| Wnt/FZD | OMP-54F28 (Ipafricept) | Fusion protein, Wnt signaling inhibitor | Canonical | Breast Cancer, pancreatic cancer | In vivo mouse models | Reduced tumor growth | [68] |
| Wnt/FZD | OMP-18R5 (Vantictumab) | Monoclonal antibody, anti-FZD (Frizzled), Wnt pathway inhibitor | Canonical | lung NSCLC, pancreatic tumor, teratocarcinoma, pancreatic, breast tumor, colon tumor | Patient derived xenograft | Reduced tumor growth | [69] |
| Wnt/FZD | WNT5A/Foxy-5 axis modulation | Wnt signaling agonist peptide | Non-canonical | Prostate cancer, breast cancer, AML, colon cancer, | Cell lines, xenograft | Reduced metastasis, ROS generation, cell proliferation, and autophagy | [70 - 74] |
| Wnt/FZD | OTSA-101 | Targeted therapy antibody | Canonical | Synovial sarcoma | Xenograft Models | Reduced tumor growth, long survival | [75] |
| Wnt/FZD | Fz7-21 | Frizzled-targeting peptide antagonist | Canonical | Triple negative breast cancer | TNBC cancer stem cells (CSCs) | synergized with carboplatin to improve its therapeutic efficacy | [76] |
| Wnt/FZD | IC-2 | Wnt pathway inhibitor | Canonical | Colorectal cancer, liver cancer | Cell line, xenograft | Reduced transcriptional activity and cancer stem cell properties in DLD-1 colorectal cancer cells, synergistically enhancing 5-FU's cytotoxicity | [77, 78] |
| ROR | Cirmtuzumab | Monoclonal antibody, ROR1 inhibitor | Non-canonical | chronic lymphocytic leukemia, Mantle cell lymphoma (MCL), breast cancer | patient derived cells/xenograft, in vivo mouse models. | reduced proliferation, metastasis, stemness , synergy with other drugs | [79 - 81] |
| ROR | ROR1R-CART | CAR-T cell therapy targeting ROR1 | Non-canonical | B-cell malignancies | Cell line | tumor cell lysis, high specificity | [82] |
| ROR | NBE-002 | ROR1-targeting antibody–drug conjugate | Non-canonical | High-grade serous ovarian cancer (HGSOC) | Cell line, mouse, PDX | Growth inhibition, apoptosis, synergy with other drugs | [83] |
| ROR | VLS-101 (Zilovertamab Vedotin) | Antibody-drug conjugate targeting ROR1 | Non-canonical | Mantle cell lymphoma (MCL) | Cell lines, PDX models | Tumor Regression in combination therapy | [84] |
| ROR | KAN0439834 | Small molecule, cancer drug | Non-canonical | Pancreatic carcinoma, Chronic lymphocytic leukemia (CLL) | Cell lines, patient cells, xenograft mice | Significant apoptosis | [85, 86] |
| DVL | FJ9 | Wnt signaling inhibitor candidate | Canonical | Melanoma, NSCLC | Cell lines, xenograft mouse models | Reduced tumor growth, induced apoptosis | [87] |
| DVL | 3289-8625 | Experimental Wnt pathway modulator | Canonical | Ovarian cancer, Lung cancer | Cell line | Resensitized to Paclitaxel, cisplatin | [88, 89] |
| DVL | Sulindac | NSAID, Wnt pathway modulator | Canonical | Ovarian cancer | Cell line | Antiproliferative, synergy with other drugs | [90] |
| PORCN | WNT974 (LGK974) | Porcupine inhibitor, Wnt pathway inhibitor | Canonical | Clear Cell Renal Cell Carcinoma, Colorectal cancer, Lung cancer, Glioblastoma,neuroendocrine prostate cancer, squamous cell carcinoma, | Cell line, allograft mice, organoid, xenograft mouse models | Inhibit proliferation, reverse resistance to other drugs | [91 - 96] |
| PORCN | Wnt-C59 | Porcupine inhibitor, Wnt pathway inhibitor | Canonical | Nasopharyngeal carcinoma (NPC), WNT-driven mammary cancer, pancreatic cancer | Cell line, mouse xenograft | Suppressed tumor growth, reduced stemness, increased sensitivity to other drugs | [97 - 99] |
| PORCN | CGX1321 | Porcupine inhibitor | Canonical | Ovarian cancer | Cancer Mouse models | Reduced tumor growth, improved survival | [100] |
| PORCN | IWP-2 | Porcupine inhibitor | Canonical | Colorectal cancer, breast cancer, Gastric cancer, Head and neck squamous cell carcinoma (HNSCC), prostate cancer, Bladder cancer | Cell line | Inhibition of b catenin signaling, Reduced proliferation, invasion, reduced aggressiveness, induced apoptosis | [101 - 106] |
| PORCN | ETC-159 | Porcupine inhibitor | Canonical | Osteosarcoma, colorectal cancer, Pancreatic ductal adenocarcinoma (PDAC) | Cell line, Murine xenograft, patient derived xenograft | Increased tumor necrosis, reduced proliferation and stemness, effectiveness with combination therapy | [107 - 109] |
| PORCN | RXC004 | Porcupine inhibitor | Canonical | gastrointestinal cancers (colorectal, pancreatic) | Cell lines, xenograft models, murine syngeneic tumor models | Inhibited proliferation and tumor growth, promoted differentiation, reduced immune suppression, synergized with anti-PD-1 | [110] |
| PORCN | GNF-6231 | Wnt pathway inhibitor | Canonical | Wnt-driven cancers (MMTV-WNT1 mammary tumor model) | Mouse MMTV-WNT1 xenograft model | Strong Wnt pathway inhibition, robust antitumor efficacy | [111] |
| β-catenin | CWP232291 | Wnt pathway inhibitor | Canonical | Ovarian cancer, prostate cancer, intestinal cancer, Salivary gland adenoid cystic carcinoma (ACC) | Cell line, patient derived organoid, mice | Reduced tumor growth, activation of apoptosis, induced cytotoxicity | [112 - 115] |
| β-catenin | KYA1797K | Wnt/β-catenin pathway inhibitor | Canonical | Pancreatic cancer, KRAS-mutated non-small cell lung cancer (NSCLC),Triple-negative breast cancer (TNBC), colorectal cancer, | Cell line, mouse models, patient derived cells/organoid, xenografts, | Suppressed invasion and migration, reduced tumor growth and transformation, reduced proliferation | [116 - 119] |
| β-catenin | ST316 | Experimental Wnt inhibitor | Canonical | Breast Cancer | Mouse models | Tumor growth inhibition | [120] |
| β-catenin | FH535 | Wnt/β-catenin inhibitor | Canonical | Esophageal squamous cell carcinoma, Myeloid leukemia, Non-small cell lung cancer (NSCLC), pancreatic cancer, breast cancer, Gastric cancer, colorectal cancer, | Cell line, xenograft models, | Reduced EMT, increased radiosensitivity, reduced proliferation alone and in combination, reduced cancer stemness | [121 - 127] |
| β-catenin/CBP | PRI-724 | CBP/β-catenin interaction inhibitor | Canonical | Head and neck squamous cell carcinoma (HNSCC), Hepatocellular carcinoma (HCC), Triple-negative breast cancer (TNBC), | Cell line, xenograft mouse models, | Reduced migration/proliferation, enhanced apoptosis with combination therapy, overcame therapy resistance, | [128 - 130] |
| β-catenin/CBP | ICG001 | CBP/β-catenin interaction inhibitor | Canonical | **Sporadic meningioma,** HPV-positive head and neck squamous cell carcinoma (HNSCC), Castration-resistant prostate cancer (CRPC), T-cell acute lymphoblastic leukemia (T-ALL), Cervical cancer, Gastric cancer, Uveal melanoma, Hepatocellular carcinoma (HCC), Mantle cell lymphoma (MCL), Endocrine-resistant breast cancer | Cell lines, tumor models, nude mouse xenograft, PDX mouse models, | Reduced proliferation and migration, induced apoptosis, restored sensitivity to other drugs, enhanced apoptosis with combination therapy, **selective growth inhibition in Merlin-low/NF2-mutant cells; robust G1 arrest** | [131 - 140] |
| β-catenin/CBP | E7386 | Wnt pathway inhibitor | Canonical | Hepatocellular carcinoma, Colorectal cancer, Head and neck cancer, | Cell line, patient derived organoid, mouse models, | Anti-tumor activity with combination with other drugs, synergy with other drugs, | [141 - 143] |
| β-catenin/CBP | Isoquercitrin | Natural flavonoid, Wnt pathway modulator | Canonical | Colon cancer | Cell line | In vitro colon cancer cell growth suppression | [144] |
| β-catenin/TCF | PKF115-584 | Experimental Wnt signaling inhibitor | Canonical | Acute myeloid leukemia (AML), Chronic lymphocytic leukemia (CLL), Hepatocellular carcinoma, | Cell line, xenograft mouse models | Induced apoptosis with combination therapy, reduced tumor growth, | [145-147] |
| β-catenin/TCF | CGP049090 | Wnt pathway inhibitor | Canonical | Chronic lymphocytic leukemia (CLL), Hepatocellular carcinoma, | Cell line, xenograft mouse models | Induced apoptosis with combination therapy, reduced tumor growth, | [146, 147] |
| β-catenin/TCF | iCRT3 | β-catenin/TCF interaction inhibitor | Canonical | Head and neck cancer, colorectal cancer, | Cell line, | Induced cytotoxicity and apoptosis, caused cell cycle arrest, reduced Wnt target gene expression, inhibited migration | [148, 149] |
| β-catenin/TCF | LF3 | β-catenin/TCF interaction inhibitor | Canonical | Colon cancer, Gastric cancer | Cancer stem cells, cancer xenograft mouse models, mouse models, | reduced motility and proliferation, suppressed tumor growth, synergistic effect with other drugs, | [150, 151] |
| COX | Aspirin | NSAID, potential Wnt modulator | Canonical | Colon cancer, | Cell line, | Reduced EMT, viability and motility | [152] |
| COX | Celecoxib | NSAID, potential Wnt modulator | Canonical | Breast cancer, Colon cancer, Glioblastoma multiforme (GBM) | Cell line, mouse models, | reduced chemo-resistance, suppressed EMT, decreased metastasis/tumorigenesis, induced apoptosis and oxidative stress with combination therapy | [153-155] |

**References**

1. Salik B, Yi H, Hassan N, Santiappillai N, Vick B, Connerty P, Duly A, Trahair T, Woo AJ, Beck D, Liu T, Spiekermann K, Jeremias I, Wang J, Kavallaris M, Haber M, Norris MD, Liebermann DA, D'Andrea RJ, Murriel C, Wang JY. Targeting RSPO3-LGR4 Signaling for Leukemia Stem Cell Eradication in Acute Myeloid Leukemia. Cancer Cell. 2020 Aug 10;38(2):263-278.e6.

<https://doi.org/10.1016/j.ccell.2020.05.014>.

2. Zhang M, Haughey M, Wang NY, Blease K, Kapoun AM, Couto S, Belka I, Hoey T, Groza M, Hartke J, Bennett B, Cain J, Gurney A, Benish B, Castiglioni P, Drew C, Lachowicz J, Carayannopoulos L, Nathan SD, Distler J, Brenner DA, Hariharan K, Cho H, Xie W. Targeting the Wnt signaling pathway through R-spondin 3 identifies an anti-fibrosis treatment strategy for multiple organs. PLoS One. 2020 Mar 11;15(3):e0229445. <https://doi.org/10.1371/journal.pone.0229445>.
3. Fulciniti M, Tassone P, Hideshima T, Vallet S, Nanjappa P, Ettenberg SA, Shen Z, Patel N, Tai YT, Chauhan D, Mitsiades C, Prabhala R, Raje N, Anderson KC, Stover DR, Munshi NC. Anti-DKK1 mAb (BHQ880) as a potential therapeutic agent for multiple myeloma. Blood. 2009 Jul 9;114(2):371-9. <https://doi.org/10.1182/blood-2008-11-191577>.
4. Goldstein SD, Trucco M, Bautista Guzman W, Hayashi M, Loeb DM. A monoclonal antibody against the Wnt signaling inhibitor dickkopf-1 inhibits osteosarcoma metastasis in a preclinical model. Oncotarget. 2016 Apr 19;7(16):21114-23. <https://doi.org/10.18632/oncotarget.8522>.
5. Betella I, Turbitt WJ, Szul T, Wu B, Martinez A, Katre A, Wall JA, Norian L, Birrer MJ, Arend R. Wnt signaling modulator DKK1 as an immunotherapeutic target in ovarian cancer. Gynecol Oncol. 2020 Jun;157(3):765-774. https://doi.org/10.1016/j.ygyno.2020.03.010.
6. Wall JA, Meza-Perez S, Scalise CB, Katre A, Londoño AI, Turbitt WJ, Randall T, Norian LA, Arend RC. Manipulating the Wnt/β-catenin signaling pathway to promote anti-tumor immune infiltration into the TME to sensitize ovarian cancer to ICB therapy. Gynecol Oncol. 2021 Jan;160(1):285-294. <https://doi.org/10.1016/j.ygyno.2020.10.031>.
7. Zhang Z, Qi J, Fan X, Pan M. XAV939 Improves the Prognosis of Myocardial Infarction by Blocking the Wnt/β-Catenin Signalling Pathway. Appl Biochem Biotechnol. 2024 Feb;196(2):605-615. <https://doi.org/10.1007/s12010-023-04485-y>.
8. Renna C, Salaroli R, Cocchi C, Cenacchi G. XAV939-mediated ARTD activity inhibition in human MB cell lines. PLoS One. 2015 Apr 2;10(4):e0124149. <https://doi.org/10.1371/journal.pone.0124149>.
9. Li C, Zheng X, Han Y, Lv Y, Lan F, Zhao J. XAV939 inhibits the proliferation and migration of lung adenocarcinoma A549 cells through the WNT pathway. Oncol Lett. 2018 Jun;15(6):8973-8982. <https://doi.org/10.3892/ol.2018.8491>.
10. Tian X, Hou W, Bai S, Fan J, Tong H, Bai Y. XAV939 promotes apoptosis in a neuroblastoma cell line via telomere shortening. Oncol Rep. 2014 Nov;32(5):1999-2006. <https://doi.org/10.3892/or.2014.3460>.
11. Kim D, Koh B, Kim KR, Kim KY, Jung WH, Kim HY, Kim S, Dal Rhee S. Anticancer effect of XAV939 is observed by inhibiting lactose dehydrogenase A in a 3-dimensional culture of colorectal cancer cells. Oncol Lett. 2019 Nov;18(5):4858-4864. <https://doi.org/10.3892/ol.2019.10813>.
12. Shetti D, Zhang B, Fan C, Mo C, Lee BH, Wei K. Low Dose of Paclitaxel Combined with XAV939 Attenuates Metastasis, Angiogenesis and Growth in Breast Cancer by Suppressing Wnt Signaling. Cells. 2019 Aug 14;8(8):892. <https://doi.org/10.3390/cells8080892>.
13. Zhang J, Si J, Gan L, Guo M, Yan J, Chen Y, Wang F, Xie Y, Wang Y, Zhang H. Inhibition of Wnt signalling pathway by XAV939 enhances radiosensitivity in human cervical cancer HeLa cells. Artif Cells Nanomed Biotechnol. 2020 Dec;48(1):479-487. https://doi.org/10.1080/21691401.2020.1716779.
14. Antony F, Kang X, Pundkar C, Wang C, Mishra A, Chen P, Babu RJ, Suryawanshi A. Targeting β-catenin using XAV939 nanoparticle promotes immunogenic cell death and suppresses conjunctival melanoma progression. Int J Pharm. 2023 Jun 10;640:123043. <https://doi.org/10.1016/j.ijpharm.2023.123043>.
15. Lee SC, Kim OH, Lee SK, Kim SJ. IWR-1 inhibits epithelial-mesenchymal transition of colorectal cancer cells through suppressing Wnt/β-catenin signaling as well as survivin expression. Oncotarget. 2015 Sep 29;6(29):27146-59. <https://doi.org/10.18632/oncotarget.4354>.
16. Martins-Neves SR, Paiva-Oliveira DI, Fontes-Ribeiro C, Bovée JVMG, Cleton-Jansen AM, Gomes CMF. IWR-1, a tankyrase inhibitor, attenuates Wnt/β-catenin signaling in cancer stem-like cells and inhibits in vivo the growth of a subcutaneous human osteosarcoma xenograft. Cancer Lett. 2018 Feb 1;414:1-15. <https://doi.org/10.1016/j.canlet.2017.11.004>.
17. Yun H, You JE, Hong JK, Kim DY, Lee JU, Kang DH, Ryu YS, Koh DI, Jin DH. TCOF1 promotes the colorectal cancer progression by stabilizing β-catenin. Med Oncol. 2023 Nov 7;40(12):348. <https://doi.org/10.1007/s12032-023-02218-z>.
18. Huang J, Qu Q, Guo Y, Xiang Y, Feng D. Tankyrases/β-catenin Signaling Pathway as an Anti-proliferation and Anti-metastatic Target in Hepatocarcinoma Cell Lines. J Cancer. 2020 Jan 1;11(2):432-440. <https://doi.org/10.7150/jca.30976>.
19. Li B, Liang J, Lu F, Zeng G, Zhang J, Ma Y, Liu P, Wang Q, Zhou Q, Chen L. Discovery of Novel Inhibitor for WNT/β-Catenin Pathway by Tankyrase 1/2 Structure-Based Virtual Screening. Molecules. 2020 Apr 6;25(7):1680. <https://doi.org/10.3390/molecules25071680>.
20. McGonigle S, Chen Z, Wu J, Chang P, Kolber-Simonds D, Ackermann K, Twine NC, Shie JL, Miu JT, Huang KC, Moniz GA, Nomoto K. E7449: A dual inhibitor of PARP1/2 and tankyrase1/2 inhibits growth of DNA repair deficient tumors and antagonizes Wnt signaling. Oncotarget. 2015 Dec 1;6(38):41307-23. <https://doi.org/10.18632/oncotarget.5846>.
21. Zheng X, Han Y, Gu L, Gao S, Lv Y, Li C. Study of the mechanism by which Xiaoyan decoction combined with E7449 regulates tumorigenesis in lung adenocarcinoma. J Cell Mol Med. 2024 Jun;28(12):e18467. <https://doi.org/10.1111/jcmm.18467>.
22. Shiraishi A, Oh-Hara T, Takahashi Y, Uchibori K, Nishio M, Katayama R. 3D layered co-culture model enhances Trastuzumab Deruxtecan sensitivity and reveals the combined effect with G007-LK in HER2-positive non-small cell lung cancer. Biochem Biophys Res Commun. 2024 Sep 17;725:150255. <https://doi.org/10.1016/j.bbrc.2024.150255>.
23. Liang B, Wang H, Qiao Y, Wang X, Qian M, Song X, Zhou Y, Zhang Y, Shang R, Che L, Chen Y, Huang Z, Wu H, Monga SP, Zeng Y, Calvisi DF, Chen X, Chen X. Differential requirement of Hippo cascade during CTNNB1 or AXIN1 mutation-driven hepatocarcinogenesis. Hepatology. 2023 Jun 1;77(6):1929-1942. <https://doi.org/10.1002/hep.32693>.
24. Kierulf-Vieira KS, Sandberg CJ, Waaler J, Lund K, Skaga E, Saberniak BM, Panagopoulos I, Brandal P, Krauss S, Langmoen IA, Vik-Mo EO. A Small-Molecule Tankyrase Inhibitor Reduces Glioma Stem Cell Proliferation and Sphere Formation. Cancers (Basel). 2020 Jun 19;12(6):1630. <https://doi.org/10.3390/cancers12061630>.
25. Lau T, Chan E, Callow M, Waaler J, Boggs J, Blake RA, Magnuson S, Sambrone A, Schutten M, Firestein R, Machon O, Korinek V, Choo E, Diaz D, Merchant M, Polakis P, Holsworth DD, Krauss S, Costa M. A novel tankyrase small-molecule inhibitor suppresses APC mutation-driven colorectal tumor growth. Cancer Res. 2013 May 15;73(10):3132-44. <https://doi.org/10.1158/0008-5472.CAN-12-4562>.
26. Waaler J, Machon O, Tumova L, Dinh H, Korinek V, Wilson SR, Paulsen JE, Pedersen NM, Eide TJ, Machonova O, Gradl D, Voronkov A, von Kries JP, Krauss S. A novel tankyrase inhibitor decreases canonical Wnt signaling in colon carcinoma cells and reduces tumor growth in conditional APC mutant mice. Cancer Res. 2012 Jun 1;72(11):2822-32. <https://doi.org/10.1158/0008-5472.CAN-11-3336>.
27. Lin X, Wang ZY, Xue G, Qin XJ, Wu JF, Zhang G. ADORA1 is a diagnostic-related biomarker and correlated with immune infiltrates in papillary thyroid carcinoma. J Cancer. 2021 May 13;12(13):3997-4010. <https://doi.org/10.7150/jca.50743>.
28. Scarborough HA, Helfrich BA, Casás-Selves M, Schuller AG, Grosskurth SE, Kim J, Tan AC, Chan DC, Zhang Z, Zaberezhnyy V, Bunn PA, DeGregori J. AZ1366: An Inhibitor of Tankyrase and the Canonical Wnt Pathway that Limits the Persistence of Non-Small Cell Lung Cancer Cells Following EGFR Inhibition. Clin Cancer Res. 2017 Mar 15;23(6):1531-1541. <https://doi.org/10.1158/1078-0432.CCR-16-1179>.
29. Quackenbush KS, Bagby S, Tai WM, Messersmith WA, Schreiber A, Greene J, Kim J, Wang G, Purkey A, Pitts TM, Nguyen A, Gao D, Blatchford P, Capasso A, Schuller AG, Eckhardt SG, Arcaroli JJ. The novel tankyrase inhibitor (AZ1366) enhances irinotecan activity in tumors that exhibit elevated tankyrase and irinotecan resistance. Oncotarget. 2016 May 10;7(19):28273-85. https://doi.org/10.18632/oncotarget.8626.
30. Menon M, Elliott R, Bowers L, Balan N, Rafiq R, Costa-Cabral S, Munkonge F, Trinidade I, Porter R, Campbell AD, Johnson ER, Esdar C, Buchstaller HP, Leuthner B, Rohdich F, Schneider R, Sansom O, Wienke D, Ashworth A, Lord CJ. A novel tankyrase inhibitor, MSC2504877, enhances the effects of clinical CDK4/6 inhibitors. Sci Rep. 2019 Jan 17;9(1):201. https://doi.org/10.1038/s41598-018-36447-4.
31. Waaler J, Machon O, von Kries JP, Wilson SR, Lundenes E, Wedlich D, Gradl D, Paulsen JE, Machonova O, Dembinski JL, Dinh H, Krauss S. Novel synthetic antagonists of canonical Wnt signaling inhibit colorectal cancer cell growth. Cancer Res. 2011 Jan 1;71(1):197-205. https://doi.org/10.1158/0008-5472.CAN-10-1282.
32. Poloznikov A, Nikulin S, Bolotina L, Kachmazov A, Raigorodskaya M, Kudryavtseva A, Bakhtogarimov I, Rodin S, Gaisina I, Topchiy M, Asachenko A, Novosad V, Tonevitsky A, Alekseev B. 9-ING-41, a Small Molecule Inhibitor of GSK-3β, Potentiates the Effects of Chemotherapy on Colorectal Cancer Cells. Front Pharmacol. 2021 Dec 9;12:777114. https://doi.org/10.3389/fphar.2021.777114.
33. Ugolkov AV, Bondarenko GI, Dubrovskyi O, Berbegall AP, Navarro S, Noguera R, O'Halloran TV, Hendrix MJ, Giles FJ, Mazar AP. 9-ING-41, a small-molecule glycogen synthase kinase-3 inhibitor, is active in neuroblastoma. Anticancer Drugs. 2018 Sep;29(8):717-724. https://doi.org/10.1097/CAD.0000000000000652.
34. Kuroki H, Anraku T, Kazama A, Bilim V, Tasaki M, Schmitt D, Mazar AP, Giles FJ, Ugolkov A, Tomita Y. 9-ING-41, a small molecule inhibitor of GSK-3beta, potentiates the effects of anticancer therapeutics in bladder cancer. Sci Rep. 2019 Dec 27;9(1):19977. https://doi.org/10.1038/s41598-019-56461-4.
35. Coats JT, Tauro S, Sutherland C. Elraglusib (formerly 9-ING-41) possesses potent anti-lymphoma properties which cannot be attributed to GSK3 inhibition. Cell Commun Signal. 2023 Jun 14;21(1):131. https://doi.org/10.1186/s12964-023-01147-8.
36. Shaw G, Cavalcante L, Giles FJ, Taylor A. Elraglusib (9-ING-41), a selective small-molecule inhibitor of glycogen synthase kinase-3 beta, reduces expression of immune checkpoint molecules PD-1, TIGIT and LAG-3 and enhances CD8+ T cell cytolytic killing of melanoma cells. J Hematol Oncol. 2022 Sep 14;15(1):134. https://doi.org/10.1186/s13045-022-01352-x.
37. Anraku T, Kuroki H, Kazama A, Bilim V, Tasaki M, Schmitt D, Mazar A, Giles FJ, Ugolkov A, Tomita Y. Clinically relevant GSK‑3β inhibitor 9‑ING‑41 is active as a single agent and in combination with other antitumor therapies in human renal cancer. Int J Mol Med. 2020 Feb;45(2):315-323. https://doi.org/10.3892/ijmm.2019.4427.
38. Ugolkov A, Qiang W, Bondarenko G, Procissi D, Gaisina I, James CD, Chandler J, Kozikowski A, Gunosewoyo H, O'Halloran T, Raizer J, Mazar AP. Combination Treatment with the GSK-3 Inhibitor 9-ING-41 and CCNU Cures Orthotopic Chemoresistant Glioblastoma in Patient-Derived Xenograft Models. Transl Oncol. 2017 Aug;10(4):669-678. https://doi.org/10.1016/j.tranon.2017.06.003.
39. Verbeke S, Perret R, Chaire V, Richard E, Velasco V, Giles F, Cavalcante L, Italiano A. GSK3-beta as a candidate therapeutic target in soft tissue sarcomas. J Hematol Oncol. 2021 Dec 2;14(1):202. https://doi.org/10.1186/s13045-021-01215-x.
40. Ugolkov A, Gaisina I, Zhang JS, Billadeau DD, White K, Kozikowski A, Jain S, Cristofanilli M, Giles F, O'Halloran T, Cryns VL, Mazar AP. GSK-3 inhibition overcomes chemoresistance in human breast cancer. Cancer Lett. 2016 Oct 1;380(2):384-392. https://doi.org/10.1016/j.canlet.2016.07.006.
41. Ding L, Madamsetty VS, Kiers S, Alekhina O, Ugolkov A, Dube J, Zhang Y, Zhang JS, Wang E, Dutta SK, Schmitt DM, Giles FJ, Kozikowski AP, Mazar AP, Mukhopadhyay D, Billadeau DD. Glycogen Synthase Kinase-3 Inhibition Sensitizes Pancreatic Cancer Cells to Chemotherapy by Abrogating the TopBP1/ATR-Mediated DNA Damage Response. Clin Cancer Res. 2019 Nov 1;25(21):6452-6462. https://doi.org/10.1158/1078-0432.CCR-19-0799. Epub 2019 Sep 18. Erratum in: Clin Cancer Res. 2021 Jul 15;27(14):4128. https://doi.org/10.1158/1078-0432.CCR-21-2114.
42. Kunnimalaiyaan S, Schwartz VK, Jackson IA, Clark Gamblin T, Kunnimalaiyaan M. Antiproliferative and apoptotic effect of LY2090314, a GSK-3 inhibitor, in neuroblastoma in vitro. BMC Cancer. 2018 May 11;18(1):560. https://doi.org/10.1186/s12885-018-4474-7.
43. Atkinson JM, Rank KB, Zeng Y, Capen A, Yadav V, Manro JR, Engler TA, Chedid M. Activating the Wnt/β-Catenin Pathway for the Treatment of Melanoma--Application of LY2090314, a Novel Selective Inhibitor of Glycogen Synthase Kinase-3. PLoS One. 2015 Apr 27;10(4):e0125028. https://doi.org/10.1371/journal.pone.0125028.
44. Santoro R, Zanotto M, Simionato F, Zecchetto C, Merz V, Cavallini C, Piro G, Sabbadini F, Boschi F, Scarpa A, Melisi D. Modulating TAK1 Expression Inhibits YAP and TAZ Oncogenic Functions in Pancreatic Cancer. Mol Cancer Ther. 2020 Jan;19(1):247-257. https://doi.org/10.1158/1535-7163.MCT-19-0270.
45. Fukuda K, Takeuchi S, Arai S, Kita K, Tanimoto A, Nishiyama A, Yano S. Glycogen synthase kinase-3 inhibition overcomes epithelial-mesenchymal transition-associated resistance to osimertinib in EGFR-mutant lung cancer. Cancer Sci. 2020 Jul;111(7):2374-2384. https://doi.org/10.1111/cas.14454.
46. Ishikawa C, Mori N. A New Strategy for Adult T-Cell Leukemia Treatment Targeting Glycogen Synthase Kinase-3β. Eur J Haematol. 2024 Dec;113(6):852-862. https://doi.org/10.1111/ejh.14300.
47. Li Y. Pyrvinium pamoate can overcome artemisinin's resistance in anaplastic thyroid cancer. BMC Complement Med Ther. 2021 May 28;21(1):156. https://doi.org/10.1186/s12906-021-03332-z.
48. Zheng W, Chen K, Lv Y, Lao W, Zhu H. Pyrvinium Pamoate Induces Cell Apoptosis and Autophagy in Colorectal Cancer. Anticancer Res. 2024 Mar;44(3):1193-1199. https://doi.org/10.21873/anticanres.16914.
49. Feng J, Jiang W, Liu Y, Huang W, Hu K, Li K, Chen J, Ma C, Sun Z, Pang X. Blocking STAT3 by pyrvinium pamoate causes metabolic lethality in KRAS-mutant lung cancer. Biochem Pharmacol. 2020 Jul;177:113960. https://doi.org/10.1016/j.bcp.2020.113960.
50. Serala K, Bai J, Prince S. Pyrvinium Pamoate Alone and With Gemcitabine Exhibits Anti-Pancreatic Cancer Activity in 2D and 3D Cell Culture Models. J Cell Mol Med. 2024 Dec;28(23):e70222. https://doi.org/10.1111/jcmm.70222.
51. Rodgers SJ, Ooms LM, Mitchell CA. The FDA-Approved Drug Pyrvinium Selectively Targets ER+ Breast Cancer Cells with High INPP4B Expression. Cancers (Basel). 2022 Dec 26;15(1):135. https://doi.org/10.3390/cancers15010135.
52. Ji Y, Zhang W, Shen K, Su R, Liu X, Ma Z, Liu B, Hu C, Xue Y, Xin Z, Yang Y, Li A, Jiang Z, Jing N, Zhu HH, Dong L, Zhu Y, Dong B, Pan J, Wang Q, Xue W. The ELAVL3/MYCN positive feedback loop provides a therapeutic target for neuroendocrine prostate cancer. Nat Commun. 2023 Nov 28;14(1):7794. https://doi.org/10.1038/s41467-023-43676-3.
53. Kim H, Jang B, Zhang C, Caldwell B, Park DJ, Kong SH, Lee HJ, Yang HK, Goldenring JR, Choi E. Targeting Stem Cells and Dysplastic Features With Dual MEK/ERK and STAT3 Suppression in Gastric Carcinogenesis. Gastroenterology. 2024 Jan;166(1):117-131. https://doi.org/10.1053/j.gastro.2023.09.040.
54. Zhang C, Zhang Z, Zhang S, Wang W, Hu P. Targeting of Wnt/β-Catenin by Anthelmintic Drug Pyrvinium Enhances Sensitivity of Ovarian Cancer Cells to Chemotherapy. Med Sci Monit. 2017 Jan 16;23:266-275. https://doi.org/10.12659/msm.901667.
55. Barbarino M, Cesari D, Intruglio R, Indovina P, Namagerdi A, Bertolino FM, Bottaro M, Rahmani D, Bellan C, Giordano A. Possible repurposing of pyrvinium pamoate for the treatment of mesothelioma: A pre-clinical assessment. J Cell Physiol. 2018 Sep;233(9):7391-7401. https://doi.org/10.1002/jcp.26579.
56. Yang J, Lim JT, Santiago Raj PV, Corona MG, Chen C, Khawaja H, Pan Q, Paine-Murrieta GD, Schnellmann RG, Roe DJ, Gokhale PC, DeCaprio JA, Padi M. Integrative analysis reveals therapeutic potential of pyrvinium pamoate in Merkel cell carcinoma. J Clin Invest. 2025;135(7):e177724. <https://doi.org/10.1172/JCI177724>.
57. Cui L, Zhao J, Liu J. Pyrvinium Sensitizes Clear Cell Renal Cell Carcinoma Response to Chemotherapy Via Casein Kinase 1α-Dependent Inhibition of Wnt/β-Catenin. Am J Med Sci. 2018 Mar;355(3):274-280. https://doi.org/10.1016/j.amjms.2017.11.017.
58. Zheng L, Liu Y, Pan J. Inhibitory effect of pyrvinium pamoate on uveal melanoma cells involves blocking of Wnt/β-catenin pathway. Acta Biochim Biophys Sin (Shanghai). 2017 Oct 1;49(10):890-898. https://doi.org/10.1093/abbs/gmx089.
59. Yi Y, Woo YM, Hwang KH, Kim HS, Lee SH. Niclosamide and Pyrvinium Are Both Potential Therapeutics for Osteosarcoma, Inhibiting Wnt-Axin2-Snail Cascade. Cancers (Basel). 2021 Sep 15;13(18):4630. https://doi.org/10.3390/cancers13184630.
60. Fu YH, Tseng CY, Lu JW, Lu WH, Lan PQ, Chen CY, Ou DL, Lin LI. Deciphering the Role of Pyrvinium Pamoate in the Generation of Integrated Stress Response and Modulation of Mitochondrial Function in Myeloid Leukemia Cells through Transcriptome Analysis. Biomedicines. 2021 Dec 9;9(12):1869. https://doi.org/10.3390/biomedicines9121869.
61. Polosukhina D, Love HD, Moses HL, Lee E, Zent R, Clark PE. Pharmacologic Inhibition of β-Catenin With Pyrvinium Inhibits Murine and Human Models of Wilms Tumor. Oncol Res. 2017 Nov 2;25(9):1653-1664. https://doi.org/10.3727/096504017X14992942781895.
62. Xu H, Wang Z, Xu L, Mo G, Duan G, Wang Y, Sun Z, Chen H. Targeting the eIF4E/β-catenin axis sensitizes cervical carcinoma squamous cells to chemotherapy. Am J Transl Res. 2017 Mar 15;9(3):1203-1212.
63. Majumder M, Janakiraman H, Chakraborty P, Vijayakumar A, Mayhue S, Yu H, Dincman T, Martin R, O'Quinn E, Mehrotra S, Palanisamy V. RNA-binding protein HuR reprograms immune T cells and promotes oral squamous cell carcinoma. Oral Oncol Rep. 2024 Jun;10:100296. https://doi.org/10.1016/j.oor.2024.100296.
64. Sugimoto K, Hayakawa F, Shimada S, Morishita T, Shimada K, Katakai T, Tomita A, Kiyoi H, Naoe T. Discovery of a drug targeting microenvironmental support for lymphoma cells by screening using patient-derived xenograft cells. Sci Rep. 2015 Aug 17;5:13054. https://doi.org/10.1038/srep13054.
65. Pan M, Hu T, Lyu J, Yin Y, Sun J, Wang Q, Xu L, Hu H, Wang C. CSNK1A1/CK1α suppresses autoimmunity by restraining the CGAS-STING1 signaling. Autophagy. 2024 Feb;20(2):311-328. https://doi.org/10.1080/15548627.2023.2256135.
66. Rodriguez-Blanco J, Li B, Long J, Shen C, Yang F, Orton D, Collins S, Kasahara N, Ayad NG, McCrea HJ, Roussel MF, Weiss WA, Capobianco AJ, Robbins DJ. A CK1α Activator Penetrates the Brain and Shows Efficacy Against Drug-resistant Metastatic Medulloblastoma. Clin Cancer Res. 2019 Feb 15;25(4):1379-1388. https://doi.org/10.1158/1078-0432.CCR-18-1319.
67. Li B, Orton D, Neitzel LR, Astudillo L, Shen C, Long J, Chen X, Kirkbride KC, Doundoulakis T, Guerra ML, Zaias J, Fei DL, Rodriguez-Blanco J, Thorne C, Wang Z, Jin K, Nguyen DM, Sands LR, Marchetti F, Abreu MT, Cobb MH, Capobianco AJ, Lee E, Robbins DJ. Differential abundance of CK1α provides selectivity for pharmacological CK1α activators to target WNT-dependent tumors. Sci Signal. 2017 Jun 27;10(485):eaak9916. https://doi.org/10.1126/scisignal.aak9916.
68. Le PN, McDermott JD, Jimeno A. Targeting the Wnt pathway in human cancers: therapeutic targeting with a focus on OMP-54F28. Pharmacol Ther. 2015 Feb;146:1-11. https://doi.org/10.1016/j.pharmthera.2014.08.005.
69. Gurney A, Axelrod F, Bond CJ, Cain J, Chartier C, Donigan L, Fischer M, Chaudhari A, Ji M, Kapoun AM, Lam A, Lazetic S, Ma S, Mitra S, Park IK, Pickell K, Sato A, Satyal S, Stroud M, Tran H, Yen WC, Lewicki J, Hoey T. Wnt pathway inhibition via the targeting of Frizzled receptors results in decreased growth and tumorigenicity of human tumors. Proc Natl Acad Sci U S A. 2012 Jul 17;109(29):11717-22. https://doi.org/10.1073/pnas.1120068109.
70. Kelsey R. Prostate cancer: Foxy-5 in prostate cancer model. Nat Rev Urol. 2017 Nov;14(11):638. https://doi.org/10.1038/nrurol.2017.160.
71. Säfholm A, Tuomela J, Rosenkvist J, Dejmek J, Härkönen P, Andersson T. The Wnt-5a-derived hexapeptide Foxy-5 inhibits breast cancer metastasis in vivo by targeting cell motility. Clin Cancer Res. 2008 Oct 15;14(20):6556-63. https://doi.org/10.1158/1078-0432.CCR-08-0711.
72. Canesin G, Evans-Axelsson S, Hellsten R, Krzyzanowska A, Prasad CP, Bjartell A, Andersson T. Treatment with the WNT5A-mimicking peptide Foxy-5 effectively reduces the metastatic spread of WNT5A-low prostate cancer cells in an orthotopic mouse model. PLoS One. 2017 Sep 8;12(9):e0184418. https://doi.org/10.1371/journal.pone.0184418.
73. Bueno MLP, Saad STO, Roversi FM. The antitumor effects of WNT5A against hematological malignancies. J Cell Commun Signal. 2023 Dec;17(4):1487-1499. https://doi.org/10.1007/s12079-023-00773-8.
74. Mehdawi LM, Prasad CP, Ehrnström R, Andersson T, Sjölander A. Non-canonical WNT5A signaling up-regulates the expression of the tumor suppressor 15-PGDH and induces differentiation of colon cancer cells. Mol Oncol. 2016 Nov;10(9):1415-1429. https://doi.org/10.1016/j.molonc.2016.07.011.
75. Sudo H, Tsuji AB, Sugyo A, Harada Y, Nagayama S, Katagiri T, Nakamura Y, Higashi T. FZD10-targeted α-radioimmunotherapy with 225 Ac-labeled OTSA101 achieves complete remission in a synovial sarcoma model. Cancer Sci. 2022 Feb;113(2):721-732. https://doi.org/10.1111/cas.15235.
76. Cai MY, Yin P, Wang ZW, Huang YZ, Wang YX, Wu KF, Zhang X, Shi L, Wei JF, Ding Q. Dual regulation of FZD1/7 by IGF2BP3 enhances stem-like properties and carboplatin resistance in triple-negative breast cancer. Cancer Lett. 2025 Nov 1;632:217944. https://doi.org/10.1016/j.canlet.2025.217944.
77. Urushibara S, Tsubota T, Asai R, Azumi J, Ashida K, Fujiwara Y, Shiota G. WNT/β-Catenin Signaling Inhibitor IC-2 Suppresses Sphere Formation and Sensitizes Colorectal Cancer Cells to 5-Fluorouracil. Anticancer Res. 2017 Aug;37(8):4085-4091. https://doi.org/10.21873/anticanres.11795.
78. Seto K, Sakabe T, Itaba N, Azumi J, Oka H, Morimoto M, Umekita Y, Shiota G. A Novel Small-molecule WNT Inhibitor, IC-2, Has the Potential to Suppress Liver Cancer Stem Cells. Anticancer Res. 2017 Jul;37(7):3569-3579. https://doi.org/10.21873/anticanres.11727.
79. Chen Y, Chen L, Yu J, Ghia EM, Choi MY, Zhang L, Zhang S, Sanchez-Lopez E, Widhopf GF 2nd, Messer K, Rassenti LZ, Jamieson C, Kipps TJ. Cirmtuzumab blocks Wnt5a/ROR1 stimulation of NF-κB to repress autocrine STAT3 activation in chronic lymphocytic leukemia. Blood. 2019 Sep 26;134(13):1084-1094. https://doi.org/10.1182/blood.2019001366.
80. Yu J, Chen Y, Chen L, Zhang L, Rassenti LZ, Widhopf GF 2nd, Kipps TJ. Cirmtuzumab inhibits ibrutinib-resistant, Wnt5a-induced Rac1 activation and proliferation in mantle cell lymphoma. Oncotarget. 2018 May 15;9(37):24731-24736. https://doi.org/10.18632/oncotarget.25340.
81. Zhang S, Zhang H, Ghia EM, Huang J, Wu L, Zhang J, Lam S, Lei Y, He J, Cui B, Widhopf GF 2nd, Yu J, Schwab R, Messer K, Jiang W, Parker BA, Carson DA, Kipps TJ. Inhibition of chemotherapy resistant breast cancer stem cells by a ROR1 specific antibody. Proc Natl Acad Sci U S A. 2019 Jan 22;116(4):1370-1377. https://doi.org/10.1073/pnas.1816262116.
82. Weng, J., Le, C. C., Pan, Y., Perutelli, F., *et al*. Targeting B-Cell Malignancies with Anti-ROR1 CAR T-Cell Therapy. Blood (2023) 142 (Supplement 1): 6827. <https://doi.org/10.1182/blood-2023-180028>
83. Liu D, Vandenberg CJ, Sini P, Waldmeier L, Baumgartinger R, Pisarsky L, Petroczi G, Ratnayake G, Scott CL, Ford CE. The antibody-drug conjugate targeting ROR1, NBE-002, is active in high-grade serous ovarian cancer preclinical models. Ther Adv Med Oncol. 2025 Apr 21;17:17588359251332471. https://doi.org/10.1177/17588359251332471.
84. Jiang VC, Liu Y, Jordan A, McIntosh J, Li Y, Che Y, Jessen KA, Lannutti BJ, Wang M. The antibody drug conjugate VLS-101 targeting ROR1 is effective in CAR T-resistant mantle cell lymphoma. J Hematol Oncol. 2021 Aug 28;14(1):132. https://doi.org/10.1186/s13045-021-01143-w.
85. Daneshmanesh AH, Hojjat-Farsangi M, Ghaderi A, Moshfegh A, Hansson L, Schultz J, Vågberg J, Byström S, Olsson E, Olin T, Österborg A, Mellstedt H. A receptor tyrosine kinase ROR1 inhibitor (KAN0439834) induced significant apoptosis of pancreatic cells which was enhanced by erlotinib and ibrutinib. PLoS One. 2018 Jun 1;13(6):e0198038. https://doi.org/10.1371/journal.pone.0198038.
86. Hojjat-Farsangi M, Daneshmanesh AH, Khan AS, Shetye J, Mozaffari F, Kharaziha P, Rathje LS, Kokhaei P, Hansson L, Vågberg J, Byström S, Olsson E, Löfberg C, Norström C, Schultz J, Norin M, Olin T, Österborg A, Mellstedt H, Moshfegh A. First-in-class oral small molecule inhibitor of the tyrosine kinase ROR1 (KAN0439834) induced significant apoptosis of chronic lymphocytic leukemia cells. Leukemia. 2018 Oct;32(10):2291-2295. https://doi.org/10.1038/s41375-018-0113-1.
87. Fujii, N., You, L., Xu, Z., Uematsu, K., Shan, J., He, B., *et al*. (2007). An antagonist of dishevelled protein-protein interaction suppresses β-catenin–dependent tumor cell growth. Cancer research, 67(2), 573-579. https://doi.org/10.1158/0008-5472.CAN-06-2726
88. Zhang K, Song H, Yang P, Dai X, Li Y, Wang L, Du J, Pan K, Zhang T. Silencing dishevelled-1 sensitizes paclitaxel-resistant human ovarian cancer cells via AKT/GSK-3β/β-catenin signalling. Cell Prolif. 2015 Apr;48(2):249-58. https://doi.org/10.1111/cpr.12161.
89. Luo K, Gu X, Liu J, Zeng G, Peng L, Huang H, Jiang M, Yang P, Li M, Yang Y, Wang Y, Peng Q, Zhu L, Zhang K. Inhibition of disheveled-2 resensitizes cisplatin-resistant lung cancer cells through down-regulating Wnt/β-catenin signaling. Exp Cell Res. 2016 Sep 10;347(1):105-113. https://doi.org/10.1016/j.yexcr.2016.07.014.
90. Chen S, Kong W, Shen X, Sinha N, Haag J, Deng B, Zhang H, John C, Sun W, Zhou C, Bae-Jump VL. Sulindac exhibits anti-proliferative and anti-invasive effects and enhances the sensitivity to paclitaxel in ovarian cancer. Front Pharmacol. 2025 Apr 30;16:1520771. https://doi.org/10.3389/fphar.2025.1520771.
91. Li J, Wu G, Xu Y, Li J, Ruan N, Chen Y, Zhang Q, Xia Q. Porcupine Inhibitor LGK974 Downregulates the Wnt Signaling Pathway and Inhibits Clear Cell Renal Cell Carcinoma. Biomed Res Int. 2020 Feb 13;2020:2527643. https://doi.org/10.1155/2020/2527643.
92. Bagheri M, Tabatabae Far MA, Mirzaei H, Ghasemi F. Evaluation of antitumor effects of aspirin and LGK974 drugs on cellular signaling pathways, cell cycle and apoptosis in colorectal cancer cell lines compared to oxaliplatin drug. Fundam Clin Pharmacol. 2020 Feb;34(1):51-64. https://doi.org/10.1111/fcp.12492.
93. Guimaraes PPG, Tan M, Tammela T, Wu K, Chung A, Oberli M, Wang K, Spektor R, Riley RS, Viana CTR, Jacks T, Langer R, Mitchell MJ. Potent in vivo lung cancer Wnt signaling inhibition via cyclodextrin-LGK974 inclusion complexes. J Control Release. 2018 Nov 28;290:75-87. https://doi.org/10.1016/j.jconrel.2018.09.025.
94. Kahlert UD, Suwala AK, Koch K, Natsumeda M, Orr BA, Hayashi M, Maciaczyk J, Eberhart CG. Pharmacologic Wnt Inhibition Reduces Proliferation, Survival, and Clonogenicity of Glioblastoma Cells. J Neuropathol Exp Neurol. 2015 Sep;74(9):889-900. https://doi.org/10.1097/NEN.0000000000000227.
95. Bland T, Wang J, Yin L, Pu T, Li J, Gao J, Lin TP, Gao AC, Wu BJ. WLS-Wnt signaling promotes neuroendocrine prostate cancer. iScience. 2021 Jan 1;24(1):101970. https://doi.org/10.1016/j.isci.2020.101970.
96. Zimmerli D, Cecconi V, Valenta T, Hausmann G, Cantù C, Restivo G, Hafner J, Basler K, van den Broek M. WNT ligands control initiation and progression of human papillomavirus-driven squamous cell carcinoma. Oncogene. 2018 Jul;37(27):3753-3762. https://doi.org/10.1038/s41388-018-0244-x.
97. Cheng Y, Phoon YP, Jin X, Chong SY, Ip JC, Wong BW, Lung ML. Wnt-C59 arrests stemness and suppresses growth of nasopharyngeal carcinoma in mice by inhibiting the Wnt pathway in the tumor microenvironment. Oncotarget. 2015 Jun 10;6(16):14428-39. https://doi.org/10.18632/oncotarget.3982.
98. Proffitt KD, Madan B, Ke Z, Pendharkar V, Ding L, Lee MA, Hannoush RN, Virshup DM. Pharmacological inhibition of the Wnt acyltransferase PORCN prevents growth of WNT-driven mammary cancer. Cancer Res. 2013 Jan 15;73(2):502-7. https://doi.org/10.1158/0008-5472.CAN-12-2258.
99. Wang B, Zou Q, Sun M, Chen J, Wang T, Bai Y, Chen Z, Chen B, Zhou M. Reversion of trichostatin A resistance via inhibition of the Wnt signaling pathway in human pancreatic cancer cells. Oncol Rep. 2014 Nov;32(5):2015-22. https://doi.org/10.3892/or.2014.3476.
100. Goldsberry WN, Meza-Perez S, Londoño AI, Katre AA, Mott BT, Roane BM, Goel N, Wall JA, Cooper SJ, Norian LA, Randall TD, Birrer MJ, Arend RC. Inhibiting WNT Ligand Production for Improved Immune Recognition in the Ovarian Tumor Microenvironment. Cancers (Basel). 2020 Mar 24;12(3):766. https://doi.org/10.3390/cancers12030766.
101. Frewer KA, Sanders AJ, Owen S, Frewer NC, Hargest R, Jiang WG. A role for WISP2 in colorectal cancer cell invasion and motility. Cancer Genomics Proteomics. 2013 Jul-Aug;10(4):187-96.
102. Ben Ghedalia-Peled N, Cohen-Erez I, Rapaport H, Vago R. Aggressiveness of 4T1 breast cancer cells hampered by Wnt production-2 inhibitor nanoparticles: An in vitro study. Int J Pharm. 2021 Mar 1;596:120208. https://doi.org/10.1016/j.ijpharm.2021.120208.
103. Mo ML, Li MR, Chen Z, Liu XW, Sheng Q, Zhou HM. Inhibition of the Wnt palmitoyltransferase porcupine suppresses cell growth and downregulates the Wnt/β-catenin pathway in gastric cancer. Oncol Lett. 2013 May;5(5):1719-1723. https://doi.org/10.3892/ol.2013.1256.
104. Kleszcz R, Szymańska A, Krajka-Kuźniak V, Baer-Dubowska W, Paluszczak J. Inhibition of CBP/β-catenin and porcupine attenuates Wnt signaling and induces apoptosis in head and neck carcinoma cells. Cell Oncol (Dordr). 2019 Aug;42(4):505-520. https://doi.org/10.1007/s13402-019-00440-4.
105. Lin R, Feng J, Dong S, Pan R, Zhuang H, Ding Z. Regulation of autophagy of prostate cancer cells by β-catenin signaling. Cell Physiol Biochem. 2015;35(3):926-32. https://doi.org/10.1159/000369749.
106. Gao L, Meng J, Zhang M, Fan S, Gao S, Wang X, Liang C. Expression and Prognostic Values of the Roof Plate-Specific Spondin Family in Bladder Cancer. DNA Cell Biol. 2020 Jun;39(6):1072-1089. https://doi.org/10.1089/dna.2019.5224.
107. Chua K, Sim AYL, Yeo EYM, Bin Masroni MS, Naw WW, Leong SM, Lee KW, Lim HJ, Virshup DM, Lee VKM. ETC-159, an Upstream Wnt inhibitor, Induces Tumour Necrosis via Modulation of Angiogenesis in Osteosarcoma. Int J Mol Sci. 2023 Mar 1;24(5):4759. https://doi.org/10.3390/ijms24054759.
108. Madan B, Ke Z, Harmston N, Ho SY, Frois AO, Alam J, Jeyaraj DA, Pendharkar V, Ghosh K, Virshup IH, Manoharan V, Ong EH, Sangthongpitag K, Hill J, Petretto E, Keller TH, Lee MA, Matter A, Virshup DM. Wnt addiction of genetically defined cancers reversed by PORCN inhibition. Oncogene. 2016 Apr 28;35(17):2197-207. https://doi.org/10.1038/onc.2015.280.
109. Hawkins HJ, Yacob BW, Brown ME, Goldstein BR, Arcaroli JJ, Bagby SM, Hartman SJ, Macbeth M, Goodspeed A, Danhorn T, Lentz RW, Lieu CH, Leal AD, Messersmith WA, Dempsey PJ, Pitts TM. Examination of Wnt signaling as a therapeutic target for pancreatic ductal adenocarcinoma (PDAC) using a pancreatic tumor organoid library (PTOL). PLoS One. 2024 Apr 10;19(4):e0298808. https://doi.org/10.1371/journal.pone.0298808.
110. Phillips C, Bhamra I, Eagle C, Flanagan E, Armer R, Jones CD, Bingham M, Calcraft P, Edmenson Cook A, Thompson B, Woodcock SA. The Wnt Pathway Inhibitor RXC004 Blocks Tumor Growth and Reverses Immune Evasion in Wnt Ligand-dependent Cancer Models. Cancer Res Commun. 2022 Sep 2;2(9):914-928. https://doi.org/10.1158/2767-9764.CRC-21-0095.
111. Cheng D, Liu J, Han D, Zhang G, Gao W, Hsieh MH, Ng N, Kasibhatla S, Tompkins C, Li J, Steffy A, Sun F, Li C, Seidel HM, Harris JL, Pan S. Discovery of Pyridinyl Acetamide Derivatives as Potent, Selective, and Orally Bioavailable Porcupine Inhibitors. ACS Med Chem Lett. 2016 May 10;7(7):676-80. https://doi.org/10.1021/acsmedchemlett.6b00038.
112. Wang W, Cho U, Yoo A, Jung CL, Kim B, Kim H, Lee J, Jo H, Han Y, Song MH, Lee JO, Kim SI, Lee M, Ku JL, Lee C, Song YS. Wnt/β-Catenin Inhibition by CWP232291 as a Novel Therapeutic Strategy in Ovarian Cancer. Front Oncol. 2022 May 12;12:852260. https://doi.org/10.3389/fonc.2022.852260.
113. Pak S, Park S, Kim Y, Park JH, Park CH, Lee KJ, Kim CS, Ahn H. The small molecule WNT/β-catenin inhibitor CWP232291 blocks the growth of castration-resistant prostate cancer by activating the endoplasmic reticulum stress pathway. J Exp Clin Cancer Res. 2019 Aug 6;38(1):342. https://doi.org/10.1186/s13046-019-1342-5.
114. Park JW, Seo MJ, Cho KS, Kook MC, Jeong JM, Roh SG, Cho SY, Cheon JH, Kim HK. Smad4 and p53 synergize in suppressing autochthonous intestinal cancer. Cancer Med. 2022 May;11(9):1925-1936. https://doi.org/10.1002/cam4.4533.
115. Humtsoe JO, Keam B, Kim HS, Jones L, Leonard B, Pothukuchi A, Honjo S, van Zante A, Lee S, Ha PK. The Small Molecule Inhibitor of the Wnt-β-Catenin Pathway, CWP232291, Inhibits Tumor Growth in Preclinical Models of Salivary Gland Adenoid Cystic Carcinoma. Head Neck. 2025 Jul 17; 47(12): 3321–3330. https://doi.org/10.1002/hed.28249.
116. Ryu WJ, Han G, Lee SH, Choi KY. Suppression of Wnt/β-catenin and RAS/ERK pathways provides a therapeutic strategy for gemcitabine-resistant pancreatic cancer. Biochem Biophys Res Commun. 2021 Apr 16;549:40-46. https://doi.org/10.1016/j.bbrc.2021.02.076.
117. Park J, Cho YH, Shin WJ, Lee SK, Lee J, Kim T, Cha PH, Yang JS, Cho J, Min DS, Han G, Lee HY, Choi KY. A Ras destabilizer KYA1797K overcomes the resistance of EGFR tyrosine kinase inhibitor in KRAS-mutated non-small cell lung cancer. Sci Rep. 2019 Jan 24;9(1):648. https://doi.org/10.1038/s41598-018-37059-8.
118. Ryu WJ, Lee JD, Park JC, Cha PH, Cho YH, Kim JY, Sohn JH, Paik S, Choi KY. Destabilization of β-catenin and RAS by targeting the Wnt/β-catenin pathway as a potential treatment for triple-negative breast cancer. Exp Mol Med. 2020 May;52(5):832-842. https://doi.org/10.1038/s12276-020-0440-y.
119. Cho YH, Ro EJ, Yoon JS, Kwak DK, Cho J, Kang DW, Lee HY, Choi KY. Small molecule-induced simultaneous destabilization of β-catenin and RAS is an effective molecular strategy to suppress stemness of colorectal cancer cells. Cell Commun Signal. 2020 Mar 6;18(1):38. https://doi.org/10.1186/s12964-020-0519-z.
120. Ghamsari, L., Scuoppo, C., Gallagher, E., Leong, S., Koester, M., Ramirez, R., *et al*. (2023). Abstract B149: Anti-tumor and immunostimulatory properties of ST316, a peptide antagonist of beta-catenin for treatment of cancers with aberrant Wnt pathway activity. Molecular Cancer Therapeutics, 22(12_Supplement), B149-B149. <https://doi.org/10.1158/1535-7163.TARG-23-B149>.
121. Su H, Jin X, Zhang X, Zhao L, Lin B, Li L, Fei Z, Shen L, Fang Y, Pan H, Xie C. FH535 increases the radiosensitivity and reverses epithelial-to-mesenchymal transition of radioresistant esophageal cancer cell line KYSE-150R. J Transl Med. 2015 Mar 31;13:104. https://doi.org/10.1186/s12967-015-0464-6.
122. Suknuntha K, Thita T, Togarrati PP, Ratanachamnong P, Wongtrakoongate P, Srihirun S, Slukvin I, Hongeng S. Wnt signaling inhibitor FH535 selectively inhibits cell proliferation and potentiates imatinib-induced apoptosis in myeloid leukemia cell lines. Int J Hematol. 2017 Feb;105(2):196-205. https://doi.org/10.1007/s12185-016-2116-x.
123. Avşar EN, Çetin İ. Combined use of WNT signal pathway inhibitor FH535 and docetaxel causes mitotic catastrophism and antiproliferative effect in non-small cell lung cancer. Cell Mol Biol (Noisy-le-grand). 2025 Feb 20;71(2):61-66. https://doi.org/10.14715/cmb/2025.71.2.9.
124. Liu L, Zhi Q, Shen M, Gong FR, Zhou BP, Lian L, Shen B, Chen K, Duan W, Wu MY, Tao M, Li W. FH535, a β-catenin pathway inhibitor, represses pancreatic cancer xenograft growth and angiogenesis. Oncotarget. 2016 Jul 26;7(30):47145-47162. https://doi.org/10.18632/oncotarget.9975.
125. Iida J, Dorchak J, Lehman JR, Clancy R, Luo C, Chen Y, Somiari S, Ellsworth RE, Hu H, Mural RJ, Shriver CD. FH535 inhibited migration and growth of breast cancer cells. PLoS One. 2012;7(9):e44418. https://doi.org/10.1371/journal.pone.0044418.
126. Tu X, Hong D, Jiang Y, Lou Z, Wang K, Jiang Y, Jin L. FH535 inhibits proliferation and migration of colorectal cancer cells by regulating CyclinA2 and Claudin1 gene expression. Gene. 2019 Mar 30;690:48-56. https://doi.org/10.1016/j.gene.2018.12.008.
127. Liu X, Du P, Han L, Zhang A, Jiang K, Zhang Q. Effects of miR-200a and FH535 combined with taxol on proliferation and invasion of gastric cancer. Pathol Res Pract. 2018 Mar;214(3):442-449. https://doi.org/10.1016/j.prp.2017.12.004.
128. Kleszcz R, Frąckowiak M, Dorna D, Paluszczak J. Combinations of PRI-724 Wnt/β-Catenin Pathway Inhibitor with Vismodegib, Erlotinib, or HS-173 Synergistically Inhibit Head and Neck Squamous Cancer Cells. Int J Mol Sci. 2023 Jun 21;24(13):10448. https://doi.org/10.3390/ijms241310448.
129. He Y, Liu D, Ling A, Han Z, Cui J, Cheng J, Feng Y, Liu W, Gong W, Xia Y, Chen L, Luo Y, Wang Y, Tian X, Wu Q, Chen L, Guo H. Inhibition of Wnt/β-catenin increases anti-tumor activity by synergizing with sorafenib in hepatocellular carcinoma. Cell Death Dis. 2025 Jul 1;16(1):466. https://doi.org/10.1038/s41419-025-07789-5.
130. Nasser F, Moussa N, Helmy MW, Haroun M. Dual targeting of Notch and Wnt/β-catenin pathways: Potential approach in triple-negative breast cancer treatment. Naunyn Schmiedebergs Arch Pharmacol. 2021 Mar;394(3):481-490. https://doi.org/10.1007/s00210-020-01988-x.
131. Brkic FF, Stoiber S, Maier T, Gurnhofer E, Kenner L, Heiduschka G, Kadletz-Wanke L. Targeting Wnt/Beta-Catenin Signaling in HPV-Positive Head and Neck Squamous Cell Carcinoma. Pharmaceuticals (Basel). 2022 Mar 20;15(3):378. https://doi.org/10.3390/ph15030378.
132. Zhang Z, Cheng L, Li J, Farah E, Atallah NM, Pascuzzi PE, Gupta S, Liu X. Inhibition of the Wnt/β-Catenin Pathway Overcomes Resistance to Enzalutamide in Castration-Resistant Prostate Cancer. Cancer Res. 2018 Jun 15;78(12):3147-3162. https://doi.org/10.1158/0008-5472.CAN-17-3006.
133. Evangelisti C, Chiarini F, Cappellini A, Paganelli F, Fini M, Santi S, Martelli AM, Neri LM, Evangelisti C. Targeting Wnt/β-catenin and PI3K/Akt/mTOR pathways in T-cell acute lymphoblastic leukemia. J Cell Physiol. 2020 Jun;235(6):5413-5428. https://doi.org/10.1002/jcp.29429.
134. Feng D, Yan K, Liang H, Liang J, Wang W, Yu H, Zhou Y, Zhao W, Dong Z, Ling B. CBP-mediated Wnt3a/β-catenin signaling promotes cervical oncogenesis initiated by Piwil2. Neoplasia. 2021 Jan;23(1):1-11. https://doi.org/10.1016/j.neo.2020.10.013.
135. Liu Y, Chen H, Zheng P, Zheng Y, Luo Q, Xie G, Ma Y, Shen L. ICG-001 suppresses growth of gastric cancer cells and reduces chemoresistance of cancer stem cell-like population. J Exp Clin Cancer Res. 2017 Sep 11;36(1):125. https://doi.org/10.1186/s13046-017-0595-0.
136. Kaochar S, Dong J, Torres M, Rajapakshe K, Nikolos F, Davis CM, Ehli EA, Coarfa C, Mitsiades N, Poulaki V. ICG-001 Exerts Potent Anticancer Activity Against Uveal Melanoma Cells. Invest Ophthalmol Vis Sci. 2018 Jan 1;59(1):132-143. https://doi.org/10.1167/iovs.17-22454.
137. Cui Y, Wu X, Lin C, Zhang X, Ye L, Ren L, Chen M, Yang M, Li Y, Li M, Li J, Guan J, Song L. AKIP1 promotes early recurrence of hepatocellular carcinoma through activating the Wnt/β-catenin/CBP signaling pathway. Oncogene. 2019 Jul;38(27):5516-5529. https://doi.org/10.1038/s41388-019-0807-5.
138. Han Y, Li C, Liu S, Gao J, He Y, Xiao H, Chen Q, Zheng Y, Chen H, Zhu X. Combined targeting of Hedgehog/GLI1 and Wnt/β-catenin pathways in mantle cell lymphoma. Hematol Oncol. 2024 Sep;42(5):e3305. https://doi.org/10.1002/hon.3305.
139. Ham A, Cho MH, Won HS, Jo J, Lee KE. β‑catenin blockers enhance the effect of CDK4/6 inhibitors on stemness and proliferation suppression in endocrine‑resistant breast cancer cells. Oncol Rep. 2022 Jul;48(1):130. https://doi.org/10.3892/or.2022.8341.
140. Deng J, Hua L, Han T, Tian M, Wang D, Tang H, Sun S, Chen H, Cheng H, Zhang T, Xie Q, Wan L, Zhu H, Gong Y. The CREB-binding protein inhibitor ICG-001: a promising therapeutic strategy in sporadic meningioma with NF2 mutations. Neurooncol Adv. 2020 Feb 22;2(1):vdz055. https://doi.org/10.1093/noajnl/vdz055.
141. Mesropian A, Gris-Oliver A, Balaseviciute U, Potdar AA, Kimura T, Shen J, Torres-Marcén M, Abril-Fornaguera J, Piqué-Gili M, Camell-Raventos D, Peix J, Fernández-Martínez E, Huguet-Pradell J, Hernández de Sande A, Keraite I, Esteban-Fabró R, Barcena-Varela M, Lindblad KE, Lujambio A, Guccione E, Thung S, Ikeda M, Kudo M, Sia D, Pinyol R, Llovet JM. E7386 enhances lenvatinib's antitumor activity in preclinical models and human hepatocellular carcinoma. Clin Cancer Res. 2025; 31 (23): 5037–5050. https://doi.org/10.1158/1078-0432.CCR-25-0725.
142. Kanda Y, Ohata H, Miyazaki T, Sakai H, Mori Y, Shiokawa D, Yokoi A, Owa T, Ochiai A, Okamoto K. NF-κB suppression synergizes with E7386, an inhibitor of CBP/β-catenin interaction, to block proliferation of patient-derived colon cancer spheroids. Biochem Biophys Res Commun. 2022 Jan 1;586:93-99. https://doi.org/10.1016/j.bbrc.2021.11.063.
143. Reed ER, Jankowski SA, Spinella AJ, Noonan V, Haddad R, Nomoto K, Matsui J, Bais MV, Varelas X, Kukuruzinska MA, Monti S. β-catenin/CBP activation of mTORC1 signaling promotes partial epithelial-mesenchymal states in head and neck cancer. Transl Res. 2023 Oct;260:46-60. https://doi.org/10.1016/j.trsl.2023.05.007.
144. Amado NG, Predes D, Fonseca BF, Cerqueira DM, Reis AH, Dudenhoeffer AC, Borges HL, Mendes FA, Abreu JG. Isoquercitrin suppresses colon cancer cell growth in vitro by targeting the Wnt/β-catenin signaling pathway. J Biol Chem. 2014 Dec 19;289(51):35456-67. https://doi.org/10.1074/jbc.M114.621599.
145. Minke KS, Staib P, Puetter A, Gehrke I, Gandhirajan RK, Schlösser A, Schmitt EK, Hallek M, Kreuzer KA. Small molecule inhibitors of WNT signaling effectively induce apoptosis in acute myeloid leukemia cells. Eur J Haematol. 2009 Mar;82(3):165-75. https://doi.org/10.1111/j.1600-0609.2008.01188.x.
146. Gandhirajan RK, Staib PA, Minke K, Gehrke I, Plickert G, Schlösser A, Schmitt EK, Hallek M, Kreuzer KA. Small molecule inhibitors of Wnt/beta-catenin/lef-1 signaling induces apoptosis in chronic lymphocytic leukemia cells in vitro and in vivo. Neoplasia. 2010 Apr;12(4):326-35. https://doi.org/10.1593/neo.91972.
147. Wei W, Chua MS, Grepper S, So S. Small molecule antagonists of Tcf4/beta-catenin complex inhibit the growth of HCC cells in vitro and in vivo. Int J Cancer. 2010 May 15;126(10):2426-36. https://doi.org/10.1002/ijc.24810.
148. Sogutlu F, Kayabasi C, Ozmen Yelken B, Asik A, Gasimli R, Dogan F, Yilmaz Süslüer S, Biray Avcı C, Gunduz C. The effect of ICRT-3 on Wnt signaling pathway in head and neck cancer. J Cell Biochem. 2019 Jan;120(1):380-395. https://doi.org/10.1002/jcb.27393.
149. Gonsalves FC, Klein K, Carson BB, Katz S, Ekas LA, Evans S, Nagourney R, Cardozo T, Brown AM, DasGupta R. An RNAi-based chemical genetic screen identifies three small-molecule inhibitors of the Wnt/wingless signaling pathway. Proc Natl Acad Sci U S A. 2011 Apr 12;108(15):5954-63. https://doi.org/10.1073/pnas.1017496108.
150. Fang L, Zhu Q, Neuenschwander M, Specker E, Wulf-Goldenberg A, Weis WI, von Kries JP, Birchmeier W. A Small-Molecule Antagonist of the β-Catenin/TCF4 Interaction Blocks the Self-Renewal of Cancer Stem Cells and Suppresses Tumorigenesis. Cancer Res. 2016 Feb 15;76(4):891-901. https://doi.org/10.1158/0008-5472.CAN-15-1519.
151. Zhao Q, Wu T, Tang C, Li J, Wu M, Wu J, Wang Z, Zhu Y, Xu H, Li X. Biomimetic nanocrystals co-deliver paclitaxel and small-molecule LF3 for ferroptosis-combined chemotherapy for gastric cancer. Colloids Surf B Biointerfaces. 2025 Jul;251:114586. https://doi.org/10.1016/j.colsurfb.2025.114586.
152. Jin S, Wu X. Aspirin inhibits colon cancer cell line migration through regulating epithelial-mesenchymal transition via Wnt signaling. Oncol Lett. 2019 May;17(5):4675-4682. https://doi.org/10.3892/ol.2019.10089.
153. Huang C, Chen Y, Liu H, Yang J, Song X, Zhao J, He N, Zhou CJ, Wang Y, Huang C, Dong Q. Celecoxib targets breast cancer stem cells by inhibiting the synthesis of prostaglandin E2 and down-regulating the Wnt pathway activity. Oncotarget. 2017 Dec 14;8(70):115254-115269. https://doi.org/10.18632/oncotarget.23250.
154. Deng Y, Su Q, Mo J, Fu X, Zhang Y, Lin EH. Celecoxib downregulates CD133 expression through inhibition of the Wnt signaling pathway in colon cancer cells. Cancer Invest. 2013 Feb;31(2):97-102. https://doi.org/10.3109/07357907.2012.754458.
155. Rybarczyk A, Majchrzak-Celińska A, Piwowarczyk L, Krajka-Kuźniak V. The Anti-Glioblastoma Effects of Novel Liposomal Formulations Loaded with Cannabidiol, Celecoxib, and 2,5-Dimethylcelecoxib. Pharmaceutics. 2025 Aug 8;17(8):1031. https://doi.org/10.3390/pharmaceutics17081031.
