# Supplementary material for: The Wnt signaling networks in higher-grade meningiomas: from molecular pathogenesis to therapeutic innovation
Source: Cancer Cell Int. 2026 Jul 24;26:345. doi: 10.1186/s12935-026-04393-1 (PMC13685165; doi:10.1186/s12935-026-04393-1)
Supplement: Supplementary file 4 [file 12935_2026_4393_MOESM4_ESM.docx]

**Supplementary Table 1**. Reported genetic, epigenetic, transcript-level, and miRNA-associated alterations involving Wnt signaling and Wnt-associated regulatory/crosstalk molecules across cancer types. The table summarizes alterations reported in core pathway components, receptors, ligands, transcriptional mediators, pathway inhibitors, and functionally connected upstream or downstream regulators. Listed events include mutations, truncations, deletions, loss of heterozygosity, promoter methylation, chromatin-associated changes, gene fusions, splice alterations, and miRNA-associated regulation.

| **Cancer Type** | **Affected Gene(s)** | **Observed genetic, epigenetic, transcript-level, or miRNA-associated alteration/regulation** | **Reference** |
| --- | --- | --- | --- |
| ACC | *CTNNB1* (β-catenin) | Missense in N-terminal Ser/Thr phosphorylation sites preventing degradation | [1, 2] |
| ACC | *ZNRF3* | Missense / truncating mutations; LOH; homozygous deletion | [1, 2] |
| Bladder cancer | *CTNNB1* | miR-1826 | [3] |
| Bladder cancer | *FZD4* | miR-493 | [3] |
| Breast cancer | *APC* | miR-142 | [3] |
| Breast cancer | *APC* | miR-1229 | [3] |
| Breast cancer | *AXIN2* | miR-34 | [3] |
| Breast cancer | *CTNNB1* | miR-200c | [3] |
| Breast cancer | *CTNNB1* | miR-141 | [3] |
| Breast cancer | *CTNNB1* | miR-340 | [3] |
| Breast cancer | *DKK1* | miR-372/373 | [3] |
| Breast cancer | *DKK1* | Promoter methylation | [4] |
| Breast cancer | *DKK3* | Promoter hypermethylation | [5] |
| Breast cancer | *DKK2* | miR-218 | [3] |
| Breast cancer | *FBXW7* | Rare mutation and/or deletion | [6] |
| Breast cancer | *FZD7* | miR-1 | [3] |
| Breast cancer | *FZD8* | miR-100 | [3] |
| Breast cancer | *GSK3β* | miR-1229 | [3] |
| Breast cancer | *LATS1/2* | Promoter hypermethylation | [7] |
| Breast cancer | *LEF1* | miR-34 | [3] |
| Breast cancer | *SFRP2* | miR-218 | [3] |
| Breast cancer | *SFRP1* | Promoter hypermethylation | [4] |
| Breast cancer | *SFRP2* | Promoter hypermethylation | [4] |
| Breast cancer | *SOST* | miR-218 | [3] |
| Breast cancer | *TCF1* | miR-34 | [3] |
| Breast cancer | *TCF7* | miR-34 | [3] |
| Breast cancer | *WIF1* | miR-374a | [3] |
| Breast cancer | *WIF1* | miR-29 | [3] |
| Breast cancer | *WIF1* | Promoter hypermethylation | [5, 8] |
| Breast cancer | *WNT5A* | miR-374a | [3] |
| CLL | *DKK1* | Promoter hypermethylation | [9] |
| Colon cancer | *CTNNB1* | miR-101 | [3] |
| Colon cancer | *CTNNB1* | miR-320 | [3] |
| Colon cancer | *DKK2* | miR-21 | [3] |
| Colon cancer | *FZD5* | miR-23a/b | [3] |
| Colon cancer | *FZD7* | miR-23a/b | [3] |
| CRC | *APC* | Truncation; frameshift / nonsense mutations leading to truncated APC proteins; LOH | [1, 2] |
| CRC | *APC* | Promoter hypermethylation | [8] |
| CRC | *AXIN1* | Truncation / missense | [1, 2] |
| CRC | *AXIN2* | Truncation / missense | [1, 2] |
| CRC | *AXIN2* | miR-34 | [3] |
| CRC | *AXIN2* | Promoter hypermethylation | [10, 11] |
| CRC | *CTNNB1* (β-catenin) | Missense / deletion / truncation; phosphorylation-site mutations preventing degradation | [1, 2] |
| CRC | *DKK1* | miR-372/373 | [3] |
| CRC | *DKK1* | Promoter hypermethylation | [12] |
| CRC | *FBXW7* | Mutation | [13] |
| CRC | *GSK3β* | miR-224 | [3] |
| CRC | *LATS1* | Promoter hypermethylation | [14] |
| CRC | *LEF1* | miR-34 | [3] |
| CRC | *LGR5* | Single nucleotide polymorphisms / allelic variation | [15] |
| CRC | *LGR5* | Promoter hypermethylation | [16] |
| CRC | *RNF43* | Mutation; missense / truncating mutations (frameshift / nonsense); LOH; homozygous deletion | [1, 2] |
| CRC | *RSPO2* | Chromosome rearrangement → EIF3E–RSPO2 gene fusion | [17] |
| CRC | *RSPO3* | Chromosome rearrangement → PTPRK–RSPO3 gene fusion | [17] |
| CRC | *SFRP1* | Promoter hypermethylation | [18] |
| CRC | *SFRP2* | miR-224 | [3] |
| CRC | *SFRP2* | Promoter hypermethylation | [18] |
| CRC | *SFRP4* | Promoter hypermethylation | [18] |
| CRC | *SFRP5* | Promoter hypermethylation | [18] |
| CRC | *TCF1* | miR-34 | [3] |
| CRC | *TCF7* | miR-34 | [3] |
| CRC | *TCF7L2 (TCF4)* | Fusion with *VTI1A* gene (*VTI1A–TCF7L2* fusion ) | [19] |
| CRC | *WIF1* | Promoter hypermethylation | [20] |
| CRC | *WNT2* | Loss of PRC2/EZH2 promoter occupancy; WNT2 derepression | [21] |
| CRC | *WNT5A* | Promoter hypermethylation | [22] |
| CRC | *WNT9A* | Promoter hypermethylation | [23] |
| CRC | *WNT10B* | Promoter hypermethylation | [24] |
| CRC | *ZNRF3* | Missense / truncating mutations (frameshift / nonsense); LOH; homozygous deletion | [1, 2] |
| CRC | *ZNRF3* | miR-93 | [3] |
| Endometrial cancer | *WNT1* | miR-148a | [3] |
| Endometrial carcinoma (EC) | *APC* | Truncating / missense; LOH | [1, 2] |
| Endometrial carcinoma (EC) | *AXIN2* | Low–moderate mutation frequency | [1, 2] |
| Endometrial carcinoma (EC) | *CTNNB1* (β-catenin) | Missense | [1, 2] |
| Endometrial carcinoma (EC) | *RNF43* | Frameshift mutations | [8, 25] |
| Endometrial carcinoma (EC) | *ZNRF3* | Occasional mutation | [1, 2] |
| Esophageal cancer | *CTNNB1* | miR-141 | [3] |
| Esophageal cancer | *RSPO2* | Recurrent EIF3E–RSPO2 gene fusion | [1, 2] |
| Gastric cancer | *APC* | Truncation | [1, 2] |
| Gastric cancer | *APC* | miR-27 | [3] |
| Gastric cancer | *AXIN1* | Truncation / missense | [1, 2] |
| Gastric cancer | *AXIN2* | Truncation / missense | [1, 2] |
| Gastric cancer | *AXIN2* | miR-544a | [3] |
| Gastric cancer | *CTNNB1* | miR-200a | [3] |
| Gastric cancer | *DKK1* | miR-493 | [3] |
| Gastric cancer | *FZD7* | miR-27b | [3] |
| Gastric cancer | *FZD7* | miR-199a | [3] |
| Gastric cancer | *RNF43* | Recurrent mutation / driver mutation | [26, 27] |
| Gastric cancer | *WNT1* | miR-22 | [3] |
| Gastric cancer | *WNT1* | miR-200b | [3] |
| Glioblastoma | *LEF1* | miR-218 | [3] |
| Glioblastoma | *LRP6* | miR-513c | [3] |
| Glioma | *APC* | miR-155 | [3] |
| Glioma | *LEF1* | miR-218 | [3] |
| Glioma | *SFRP1* | miR-328 | [3] |
| Glioma | *TCF7* | LOC728196 upregulation/LOC728196/miR-513c axis | [28] |
| Glioma | *WIF1* | miR-603 | [3] |
| Glioma | *WNT1* | miR-122 | [3] |
| Glioma | *WNT1* | miR-200b | [3] |
| HCC | *APC* | miR-155 | [3] |
| HCC | *APC* | miR-106b | [3] |
| HCC | *AXIN1* | Truncation / missense | [1, 2] |
| HCC | *AXIN2* | miR-34 | [3] |
| HCC | *CTNNB1* | miR-200a | [3] |
| HCC | *CTNNB1* | miR-33a | [3] |
| HCC | *CTNNB1* | miR-214 | [3] |
| HCC | *CTNNB1* (β-catenin) | Missense / in-frame deletion | [1, 2] |
| HCC | *FZD7* | miR-199a | [3] |
| HCC | *LEF1* | miR-612 | [3] |
| HCC | *LEF1* | miR-34 | [3] |
| HCC | *LRP6* | miR-610 | [3] |
| HCC | *SFRP2* | Promoter hypermethylation | [29] |
| HCC | *TCF7L2 (TCF4)* | miR-139 | [3] |
| HCC | *TCF1* | miR-34 | [3] |
| HCC | *TCF7* | miR-34 | [3] |
| HCC | *WNT1* | miR-122 | [3] |
| HCC | *WNT1* | miR-148a | [3] |
| Head and Neck Cancer (HNC) | *RSPO2* | Recurrent EIF3E–RSPO2 gene fusion | [1, 2] |
| HNSCC | *LATS1/2* | Promoter hypermethylation | [30] |
| HNSCC | *MST1/2* | Promoter hypermethylation | [30] |
| Leukemia | *CREBBP (CBP)* | Truncation / missense | [31] |
| Leukemia | *GSK3β* | Missplicing / in-frame deletion | [32] |
| Liposarcoma | *APC* | miR-155 | [3] |
| Lung cancer | *AXIN2* | miR-34 | [3] |
| Lung cancer | *CTNNB1* | miR-548b | [3] |
| Lung cancer | *FZD2* | miR-203 | [3] |
| Lung cancer | *GSK3β* | miR-1290 | [3] |
| Lung cancer | *LEF1* | miR-34 | [3] |
| Lung cancer | *RSPO2* | Recurrent EIF3E–RSPO2 gene fusion | [1, 2] |
| Lung cancer | *TCF1* | miR-34 | [3] |
| Lung cancer | *TCF7* | miR-34 | [3] |
| Lung cancer | *WIF1* | miR-29 | [3] |
| Lung cancer | *WNT5A* | miR-487b | [3] |
| Lung cancer | *ZNRF3* | miR-93 | [3] |
| Medulloblastoma | *CTNNB1* (β-catenin) | Missense / in-frame deletion | [1, 2] |
| Meningioma | *APC* | LOH | [33] |
| Meningioma | *AXIN1* | LOH, MSI | [34] |
| Meningioma | *CTNNB1* | miR-200a | [35] |
| Meningioma | *DVL1* (Dishevelled-1) | Microsatellite instability; LOH | [36] |
| Meningioma | *SFRP1* | Promoter hypermethylation | [37] |
| Mesothelioma | *MST1* | Promoter hypermethylation | [38] |
| Nasopharyngeal carcinoma | *WNT2B* | miR-185-3p | [3] |
| Nasopharyngeal carcinoma | *WNT2B* | miR-324-3p | [3] |
| Oral cancer | *DKK2* | miR-21 | [3] |
| Oral cancer | *WNT7B* | miR-329 | [3] |
| Oral cancer | *WNT7B* | miR-410 | [3] |
| Osteosarcoma | *ZNRF3* | miR-146b-5p | [3] |
| Ovarian cancer (Mucinous) | *RNF43* | Frameshift / nonsense truncations | [39] |
| Ovarian cancer | *WIF1* | Promoter hypermethylation | [40] |
| Pancreatic cancer | *DKK1* | miR-29a | [3] |
| Pancreatic cancer | *FZD5* | miR-23a/b | [3] |
| Pancreatic cancer | *FZD7* | miR-23a/b | [3] |
| Hyperparathyroid cancer | *LRP5* | Aberrantly spliced internally deleted LRP5 receptor | [41] |
| PDAC | *RNF43* | Mutation; missense / truncating mutations | [42] |
| Prostate cancer | *AXIN1* | Truncating / missense mutation | [1, 2] |
| Prostate cancer | *AXIN2* | miR-34 | [3] |
| Prostate cancer | *CTNNB1* | miR-320 | [3] |
| Prostate cancer | *FZD7* | miR-613 | [3] |
| Prostate cancer | *LEF1* | miR-34 | [3] |
| Prostate cancer | *TCF1* | miR-34 | [3] |
| Prostate cancer | *TCF7* | miR-34 | [3] |
| Prostate cancer | *WNT5A* | miR-26a | [3] |
| Renal cancer | *CTNNB1* | miR-1826 | [3] |
| Renal cancer | *DKK2* | miR-1260b | [3] |
| Renal cancer | *SFRP1* | miR-1260b | [3] |
| RCC | *LATS1* | Promoter hypermethylation | [43] |
| Laryngeal squamous cell carcinoma (LSCC) | *APC* | miR-129-5p | [3] |
| TCLL | *WNT16* | miR-374b | [3] |
| Thyroid cancer | *ZNRF3* | miR-146b-5p | [3] |
| Wilms tumor | *FAM123B (WTX)* | Truncation / deletion | [44] |

**References**

[1] Xue C, Chu Q, Shi Q, et al. Wnt signaling pathways in biology and disease: mechanisms and therapeutic advances. Signal Transduct Target Ther. 2025;10:106. <https://doi.org/10.1038/s41392-025-02142-w>.

[2] Groenewald W, Lund AH, Gay DM. The Role of WNT Pathway Mutations in Cancer Development and an Overview of Therapeutic Options. Cells. 2023 Mar 24;12(7):990. <https://doi.org/10.3390/cells12070990>.

[3] Peng Y, Zhang X, Feng X, Fan X, Jin Z. The crosstalk between microRNAs and the Wnt/beta-catenin signaling pathway in cancer. Oncotarget. 2017 Feb 21;8(8):14089-14106. <https://doi.org/10.18632/oncotarget.12923>.

[4] Suzuki, H., Toyota, M., Carraway, H. et al. Frequent epigenetic inactivation of Wnt antagonist genes in breast cancer. Br J Cancer 98, 1147–1156 (2008). <https://doi.org/10.1038/sj.bjc.6604259>.

[5] Veeck, J., Wild, P.J., Fuchs, T. et al. Prognostic relevance of Wnt-inhibitory factor-1 (WIF1) and Dickkopf-3 (DKK3) promoter methylation in human breast cancer. BMC Cancer 9, 217 (2009). <https://doi.org/10.1186/1471-2407-9-217>.

[6] Chen S, Leng P, Guo J, et al. FBXW7 in breast cancer: mechanism of action and therapeutic potential. J Exp Clin Cancer Res. 2023;42:226. <https://doi.org/10.1186/s13046-023-02767-1>.

[7] Takahashi Y, Miyoshi Y, Takahata C, Irahara N, Taguchi T, Tamaki Y, Noguchi S. Down-regulation of LATS1 and LATS2 mRNA expression by promoter hypermethylation and its association with biologically aggressive phenotype in human breast cancers. Clin Cancer Res. 2005 Feb 15;11(4):1380-1385. <https://doi.org/10.1158/1078-0432.CCR-04-1773>.

[8] Song P, Gao Z, Bao Y, Chen L, Huang Y, Liu Y, Dong Q, Wei X. Wnt/beta-catenin signaling pathway in carcinogenesis and cancer therapy. J Hematol Oncol. 2024 Jun 18;17(1):46. <https://doi.org/10.1186/s13045-024-01563-4>.

[9] Moskalev EA, Luckert K, Vorobjev IA, Mastitsky SE, Gladkikh AA, Stephan A, et al. Concurrent epigenetic silencing of wnt/beta-catenin pathway inhibitor genes in B cell chronic lymphocytic leukaemia. BMC Cancer. 2012 Jun 6;12:213. <https://doi.org/10.1186/1471-2407-12-213>.

[10] Koinuma, K., Yamashita, Y., Liu, W. et al. Epigenetic silencing of AXIN2 in colorectal carcinoma with microsatellite instability. Oncogene 25, 139–146 (2006). <https://doi.org/10.1038/sj.onc.1209009>.

[11] Muto, Y., Maeda, T., Suzuki, K. et al. DNA methylation alterations of AXIN2 in serrated adenomas and colon carcinomas with microsatellite instability. BMC Cancer 14, 466 (2014). <https://doi.org/10.1186/1471-2407-14-466>.

[12] Aguilera, O., Fraga, M., Ballestar, E. et al. Epigenetic inactivation of the Wnt antagonist DICKKOPF-1 (DKK-1) gene in human colorectal cancer. Oncogene 25, 4116–4121 (2006). <https://doi.org/10.1038/sj.onc.1209439>.

[13] Chang CC, Lin HH, Lin JK, Lin CC, Lan YT, Wang HS, et al. FBXW7 mutation analysis and its correlation with clinicopathological features and prognosis in colorectal cancer patients. Int J Biol Markers. 2015 Feb 24;30(1):e88-e95. <https://doi.org/10.5301/jbm.5000125>.

[14] Wierzbicki PM, Adrych K, Kartanowicz D, et al. Underexpression of LATS1 TSG in colorectal cancer is associated with promoter hypermethylation. World J Gastroenterol. 2013 Jul 21;19(27):4363-4373. <https://doi.org/10.3748/wjg.v19.i27.4363>.

[15] Kleist B, Xu L, Kersten C, Seel V, Li G, Poetsch M. Single nucleotide polymorphisms of the adult intestinal stem cell marker Lgr5 in primary and metastatic colorectal cancer. Am J Transl Res. 2012;4(3):279-90.

[16] Su S, Hong F, Liang Y, Zhou J, Liang Y, Chen K, Wang X, Wang Z, Wang Z, Chang C, Han W, Gong W, Qin H, Jiang B, Xiong H, Peng L. Lgr5 Methylation in Cancer Stem Cell Differentiation and Prognosis-Prediction in Colorectal Cancer. PLoS One. 2015 Nov 24;10(11):e0143513. <https://doi.org/10.1371/journal.pone.0143513>.

[17] Seshagiri, S., Stawiski, E., Durinck, S. et al. Recurrent R-spondin fusions in colon cancer. Nature 488, 660–664 (2012). <https://doi.org/10.1038/nature11282>.

[18] Qi J, Zhu YQ, Luo J, Tao WH. Hypermethylation and expression regulation of secreted frizzled-related protein genes in colorectal tumor. World J Gastroenterol. 2006 Nov 28;12(44):7113-7. <https://doi.org/10.3748/wjg.v12.i44.7113>.

[19] Bass, A., Lawrence, M., Brace, L. et al. Genomic sequencing of colorectal adenocarcinomas identifies a recurrent VTI1A-TCF7L2 fusion. Nat Genet 43, 964–968 (2011). <https://doi.org/10.1038/ng.936>

[20] Jalilvand A, Soltanpour MS. Promoter Hypermethylation of Wnt/β-catenin Signaling Pathway Inhibitor WIF-1 Gene and its Association with MTHFR C677T Polymorphism in Patients with Colorectal Cancer. Oman Med J. 2020 Jun 25;35(3):e131. <https://doi.org/10.5001/omj.2020.49>.

[21] Jung YS, Jun S, Lee SH, Sharma A, Park JI. Wnt2 complements Wnt/β-catenin signaling in colorectal cancer. Oncotarget. 2015 Nov 10;6(35):37257-68. <https://doi.org/10.18632/oncotarget.6133>.

[22] Hibi K, Mizukami H, Goto T, Kitamura Y, Sakata M, Saito M, Ishibashi K, Kigawa G, Nemoto H, Sanada Y. WNT5A gene is aberrantly methylated from the early stages of colorectal cancers. Hepatogastroenterology. 2009 Jul-Aug;56(93):1007-9.

[23] Galamb O, Kalmár A, Péterfia B, Csabai I, Bodor A, Ribli D, Krenács T, Patai ÁV, Wichmann B, Barták BK, Tóth K, Valcz G, Spisák S, Tulassay Z, Molnár B. Aberrant DNA methylation of WNT pathway genes in the development and progression of CIMP-negative colorectal cancer. Epigenetics. 2016 Aug 2;11(8):588-602. <https://doi.org/10.1080/15592294.2016.1190894>.

[24] Yoshikawa H, Matsubara K, Zhou X, Okamura S, Kubo T, Murase Y, Shikauchi Y, Esteller M, Herman JG, Wei Wang X, Harris CC. WNT10B functional dualism: beta-catenin/Tcf-dependent growth promotion or independent suppression with deregulated expression in cancer. Mol Biol Cell. 2007 Nov;18(11):4292-303. <https://doi.org/10.1091/mbc.e06-10-0889>.

[25] Giannakis M, Hodis E, Jasmine Mu X, Yamauchi M, Rosenbluh J, Cibulskis K, Saksena G, Lawrence MS, Qian ZR, Nishihara R, Van Allen EM, Hahn WC, Gabriel SB, Lander ES, Getz G, Ogino S, Fuchs CS, Garraway LA. RNF43 is frequently mutated in colorectal and endometrial cancers. Nat Genet. 2014 Dec;46(12):1264-6. <https://doi.org/10.1038/ng.3127>.

[26] Wang, K., Yuen, S., Xu, J. et al. Whole-genome sequencing and comprehensive molecular profiling identify new driver mutations in gastric cancer. Nat Genet 46, 573–582 (2014). <https://doi.org/10.1038/ng.2983>.

[27] Min BH, Hwang J, Kim NKD, Park G, Kang SY, Ahn S, Ahn S, Ha SY, Lee YK, Kushima R, Van Vrancken M, Kim MJ, Park C, Park HY, Chae J, Jang SS, Kim SJ, Kim YH, Kim JI, Kim KM. Dysregulated Wnt signalling and recurrent mutations of the tumour suppressor RNF43 in early gastric carcinogenesis. J Pathol. 2016 Nov;240(3):304-314. <https://doi.org/10.1002/path.4777>.

[28] Wang O, Huang Y, Wu H, Zheng B, Lin J, Jin P. LncRNA LOC728196/miR-513c axis facilitates glioma carcinogenesis by targeting TCF7. Gene. 2018 Dec 30;679:119-125. <https://doi.org/10.1016/j.gene.2018.08.081>.

[29] Takagi H, Sasaki S, Suzuki H, Toyota M, Maruyama R, Nojima M, Yamamoto H, Omata M, Tokino T, Imai K, Shinomura Y. Frequent epigenetic inactivation of SFRP genes in hepatocellular carcinoma. J Gastroenterol. 2008;43(5):378-89. <https://doi.org/10.1007/s00535-008-2170-0>.

[30] Steinmann K, Sandner A, Schagdarsurengin U, Dammann RH. Frequent promoter hypermethylation of tumor-related genes in head and neck squamous cell carcinoma. Oncol Rep. 2009 Dec;22(6):1519-1526. <https://doi.org/10.3892/or_00000596>.

[31] Mullighan CG, Zhang J, Kasper LH, Lerach S, Payne-Turner D, Phillips LA, et al. CREBBP mutations in relapsed acute lymphoblastic leukaemia. Nature. 2011 Mar 10;471(7337):235-239. <https://doi.org/10.1038/nature09727>.

[32] Abrahamsson AE, Geron I, Gotlib J, Dao KH, Barroga CF, Newton IG, Giles FJ, Durocher J, Creusot RS, Karimi M, Jones C, Zehnder JL, Keating A, Negrin RS, Weissman IL, Jamieson CH. Glycogen synthase kinase 3beta missplicing contributes to leukemia stem cell generation. Proc Natl Acad Sci U S A. 2009 Mar 10;106(10):3925-9. <https://doi.org/10.1073/pnas.0900189106>.

[33] Pecina-Slaus N, Nikuseva Martic T, Tomas D, Beros V, Zeljko M, Cupic H. Meningiomas exhibit loss of heterozygosity of the APC gene. J Neurooncol. 2008 Mar;87(1):63-70. <https://doi.org/10.1007/s11060-007-9500-6>.

[34] Pecina-Slaus N, Kafka A, Vladusic T, Pecina HI, Hrascan R. AXIN1 Expression and Localization in Meningiomas and Association to Changes of APC and E-cadherin. Anticancer Res. 2016 Sep;36(9):4583-4594. <https://doi.org/10.21873/anticanres.11007>.

[35] Saydam O, Shen Y, Wurdinger T, Senol O, Boke E, James MF, et al. Downregulated microRNA-200a in meningiomas promotes tumor growth by reducing E-cadherin and activating the Wnt/beta-catenin signaling pathway. Mol Cell Biol. 2009;29(21):5923-5940. <https://doi.org/10.1128/MCB.00332-09>.

[36] Bukovac A, Dragicevic K, Kafka A, Oreskovic D, Cesarec-Augustinovic S, Pecina-Slaus N. Decoding the Role of DVL1 in Intracranial Meningioma. Int J Mol Sci. 2021 Nov 5;22(21):11996. <https://doi.org/10.3390/ijms222111996>.

[37] Vasudevan HN, Braunstein SE, Phillips JJ, Pekmezci M, Tomlin BA, Wu A, et al. Comprehensive molecular profiling identifies FOXM1 as a key transcription factor for meningioma proliferation. Cell Rep. 2018;22(13):3672-3683. <https://doi.org/10.1016/j.celrep.2018.03.013>.

[38] Maille E, Brosseau S, Hanoux V, Creveuil C, Danel C, Bergot E, et al. MST1/Hippo promoter gene methylation predicts poor survival in patients with malignant pleural mesothelioma in the IFCT-GFPC-0701 MAPS Phase 3 trial. Br J Cancer. 2019;120:387-397. <https://doi.org/10.1038/s41416-019-0379-8>.

[39] Ryland GL, Hunter SM, Doyle MA, Rowley SM, Christie M, Allan PE, Bowtell DD; Australian Ovarian Cancer Study Group; Gorringe KL, Campbell IG. RNF43 is a tumour suppressor gene mutated in mucinous tumours of the ovary. J Pathol. 2013 Feb;229(3):469-76. <https://doi.org/10.1002/path.4134>.

[40] Chmelarova M, Dvorakova E, Spacek J, Laco J, Mzik M, Palicka V. Promoter methylation of GATA4, WIF1, NTRK1 and other selected tumour suppressor genes in ovarian cancer. Folia Biol (Praha). 2013;59(2):87-92. <https://doi.org/10.14712/fb2013059020087>.

[41] Björklund P, Akerström G, Westin G. An LRP5 receptor with internal deletion in hyperparathyroid tumors with implications for deregulated WNT/beta-catenin signaling. PLoS Med. 2007 Nov 27;4(11):e328. <https://doi.org/10.1371/journal.pmed.0040328>.

[42] Jiang X, Hao HX, Growney JD, Woolfenden S, Bottiglio C, Ng N, Lu B, Hsieh MH, Bagdasarian L, Meyer R, Smith TR, Avello M, Charlat O, Xie Y, Porter JA, Pan S, Liu J, McLaughlin ME, Cong F. Inactivating mutations of RNF43 confer Wnt dependency in pancreatic ductal adenocarcinoma. Proc Natl Acad Sci U S A. 2013 Jul 30;110(31):12649-54. <https://doi.org/10.1073/pnas.1307218110>.

[43] Chen KH, He J, Wang DL, Cao JJ, Li MC, Zhao XM, Sheng X, Li WB, Liu WJ. Methylation-associated inactivation of LATS1 and its effect on demethylation or overexpression on YAP and cell biological function in human renal cell carcinoma. Int J Oncol. 2014;45:2511-2521. <https://doi.org/10.3892/ijo.2014.2687>.

[44] Ruteshouser EC, Robinson SM, Huff V. Wilms tumor genetics: mutations in WT1, WTX, and CTNNB1 account for only about one-third of tumors. Genes Chromosomes Cancer. 2008 Jun;47(6):461-470. <https://doi.org/10.1002/gcc.20553>.
